# Orally available Colivin traps TRPV3 in an inactive state and suppresses intestinal inflammation

**DOI:** 10.1038/s44318-026-00896-9

**Published:** 2026-08-13

**Authors:** Yujing Wang, Jiahui Chen, Qinglian Tang, Xu Qin, Shuxian Wang, Chuchu Xi, Zhaowen Lu, Kerui Ren, Chu Xue, Jianhua Shen, Kai Wang, Michael X Zhu, Jie Yu, Zhengyu Cao

**Affiliations:** 1 State Key Laboratory of Natural Medicines and Jiangsu Provincial Key Laboratory for TCM Evaluation and Translational Research, School of Traditional Chinese Pharmacy, China Pharmaceutical University, Nanjing, China; 2 Interdisciplinary Research Center on Biology and Chemistry, Shanghai Institute of Organic Chemistry, Chinese Academy of Sciences, Shanghai, China; 3 University of Chinese Academy of Sciences, Beijing, China; 4 State Key Laboratory of Drug Research, Shanghai Institute of Materia Medica, Chinese Academy of Sciences, Shanghai, China; 5 Institute of Molecular Physiology, Shenzhen Bay Laboratory, Shenzhen, China

**Keywords:** Digestive System, Membranes & Trafficking, Pharmacology & Drug Discovery

## Abstract

Transient receptor potential vanilloid 3 (TRPV3) is a non-selective cation channel highly expressed in the skin and intestine. While its roles in itch and skin inflammation are established, the physiological role of TRPV3 in the intestine remains relatively poorly understood. Topical application of a TRPV3 inhibitor, KM-001, has entered phase I clinical trials for the treatment of pruritus; however, its poor metabolic stability limits broader therapeutic application. Here, we show that the KM-001-derivative Colivin has comparable TRPV3-blocking activity, but enhanced metabolic stability and increased oral bioavailability. Cryo-EM and site-directed mutagenesis analyses established that Colivin binds to the vanilloid binding pocket of TRPV3, stabilizing one of two distinct non-conducting conformations. Oral administration of Colivin effectively suppressed DSS-induced ulcerative colitis (UC) in wild-type, but not Trpv3-deficient mice. Collectively, our findings establish TRPV3 inhibition as a novel therapeutic strategy for UC, offer key structural insights into the inhibition mechanism of TRPV3, and provide critical structural insights for the rational design of potent and selective TRPV3 inhibitors.

## Synopsis

TRPV3 drives itch and inflammation in the skin, but is also widely expressed in the intestine where its function is unclear. This study identifies orally available Colivin as a TRPV3 inhibitor and key regulator of intestinal inflammation, suggesting intestinal TRPV3 inhibition as a promising therapeutic strategy for ulcerative colitis.

- Colivin, an optimized KM-001 derivative, maintains potent TRPV3 inhibition with enhanced metabolic stability and oral bioavailability.
- Colivin targets the TRPV3 vanilloid pocket and stabilizes two distinct non-conducting conformations, providing a structural basis for future TRPV3 inhibitor development.
- Oral Colivin administration effectively reduces DSS-induced ulcerative colitis in wild-type but not *Trpv3*^-/-^ mice.

## Abstract (web summary)

Structural trapping reveals TRPV3 to be a druggable driver of intestinal inflammation.

## Graphical abstract

**Figure:**
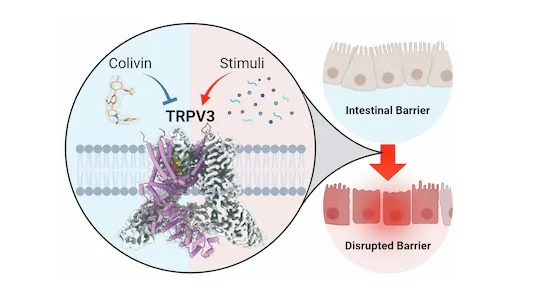

## Introduction

Ulcerative colitis (UC) is a remission-relapsing inflammatory bowel disease characterized by recurrent diarrhea, rectal bleeding, abdominal pain, and cramping (Gros and Kaplan, 2023; Le Berre et al, 2023). UC disrupts colonic mucosal integrity through sustained immune dysregulation, leading to an increased long-term risk of colorectal cancer (Schiavone et al, 2025). The average morbidity of UC is about 0.14–0.25%, depending on geographic locations (Ananthakrishnan, 2015). With the current treatments, managing UC remains a significant clinical challenge. Conventional drugs (aminosalicylates, corticosteroids, immunosuppressants) are often ineffective for long-term remission, while biologics carry risks of infection and are cost-prohibitive. Moreover, the persistent 15% colectomy rate highlights the inadequacy of existing strategies (Ungaro et al, 2017; Wangchuk et al, 2024). Thus, this critical unmet clinical need underscores the urgency of discovering novel molecular targets, a fundamental step toward developing UC therapeutics.

Transient receptor potential vanilloid 3 (TRPV3) is a warm-sensitive Ca^2+^-permeable non-selective cation channel abundantly expressed in the skin, nervous, and gastrointestinal systems (Peier et al, 2002; Ueda et al, 2009; Xu et al, 2002). Since the discovery that TRPV3 gain-of-function mutants, including Gly573Ser and Gly573Cys, are linked to Olmsted syndrome, a rare congenital cutaneous inflammatory disease (Lin et al, 2012), the functional role of TRPV3 has been widely studied in the skin system. TRPV3 has been demonstrated to regulate keratinocyte proliferation, differentiation, and proinflammation, thereby influencing the formation of the cutaneous epidermal barrier (Cheng et al, 2010; Szöllősi et al, 2018; Wang et al, 2020) and cutaneous inflammation (Cui et al, 2018; Wang et al, 2024). In addition to regulating skin inflammation and barrier formation, TRPV3 has also been reported to regulate itch and pain sensation due to its expression in sensory neurons (Klein et al, 2014; Xu et al, 2002).

TRPV3 has also been demonstrated to be abundantly expressed in the gastrointestinal system (Ueda et al, 2009). Cannabichromene, a natural cannabinoid, was found to elicit TRPV3-dependent Ca^2+^ elevation in intestinal epithelial cells, suggesting the functional expression of TRPV3 in these cells (De Petrocellis et al, 2012). However, except for a few reports suggesting the involvement of intestinal TRPV3 in ammonium uptake (Liebe et al, 2021; Manneck et al, 2021), the (patho)physiological role of TRPV3 in the gastrointestinal system remains unclear.

The critical involvement of TRPV3 in skin inflammation has sparked significant interest in discovering its inhibitors. This has led to the identification of several natural compounds (such as osthole, scutellarein, and isochlorogenic acid) (Qi et al, 2022; Sun et al, 2018; Wang et al, 2022) and synthetic molecules (for example: (pyridin-2-yl)methanol derivative 74a, cinnamate ester derivative 8c, and Trpvicin (Black et al, 2016; Gomtsyan et al, 2016; Lv et al, 2021)). However, these compounds lack either high affinity or selectivity for TRPV3, or they display poor oral bioavailability, preventing their systemic use to explore the pathophysiological role of TRPV3 and clinical applications. Among them, a picolinamide derivative, KM-001 ((3-(2’-cyclopropyl-3-(hydroxymethyl)-[1,1’-biphenyl]-4-yl)pyrrolidin-1-yl)(5-fluoropyridin-2-yl)methanone), has been reported to be a TRPV3 inhibitor. Topical use of KM-001 targeting TRPV3 for treating pruritus in patients with lichen simplex chronicus has completed a Phase I clinical trial, showing efficacious improvement of itching (Barak et al, 2023; Braiman-Wiksman et al, 2023). However, its poor oral availability makes it unsuitable for systemic use and for investigations into the therapeutic potential of TRPV3 in diseases beyond those affecting the skin (Wu et al, 2021).

In this study, we developed Colivin ((3-(4’-fluoro-3-(hydroxymethyl-*d*_2_)-2’-(trifluoromethoxy)-[1,1’-biphenyl]-4-yl)pyrrolidin-1-yl)(5-fluoropyridin-2-yl)methanone), a selective and potent TRPV3 inhibitor with improved metabolic stability and oral bioavailability, based on the structure of KM-001. Cryo-EM analysis revealed that Colivin binds the TRPV3 vanilloid pocket, yielding two distinct non-conducting conformations, offering novel structural insights into the mechanism of TRPV3 inhibition and a framework for the structure-based rational design of more potent TRPV3 inhibitors. Importantly, Colivin treatment mitigated intestinal inflammation in a dextran sulfate sodium (DSS)-induced mouse colitis model, an effect entirely dependent on TRPV3. These findings establish proof of concept for TRPV3 as a promising therapeutic target for UC.

## Results

### Colivin is a novel, selective TRPV3 inhibitor with good oral availability

**Figure 1:**
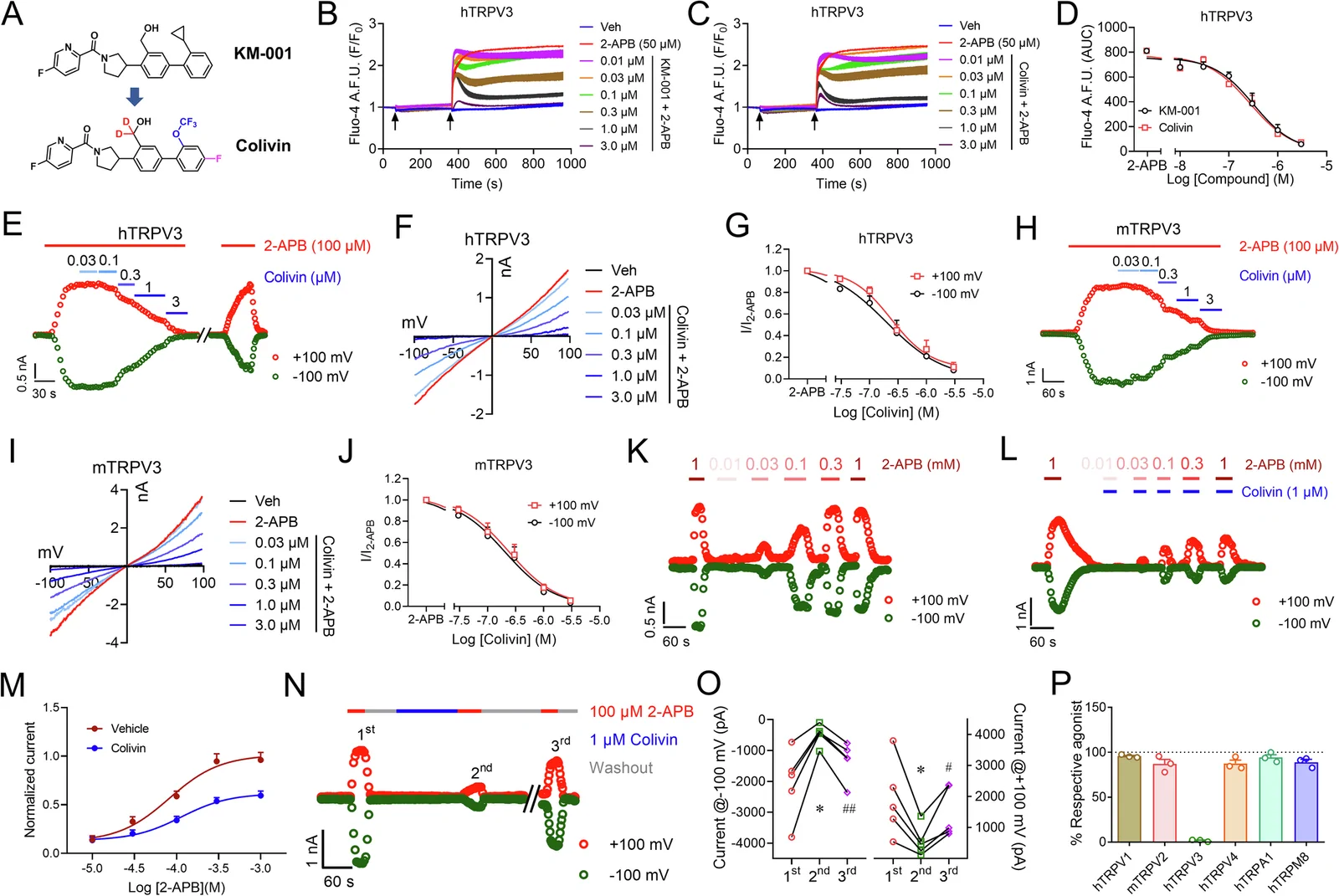
Colivin is a novel TRPV3 inhibitor. (**A**) Structures of the original lead, KM-001, and the newly synthesized selective TRPV3 antagonist, Colivin. (**B**, **C**) Representative time courses of Fluo-4 fluorescence changes (arbitrary fluorescence unit, A.F.U.) reflecting 2-APB-induced [Ca^2+^]_i_ elevation in HEK-293 cells that expressed hTRPV3 in the presence of varying concentrations of KM-001 (**B**) and Colivin (**C**). KM-001 and Colivin were administered at concentrations of 0.01–3 μM, at 60 s (first arrow). Subsequently, the TRPV3 agonist 2-APB (50 μM) was added at 360 s (second arrow). (**D**) Concentration-response curves for KM-001 and Colivin suppression of TRPV3-mediated Ca^2+^ responses. Each data point represents mean ± SEM (*N* = 3 wells). (**E**, **H**) Effect of Colivin (0.03–3 µM as indicated by blue bars) on 2-APB (100 μM)-evoked hTRPV3 (**E**) and mTRPV3 (**H**) whole-cell currents at ±100 mV. (**F**, **I**) Current-voltage curves of 2-APB-induced hTRPV3 (**F**) and mTRPV3 (**I**) currents in the absence and presence of the indicated concentrations of Colivin, derived from (**E**, **H**). (**G**, **J**) Concentration-response curves for Colivin inhibition of hTRPV3 (**G**) and mTRPV3 (**J**)-mediated whole-cell currents. Each data point represents mean ± SEM (*N* = 5–6 cells). (**K**, **L**) Whole-cell hTRPV3 currents at ±100 mV evoked by the 2-APB (0.01–1 mM) in the absence (**K**) and presence (**L**) of Colivin (1 μM). (**M**) Concentration-response curves of 2-APB-induced hTRPV3 currents at +100 mV in the absence and presence of Colivin. The currents were normalized to the maximal current evoked by 1 mM 2-APB in the absence of Colivin for each cell. Each data point represents mean ± SEM (*N* = 5 cells). (**N**) Whole-cell hTRPV3 currents at ±100 mV recorded during sequential perfusion with 2‑APB (100 μM) and Colivin (1 μM), as indicated. (**O**) Quantification of the current amplitude evoked by sequential applications of 2-APB. Each connected line (black) represents a dataset from the same cell (*N* = 5 cells). *P < 0.05, *vs*. first application of 2-APB; ^#^*P* < 0.05, ^##^*P* < 0.01, *vs*. second application of 2-APB, by one-way ANOVA followed with Dunnett’s multiple comparisons tests. Exact *P* values for all comparisons are listed in Dataset EV1. (**P**) Quantification of the effect of Colivin (10 μM) on [Ca^2+^]_i_ elevations mediated by hTRPV1, mTRPV2, hTRPV3, hTRPV4, hTRPA1, and hTRPM8 in response to capsaicin (1 µM), 2-APB (100 µM), 2-APB (50 µM), GSK1016790A (10 nM), AITC (30 µM), and menthol (300 µM), respectively. Bars represent mean ± SEM (*N* = 3 wells) of Ca^2+^ responses in the presence of 10 μM Colivin, normalized to that with the respective agonist alone. Source data are available online for this figure.

KM-001 (Fig. 1A, top), a 2-pyridinecarboxamide derivative, is a TRPV3 inhibitor currently undergoing Phase I clinical trials for the treatment of pruritus in patients with lichen simplex chronicus (Barak et al, 2023). However, it has been reported to have poor oral availability (Wu et al, 2021), limiting its use to topical applications only. We confirmed KM-001 to be a potent TRPV3 inhibitor by using a fluorescence-based Ca^2+^ flux assay, which showed that KM-001 suppressed 2-APB (50 μM)-induced Ca^2+^ influx in HEK-293 cells transiently transfected with hTRPV3 with an IC_50_ value of 0.26 ± 0.04 μM (Fig. 1B,D). However, the half-life (*t*_1/2_) values of KM-001 in human and mouse liver microsomes were only 13 and 2 min, respectively (Table 1), suggesting that it is metabolically unstable, which may explain its poor oral availability (Wu et al, 2021).

**Table 1:** The metabolic profile of KM001 and Colivin in mouse and human liver microsomes.

Test Compound	Species	T_1/2_ (min)	Cl_Hep_ (mL/min/kg)
KM001	Mouse	2.24	86.79
	Human	12.5	1343
Colivin	Mouse	12.77	74.34
	Human	70.8	903

*HBF* hepatic blood flow, *V* The incubation volume, *BW* body weight.

Hepatic clearance: Cl_Hep_ = HBF × Cl/(HBF + Cl).

Clearance: Cl = 0.693 × V/T_1/2_/BW.

To develop orally available TRPV3 inhibitors, we designed and synthesized a series of KM-001 derivatives. As part of our structural optimization strategy, deuterium and fluorine atoms were introduced to metabolically labile positions. This approach led to the discovery of a novel compound, Colivin (Fig. 1A, bottom). Fluorescence-based Ca^2+^ influx assay showed that Colivin potently suppressed hTRPV3 activity with IC_50_ values of 0.23 ± 0.02 μM (Fig. 1C,D). Moreover, compared to KM-001, the *t*_1/2_ values of Colivin in human and mouse liver microsomes were markedly increased to 71 and 13 min, respectively (Table 1), demonstrating improved metabolic stability. Oral administration of Colivin (10 mg/kg) to mice resulted in rapid and abundant absorption, showing a maximum concentration (*C*_max_) of 1147 ng/mL and a time-to-peak concentration (*t*_max_) of 0.25 h. The *t*_1/2_ of Colivin was 1.80 h, which is considered an intermediate value in mice (Table 2). In agreement with the Ca^2+^ influx assay, whole-cell recording demonstrated that Colivin concentration-dependently suppressed 2-APB (100 μM)-induced outward and inward currents in HEK-293 cells transiently expressing hTRPV3 with IC_50_ values of 0.24 ± 0.01 μM and 0.20 ± 0.03 μM at +100 and −100 mV, respectively (Fig. 1E–G). In HEK-293 cells transiently expressing mTRPV3, Colivin yielded similar IC_50_ values of 0.24 ± 0.03 μM and 0.20 ± 0.02 μM at +100 and −100  mV, respectively (Fig. 1H–J). This suggests that Colivin inhibits hTRPV3 and mTRPV3 equally well.

**Table 2:** Pharmacokinetic parameters of Colivin after intragastric administration (10 mg/kg) in ICR mice.

Mice	*t*_max_ (h)	*C*_max_ (ng/mL)	AUC_0-t_ (h*ng/mL)	AUC_0-∞_ (h*ng/mL)	MRT_0-∞_ (h)	*t*_1/2_ (h)
Mean ± SEM	0.33 ± 0.08	1147 ± 77	2318 ± 103	2333 ± 106	2.04 ± 0.06	1.80 ± 0.07

While Colivin caused a slight rightward shift in the EC_50_ value for 2-APB-evoked hTRPV3 currents (from 67.50 ± 13.44 μM without Colivin to 104.50 ± 19.23 μM with Colivin), it strongly suppressed the maximal current amplitude (Fig. 1K–M), suggesting that Colivin acts as a non-competitive inhibitor of TRPV3. Furthermore, although Colivin did not alter basal hTRPV3 currents, preincubation with Colivin suppressed 2-APB response by 71.3 ± 14.1% and 77.0 ± 9.3% ( + 100 mV and −100 mV, respectively), suggesting that Colivin does not rely on channel activation to exert an inhibitory effect on hTRPV3 (Fig. 1N,O). Then, following extended washing, the response to 2-APB largely recovered, reaching 77.3 ± 29.3% and 64.2 ± 20.6% ( + 100 mV and −100 mV, respectively) of the initial current amplitudes, indicating that the inhibition by Colivin is reversible (Fig. 1N,O).

**Figure EV1:**
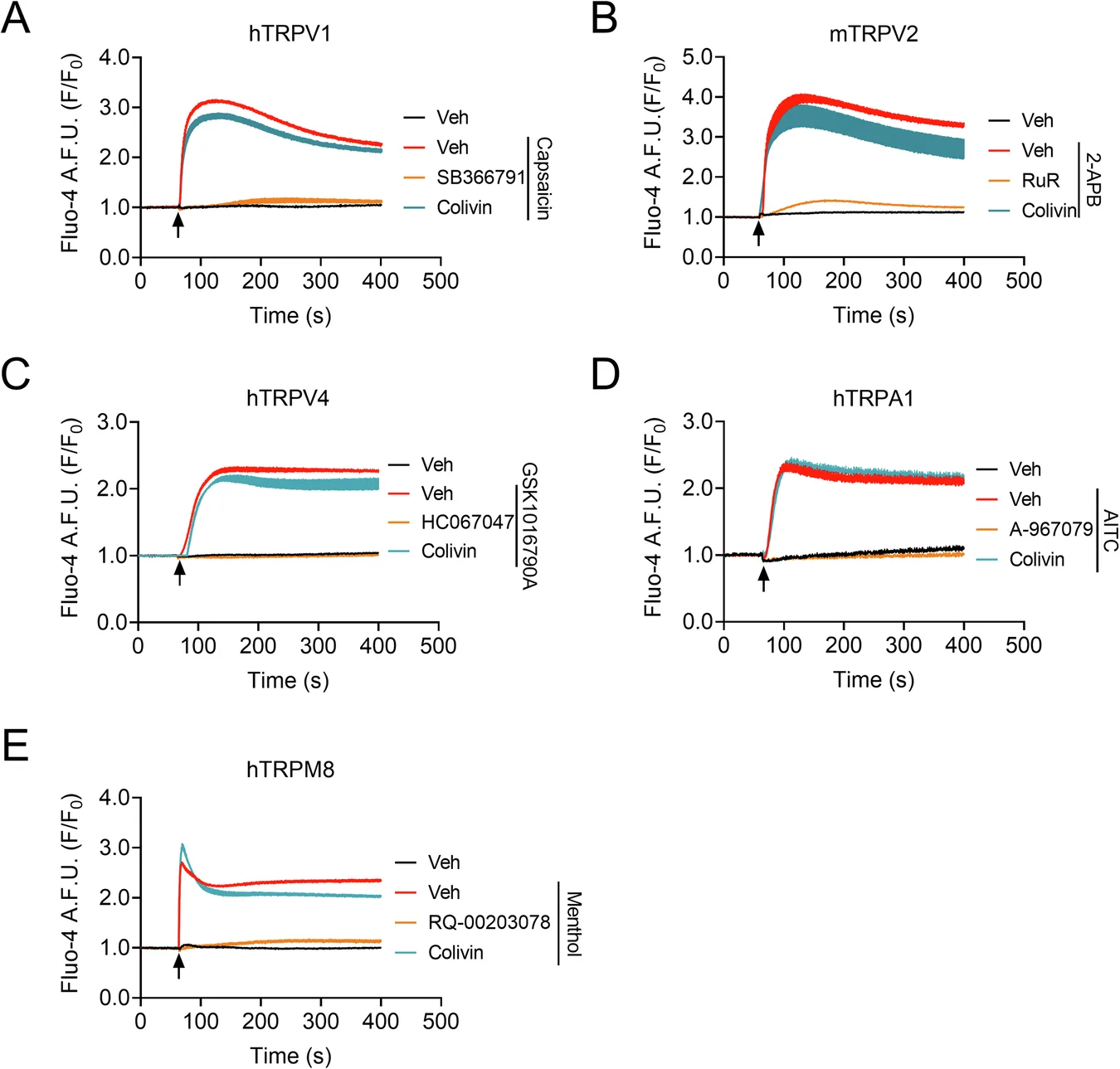
Effects of Colivin on thermosensitive TRP channels. Time courses of Ca^2+^ influx induced by capsaicin (1 μM), 2-APB (100 μM), GSK1016790A (10 nM), AITC (30 μM), and menthol (300 μM) in HEK-293 cells expressing hTRPV1 (**A**), mTRPV2 (**B**), hTRPV4 (**C**), hTRPA1 (**D**), and hTRPM8 (**E**), respectively. Colivin (10 μM), along with the corresponding inhibitors: SB366791 (1 μM), ruthenium red (30 μM), HC067047 (1 μM), A-967079 (1 μM), and RQ-00203078 (1 μM), was incubated for 5 min, followed by the addition of agonists at 60 s (black arrow). Each data point represents mean ± SEM (*N* = 3 wells). Each experiment was repeated in three independent cultures with similar results. Related to Fig. 1. Source data are available online for this figure.

To evaluate the selectivity of Colivin among thermosensitive TRP channels, we investigated the effect of 10 μM Colivin on Ca^2+^ influx mediated by hTRPV1, mTRPV2, hTRPV4, hTRPA1, and hTRPM8, respectively. Colivin had no significant effect on these thermosensitive TRP channels, demonstrating its specificity on TRPV3 (Figs. 1P and EV1). Together, these data demonstrate that Colivin is a potent, selective, non-competitive, and orally available TRPV3 inhibitor.

### Colivin binds to the vanilloid site

**Figure EV2:**
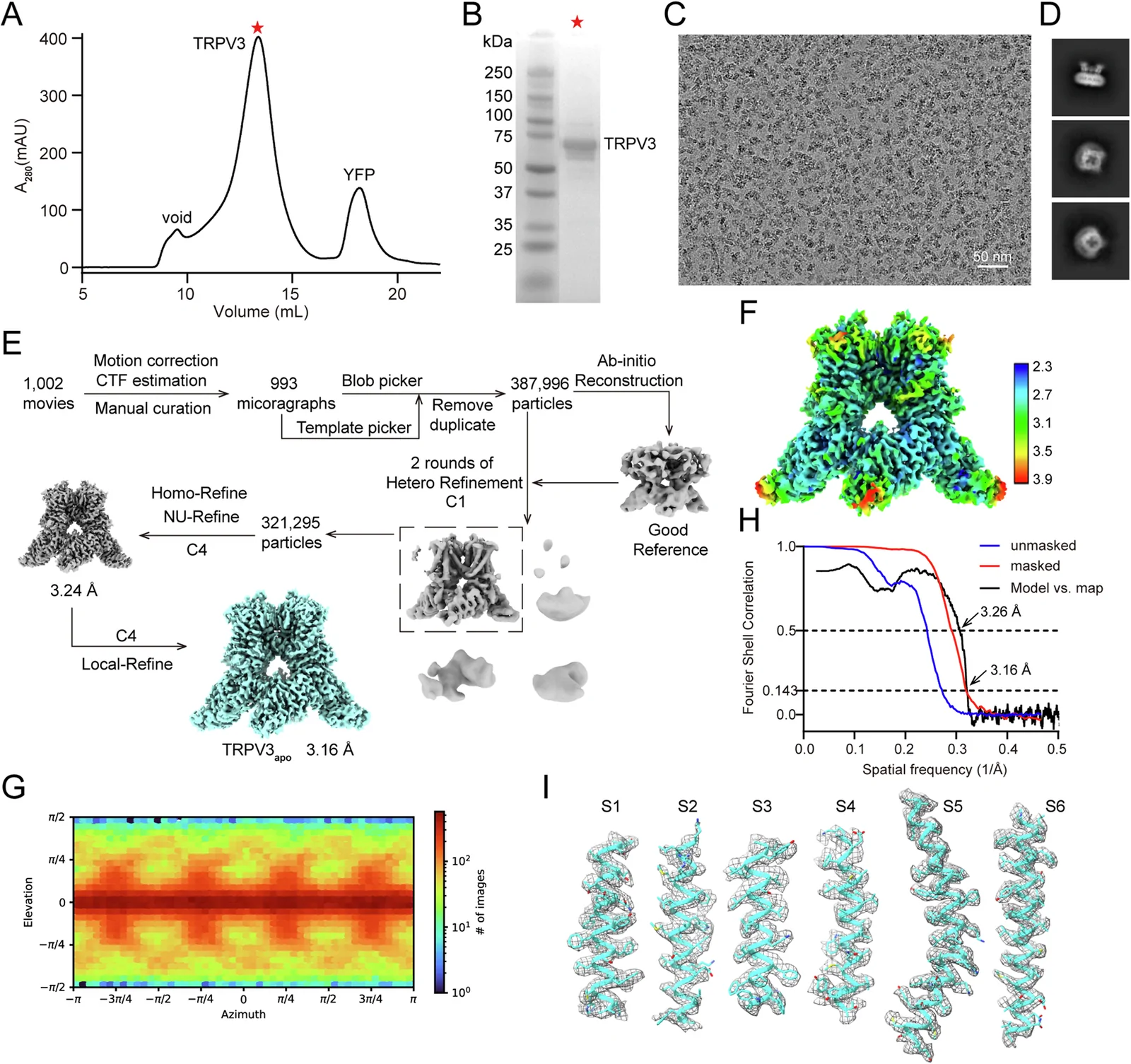
Protein purification and Cryo-EM reconstruction of TRPV3_apo_. (**A**) Size-exclusion chromatography of purified human TRPV3. The fractions were pooled for cryo-EM sample preparation. (**B**) SDS-PAGE gel of purified human TRPV3. (**C**) A representative motion-corrected micrograph of the dataset. Scale bar = 50 nm. (**D**) Representative 2D class averages generated from particles for the TRPV3_apo_ map. (**E**) Flow chart of cryo-EM data processing of TRPV3_apo_. Further details can be found in Methods. The contour level of the final map is 0.25. (**F**) Local resolution map of TRPV3_apo_, colored according to the estimated values ranging from 2.3 to 3.9 Å. (**G**) Particle angular distribution calculated in cryoSPARC for the final reconstruction. (**H**) Gold standard Fourier Shell Correlations (FSC) curves of unmasked map (blue), masked map (red), and model versus map (black), marked with resolutions corresponding to FSC  =  0.5 and 0.143. (**I**) Representative cryo-EM densities of transmembrane helices (S1-S6) of TRPV3_apo_, contoured at 0.25. Related to Fig. 2.

**Figure 2:**
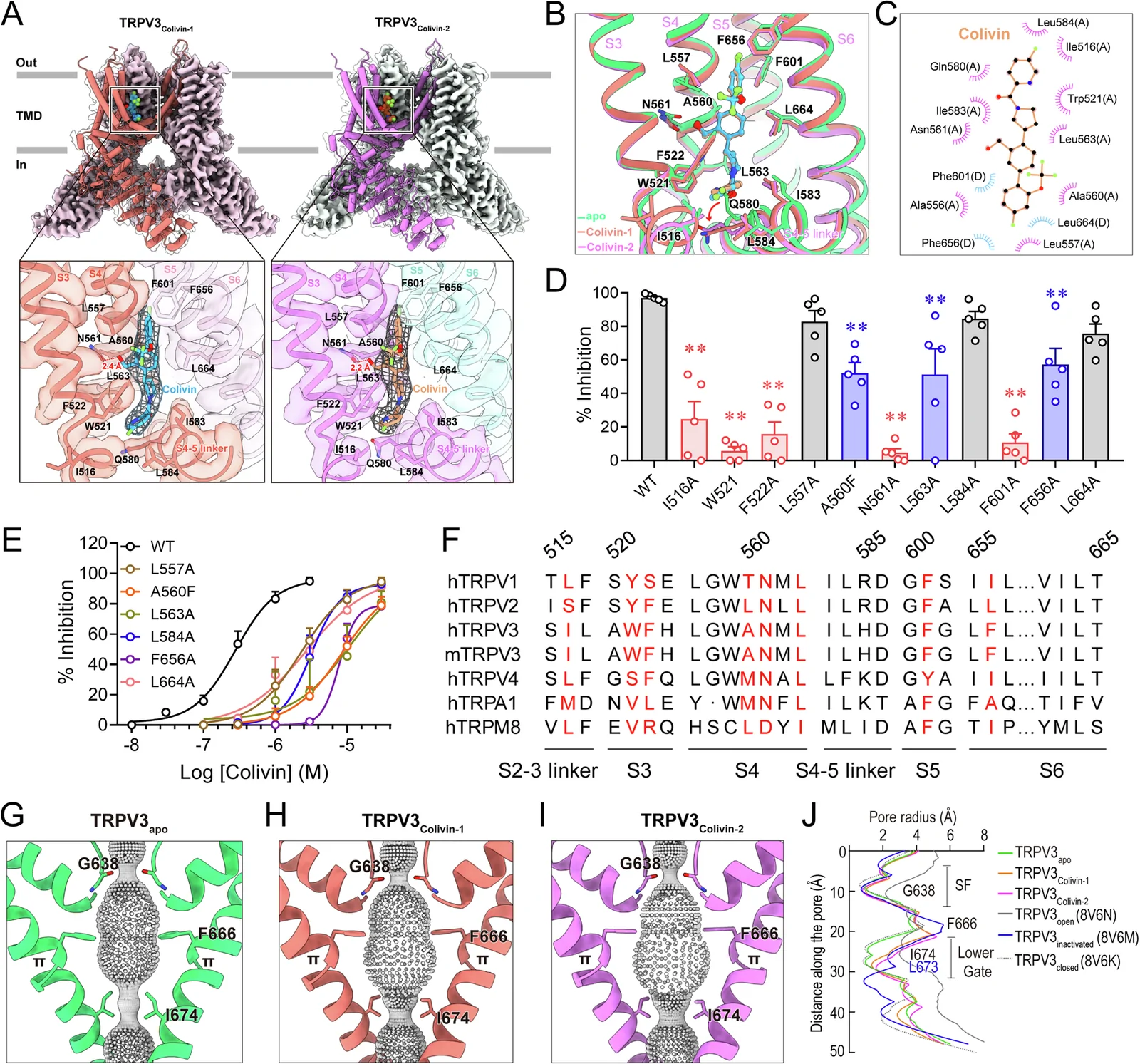
Colivin binds to the vanilloid site of hTRPV3. (**A**) Structures of TRPV3_Colivin-1_ (deep skyblue) and TRPV3_Colivin-2_ (wheat) tetramers determined by single-particle cryo-EM, with the Colivin-binding sites enlarged in the boxes. One subunit is shown in light salmon or violet cartoon, with a transparent surface, while the other three are displayed in a density map colored in pink or cyan, contoured at 0.25. Cryo-EM densities for Colivin are shown as mesh in the expanded boxes. The hydrogen bonds between the carboxamide group of Asn^561^ and hydroxyl group of Colivin are shown as red dashed lines, with distances marked below. (**B**) Superimposed structures of TRPV3_apo_, TRPV3_Colivin-1_, and TRPV3_Colivin-2_, showing only the vanilloid site. The movement of the side chain of Gln580 is indicated by the red arrow. (**C**) Schematic showing the interactions between Colivin and residues in the TRPV3 vanilloid pocket, generated from LigPlot. (**D**) Summary of Colivin (10 μM) inhibition (%) of 2-APB-induced currents at +100 mV in HEK-293 cells expressing WT hTRPV3 and the indicated hTRPV3 mutants. Blue and red bars indicate mutations that displayed greater than 50% and less than 30%, respectively, inhibition by Colivin. Data represent the mean ± SEM from 5 cells. **P < 0.01, *vs*. WT, by one-way ANOVA with Dunnett’s multiple comparisons tests. Exact *P* values for all comparisons are listed in Dataset EV1. (**E**) Concentration-response curves of Colivin inhibition of WT hTRPV3 and its mutants. Data represent the mean ± SEM from five cells. (**F**) Sequence alignment of human thermosensitive TRP and mTRPV3 channels in the vanilloid site. Residues involved in Colivin binding are highlighted in red. (**G**–**I**) Ion-conduction pores of TRPV3_apo_ (**G**), TRPV3_Colivin-1_ (**H**), and TRPV3_Colivin-2_ (**I**), calculated with HOLE and shown as gainsboro spheres. Only two opposing subunits of the pore domains are shown for clarity. Gating residues at selectivity filters and lower gates are labeled. (**J**) Pore radii along the permeation pathways of TRPV3_apo_, TRPV3_Colivin-1_, TRPV3_Colivin-2_, TRPV3_closed_ (PDB ID: 8V6K), TRPV3_inactivated_ (PDB ID: 8V6M) and TRPV3_open_ (PDB ID: 8V6N), calculated with HOLE. Two constriction sites of the selectivity filters and the activation gates are shown as SF and Lower Gate. Source data are available online for this figure.

**Figure EV3:**
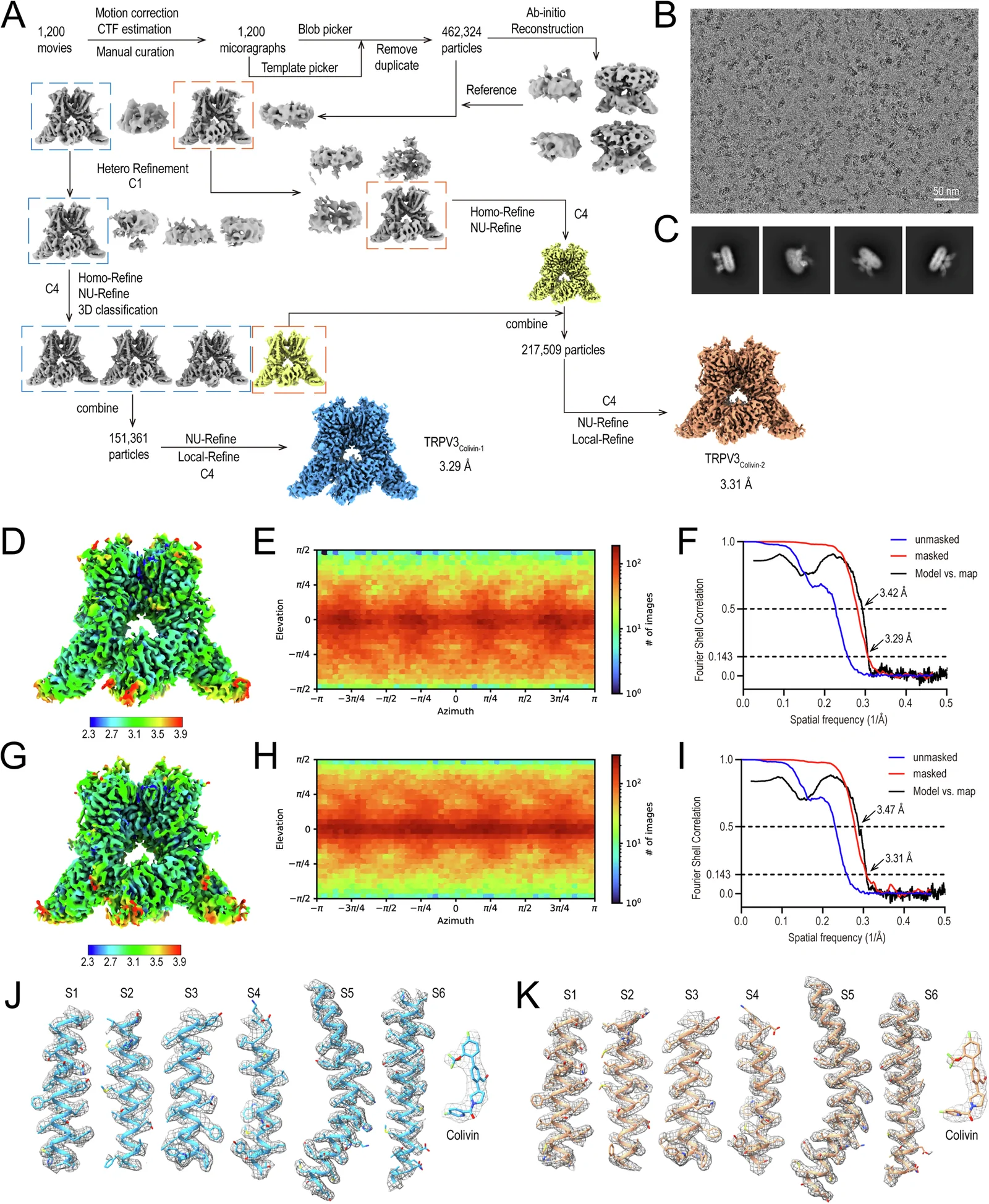
Cryo-EM reconstruction of TRPV3_Colivin_. (**A**) Flow chart of cryo-EM data processing of TRPV3_Colivin_. Further details can be found in “Methods”. The contour level of the final maps is 0.3. (**B**) A representative motion-corrected micrograph of the dataset. Scale bar = 50 nm. (**C**) Representative 2D class averages generated from particles. (**D**, **G**) Local resolution map of TRPV3_Colivin-1_ (**D**) and TRPV3_Colivin-2_ (**G**), colored according to the estimated values ranging from 2.3 to 3.9 Å. (**E**, **H**) Particle angular distributions calculated in cryoSPARC for the final reconstruction of TRPV3_Colivin-1_ (**E**) and TRPV3_Colivin-2_ (**H**). (**F**, **I**) FSC curves of unmasked map (blue), masked map (red), and model versus map (black) for the final map of TRPV3_Colivin-1_ (**F**) and TRPV3_Colivin-2_ (**I**), marked with resolutions corresponding to FSC  =  0.5 and 0.143. (**J**, **K**) Representative cryo-EM densities of transmembrane helices (S1–S6) and ligands for TRPV3_Colivin-1_ (**J**) and TRPV3_Colivin-2_ (**K**), contoured at 0.3. Related to Fig. 2.

To examine the mechanism of TRPV3 inhibition by Colivin, we determined high-resolution structures of TRPV3 in its apo state and in complex with a saturating concentration of Colivin (1 mM). Cryo-EM analysis revealed a single conformation of apo TRPV3 (TRPV3_apo_) at a resolution of 3.16 Å (Fig. EV2; Table 3), and two distinct Colivin-bound conformations (TRPV3_Colivin-1_ and TRPV3_Colivin-2_) at resolutions of 3.29 Å and 3.31 Å, respectively (Figs. 2A and EV3; Table 3). Unlike TRPV3_apo_, both TRPV3_Colivin-1_ and TRPV3_Colivin-2_ displayed well-defined non-protein densities within the vanilloid binding pocket formed by the S3, S4, and S4-S5 linker of one subunit and the S5 and S6 helices of the neighboring subunit that were unambiguously assigned to Colivin (Fig. 2A). Notably, Colivin adopts a similar binding pose in both TRPV3_Colivin__-__1_ and TRPV3_Colivin-2_ (Figs. 2A, Zoom-in and EV3J,K). Superimposing the vanilloid binding pockets in TRPV3_Colivin_ and TRPV3_apo_ revealed that the binding of Colivin induced an inward movement of the S4-S5 linker toward the channel pore and a displacement of S6 helix, resulting in an expansion of the binding pocket. Noticeably, the side chain of Gln580 in the S4-S5 linker swings away from the binding site to accommodate Colivin binding (Fig. 2B, red arrowhead).

**Table 3:** Statistics of 3D reconstructions and model refinement.

Protein	TRPV3_apo_	TRPV3_Colivin-1_	TRPV3_Colivin-2_
**Data collection and processing**			
Magnification	81 K	81 K	81 K
Voltage (kV)	300	300	300
Electron exposure (e^–^/Å^2^)	50	50	50
Defocus range (μm)	−1.0 ~ −2.0	−1.0 ~ −2.0	−1.0 ~ −2.0
Magnified pixel size (Å)	1.055	1.055	1.055
Micrographs	1002	1200	1200
Initial particle images (no.)	878,678	856,086	856,086
Final particle images (no.)	321,295	151,361	217,509
Symmetry	C4	C4	C4
Map Resolution (Å)	3.16	3.29	3.31
Sharpening B-factor (Å^2^)	120.6	123.8	127.4
**Model statistics**			
Map CC	0.78	0.75	0.75
Number of atoms	20,492	20,712	20,440
Protein residues	2516	2536	2516
Ligands	0	4	4
**R.m.s. deviations**			
Bond length (Å)	0.002	0.002	0.002
Bond angle (°)	0.393	0.443	0.501
**Ramachandran plot**			
Favored (%)	98.15	96.87	97.15
Allowed (%)	1.85	3.13	2.85
Disallowed (%)	0.00	0.00	0.00
Rotamers outlier (%)	0.00	0.00	0.00
MolProbity score	1.38	1.45	1.55
Clashscore	6.91	5.01	7.29

**Figure EV4:**
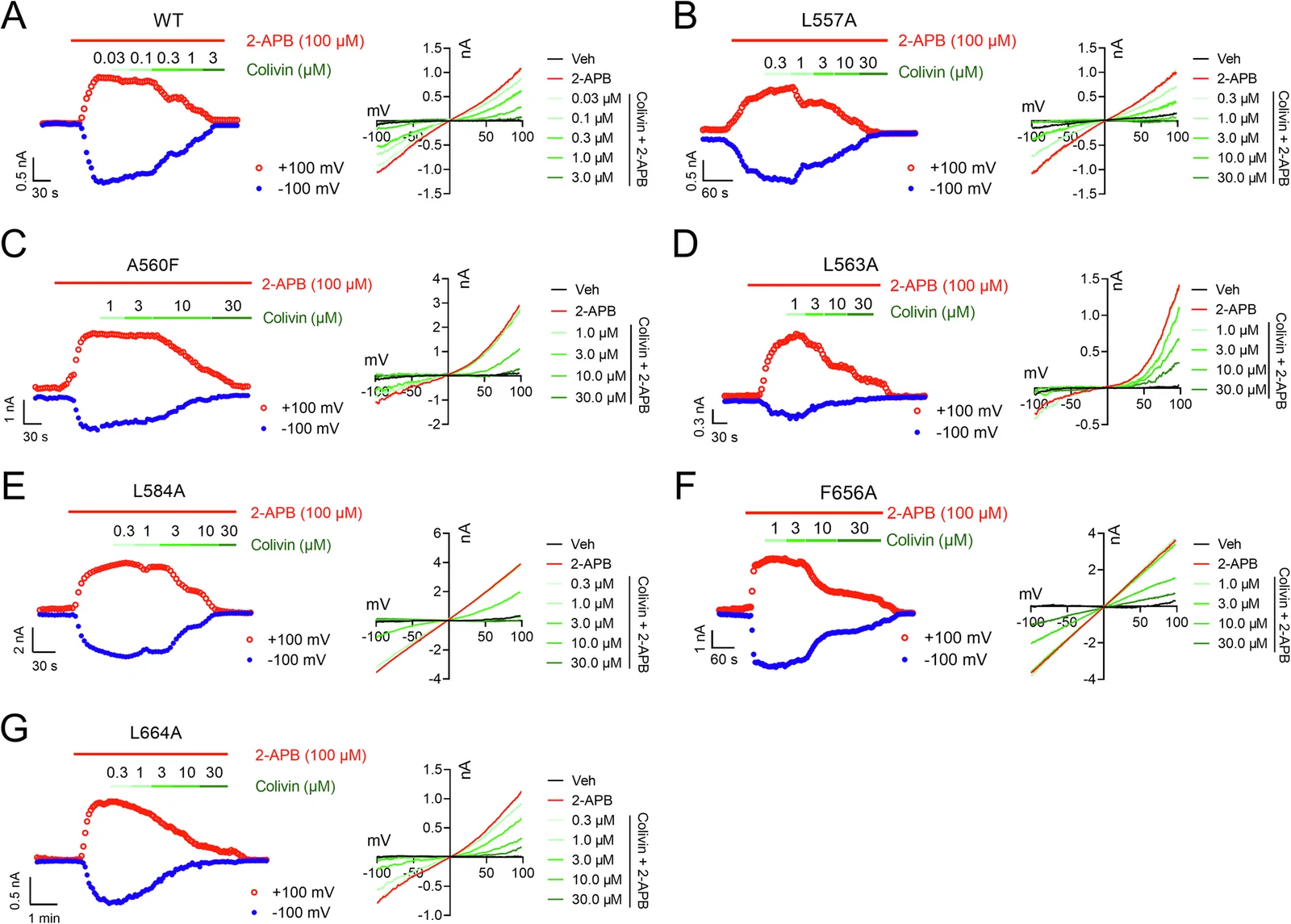
Mutations in the vanilloid site reduced the apparent affinity of Colivin on hTRPV3. Effects of different concentrations of Colivin on 2-APB (100 µM)-evoked whole-cell currents at ±100 mV in HEK-293 cells transiently expressing wild-type (WT) hTRPV3 (**A**) and L557A (**B**), A560F (**C**), L563A (**D**), L584A (**E**), F656A (**F**), and L664A (**G**) mutants of hTRPV3. Also shown are representative I–V curves of WT hTRPV3 and its mutants, derived from the different time points of drug applications. Related to Fig. 2. Source data are available online for this figure.

**Figure EV5:**
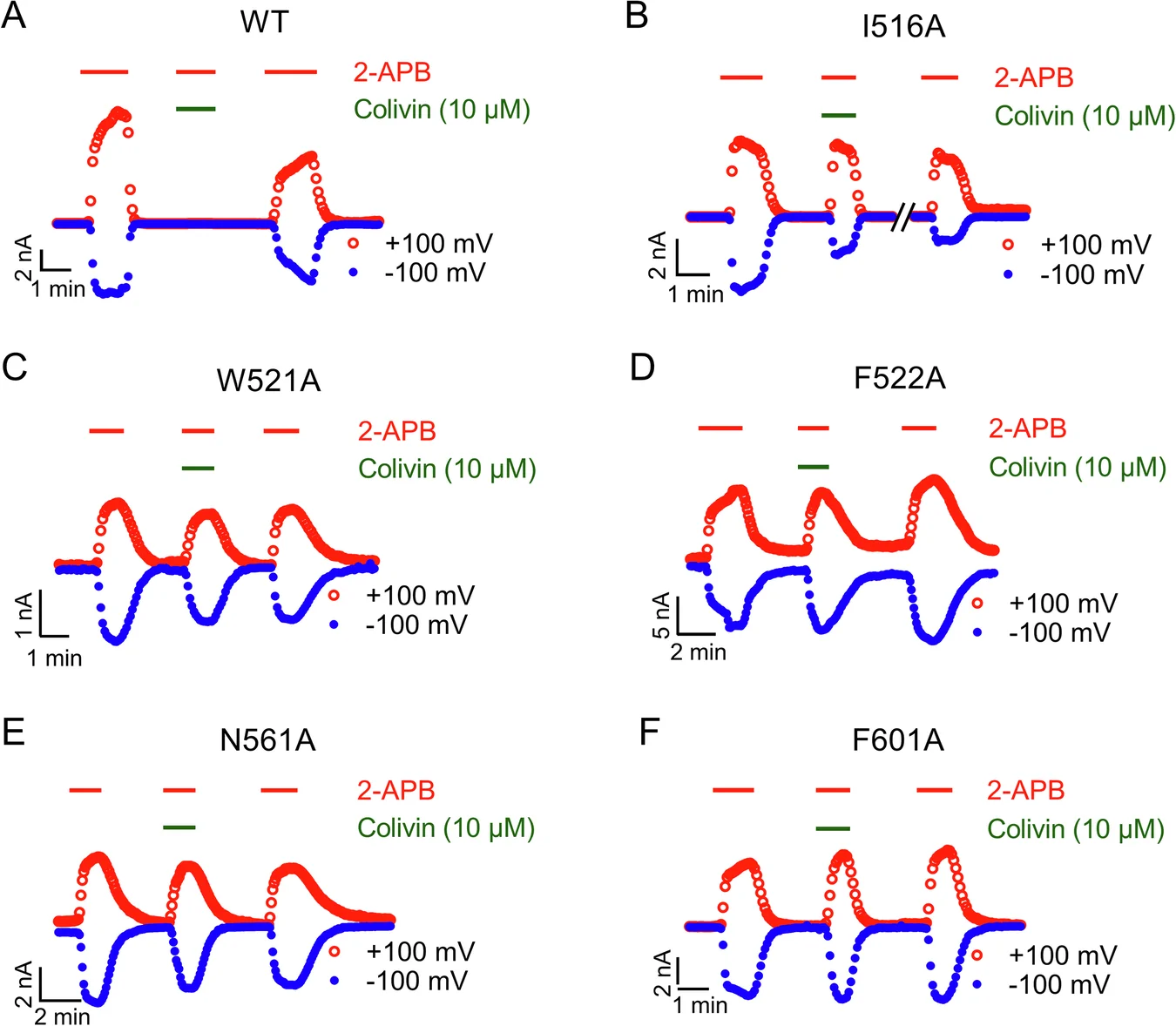
Colivin inhibition of hTRPV3 was abolished by mutations at critical residues. Representative whole-cell current traces at ±100 mV with bath applications of 2-APB (100 µM) and Colivin (10 µM) as indicated in HEK-293 cells transiently expressing wild-type (WT) hTRPV3 (**A**) and mutants: I516A (**B**), W521A (**C**), F522A (**D**), N561A (**E**), and F601A (**F**). Related to Fig. 2. Source data are available online for this figure.

Colivin binds the vanilloid-binding pocket through a combination of van der Waals forces, hydrophobic contacts and a hydrogen bond (Fig. 2C). In particular, the side chain of Asn561 likely forms a hydrogen bond with the hydroxyl group of Colivin, while hydrophobic residues including Ile516, Phe522, Leu557, Ala560, Leu563, Ile583, Leu584, Phe601, Phe656 and Leu664 stabilize its aliphatic groups *via* hydrophobic interactions. With the exception of the Gln580Ala and Ile583Ala mutants—which were insensitive to 2-APB despite successful expression in HEK-293 cells (Appendix Fig. S1), substitutions of Leu557, Leu563, Leu584, Phe656, or Leu664 with Ala, or Ala560 with Phe, increased the IC_50_ value of Colivin (0.28 μM, +100 mV recording) by more than 10-fold (Figs. 2D,E and EV4; Table 4). Furthermore, Ala substitutions of Ile516, Trp521, Phe522, Asn561, and Phe601 reduced Colivin (10 µM)-mediated inhibition to less than 30%, confirming the functional importance of these residues for Colivin binding (Figs. 2D and EV5; Table 4). Notably, both Trp521Ala and Asn561Ala mutants were completely insensitive to Colivin (10 μM), likely due to the disappearance of the π-π stacking formed by the bulky side chain of Trp521 and the pyridine moiety of Colivin, as well as the loss of the hydrogen bond between the carboxamide group of Asn561 and the hydroxyl group of Colivin. Sequence alignment demonstrated that the key amino acid residues involved in Colivin interaction are identical between hTRPV3 and mTRPV3, consistent with the similar apparent affinities of Colivin between hTRPV3 and mTRPV3. However, these critical amino acid residues substantially differ in hTRPV1, mTRPV2, hTRPV4, hTRPA1, and hTRPM8, explaining the selective inhibition of TRPV3 by Colivin (Fig. 2F).

**Table 4:** Inhibitory effects of Colivin on hTRPV3 and its mutants.

Mutation	% Inhibition (10 μM)	IC_50_ (μM)
WT	97.0 ± 0.9	0.28 ± 0.07
I516A	24.6 ± 10.5	>10
W521A	5.7 ± 2.5	>10
F522A	15.8 ± 7.1	>10
L557A	82.9 ± 6.5	2.31 ± 0.65
A560F	52.1 ± 6.2	8.61 ± 0.69
N561A	4.7 ± 2.4	>10
L563A	51.3 ± 15.3	10.72 ± 1.41
L584A	84.7 ± 4.2	3.14 ± 1.29
F601A	10.8 ± 5.1	>10
F656A	57.2 ± 9.7	7.71 ± 0.86
L664A	75.8 ± 5.7	2.71 ± 1.01

### Colivin stabilizes TRPV3 in a non-conducting conformation

**Figure 3:**
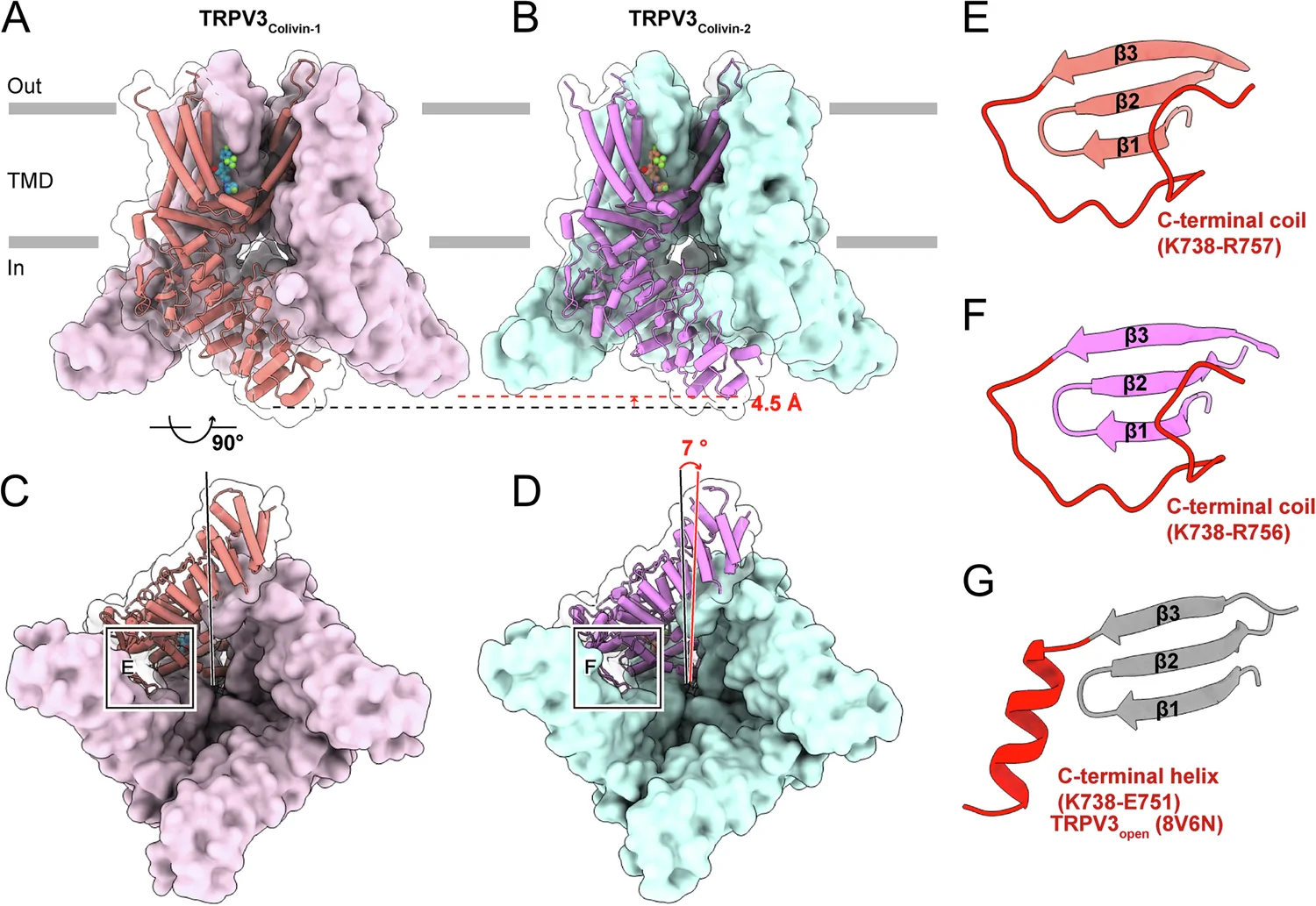
Conformational changes of TRPV3 induced by Colivin binding. (**A**, **B**) Structures of TRPV3_Colivin-1_ (**A**) and TRPV3_Colivin-2_ (**B**) viewed parallel to the membrane. Dashed lines indicate the upward movement of ARD in TRPV3_Colivin-2_ (**B**). One protomer is shown in cartoon, and the other three are shown in surface. (**C**, **D**) Structures of TRPV3_Colivin-1_ (**C**) and TRPV3_Colivin-2_ (**D**) viewed from the intracellular side. Solid lines indicate the clockwise rotation of ARD in TRPV3_Colivin-2_ (**D**). (**E**–**G**) Interfaces between adjacent protomers in TRPV3_Colivin-1_ (**E**) and TRPV3_Colivin-2_ (**F**), derived from structures in C and D, and TRPV3_open_ (8V6N) (**G**), formed by a C-terminal region, K738 – R757.

The TRPV3_Colivin-1_ and TRPV3_Colivin-2_ structures share a similar overall architecture, with a root mean square deviation (RMSD) of 0.867 Å. Both Colivin-bound structures share a similar overall architecture with the structures of TRPV3_apo_, TRPV3_closed_ (PDB ID: 8V6K), and TRPV3_inactivated_ (PDB ID: 8V6M) (Appendix Fig. S2A–F). The structural differences are mainly located in the cytosolic ankyrin repeat domains (ARDs). Compared to those in TRPV3_Colivin-1_, TRPV3_apo_, TRPV3_closed_ and TRPV3_inactivated_, the ARDs in TRPV3_Colivin-2_ shift ~4.5 Å toward the membrane and rotate ~7° clockwise around the central symmetry axis when viewed intracellularly (Fig. 3A–D; Appendix Fig. S2A–F).

Both TRPV3_Colivin-1_ and TRPV3_Colivin-2_ represent non-conducting states, with the width of the upper selectivity filter comparable to that of the apo, closed and inactivated states. Although their lower gate pore radii are slightly larger than those of TRPV3_apo_ and TRPV3_closed_, this increase remains smaller than the lower gate dilation observed in the open state (Fig. 2G–J; Appendix Fig. S2G–I). The upper selectivity filter (SF) restriction is defined by the main-chain carbonyl oxygens of Gly638, while the lower gate restriction is formed by the side chains of Ile674 on the π-helix formed by S6 in both TRPV3_Colivin-1_ and TRPV3_Colivin-2_ structures. The SF radii in TRPV3_Colivin-1_ and TRPV3_Colivin-2_ are approximately 0.8–1.0 Å, comparable to that of TRPV3_apo_, TRPV3_closed_, and TRPV3_inactivated_, and are therefore too narrow to permeate hydrated cations such as Ca^2+^ (radius 3–4.5 Å) (Fig. 2J). By contrast, the radii in the lower gate of TRPV3_Colivin-1_ and TRPV3_Colivin-2_ are ~1.3–1.6 Å, intermediate between those observed in TRPV3_apo_ and TRPV3_open_ (PDB ID: 8V6N) (Fig. 2J). The length and orientation of the TRP helix in both Colivin-bound structures remain largely unchanged compared to the apo or closed state, indicating that Colivin does not induce the TRP helix rearrangements associated with inactivation (Appendix Fig. S2J, K, L). Similar to the apo and closed states, the pore-forming S6 helices in both Colivin-bound complexes adopt a π-helical configuration, unlike the inactivated state, which instead displays a π-to-α transition (Appendix Fig. S2G–I). In the previously solved structures, the folding of the C terminal region, Lys738-Glu751, into an α helix represents a hallmark of TRPV3 in the open state (Nadezhdin et al, 2024). In TRPV3_Colivin-1_ and TRPV3_Colivin-2_, however, this region instead forms a coil that wraps around the three-stranded β sheet (Fig. 3E–G), further supporting that TRPV3 adopts a non-conducting state in the presence of Colivin.

### Colivin alleviates DSS-induced UC in mice

**Figure 4:**
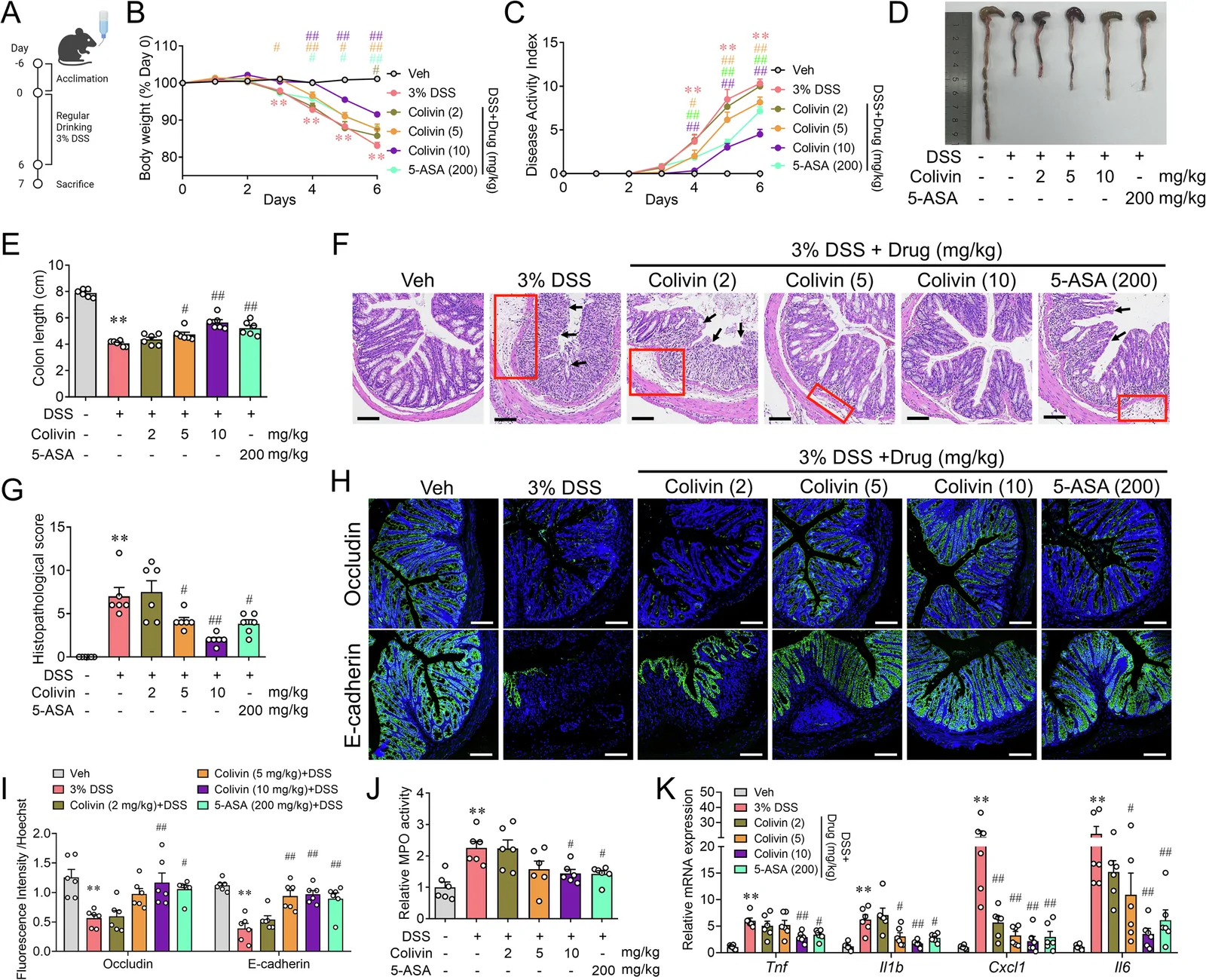
Colivin ameliorates DSS-induced ulcerative colitis in mice. (**A**) Experimental protocol for DSS-induced colitis mouse model. (**B**) Changes in body weight in WT mice treated with Veh or DSS for 6 days. When indicated, Colivin or 5-ASA was administered through oral gavage twice daily during the same 6-day period. Data represent Mean ± SEM (*N* = 6 mice). **P < 0.01, *vs*. Veh-treated mice; ^#^*P* < 0.05, ^##^*P* < 0.01, *vs*. DSS, by two-way ANOVA with Dunnett’s multiple comparisons tests. Exact *P* values for all comparisons are listed in Dataset EV1. (**C**) Disease Activity Index of the same mice treated with Veh or DSS together or not with Colivin or 5-ASA administration as in (**B**). Data represent Mean ± SEM (*N* = 6 mice). **, *P* < 0.01, *vs*. Veh-treated mice; ^#^*P* < 0.05, ^##^*P* < 0.01, *vs*. DSS, by Two-way ANOVA with Dunnett’s multiple comparisons tests. Exact *P* values for all comparisons are listed in Dataset EV1. (**D**, **E**) Representative colon images (**D**) and quantification of colon length (**E**) in Veh and DSS-treated mice, together or not with Colivin or 5-ASA administration. (**F**) Representative micrographs of colon tissues from mice in different groups stained with H&E. Disappearance of crypts and edematous lamina propria containing inflammatory cell infiltrates are indicated by black arrows and red boxes, respectively. Scale bars = 100 μm. (**G**) Quantification of histopathological scores of H&E-stained colon tissues from mice in different groups. (**H**) Representative immunofluorescent images of colon tissues stained for occludin (top panel) and E-cadherin (bottom panel) from mice exposed to 3% DSS for 6 days and treated with vehicle, different doses of Colivin, or the positive control 5-ASA (200 mg/kg). Nuclei were stained by Hoechst 33342 (blue). Scale bar = 100 μm. (**I**) Quantification of occludin and E-cadherin expression levels in different groups. (**J**) MPO activity in colon tissues from mice in different groups. (**K**) Quantification of mRNA levels of *Tnf*, *Il1b*, *Cxcl1*, *Il6* in colon tissues from mice in different groups. Data in (**E**, **G**, **I**–**K**) represent mean ± SEM (*N* = 6 mice). **P < 0.01, *vs*. Veh; ^#^*P* < 0.05, ^##^*P* < 0.01 *vs*. DSS alone, by one-way ANOVA with Dunnett’s multiple comparisons tests. Exact *P* values for all comparisons are listed in Dataset EV1. Source data are available online for this figure.

Consistent with previous demonstrations of TRPV3 expression in the gastrointestinal system, immunofluorescent staining revealed abundant TRPV3 expression within the colon tissue, the signal of which predominantly overlaps with that of CK-18, an intestinal epithelial cells (IECs) marker (Appendix Fig. S3). To test the therapeutic potential of Colivin in systemic applications, we employed the DSS-induced ulcerative colitis (UC) model, an inflammatory disease involving epithelial barrier disruption in colonocytes. After taking DSS (3%) through drinking water (Fig. 4A), wild-type (WT) mice displayed significant reductions in body weight, beginning on the third day and continuing through the sixth day (Fig. 4B). Their overall health, based on the disease activity index (DAI) determined according to Sann’s method by summing the respective scores of body weight loss, hemorrhage, and fecal consistency (Wang et al, 2021), also deteriorated throughout days 3 to 6 (Fig. 4C). Intragastric administration of Colivin (twice a day for 6 days, when DSS was included in the drinking water) resulted in dose-dependent attenuation of the DSS-induced UC, as seen by the significant mitigations in body weight loss and DAI score increase (Fig. 4B,C). DSS administration also led to colon atrophy and histopathologic changes, most notably crypt loss and lamina propria edema accompanied by inflammatory infiltration, which were suppressed by Colivin administration (Fig. 4D–G). At the dose of 10 mg/kg, Colivin alleviated the DSS-induced body weight loss by 49.6 ± 4.0% (*P* < 0.01, Fig. 4B), DAI score increase by 56.5 ± 5.4% (*P* < 0.01, Fig. 4C), colon atrophy by 41.5 ± 4.7% (*P* < 0.01, Fig. 4D,E), and histopathological score increase by 71.4 ± 3.7% (*P* < 0.01, Fig. 4F,G).

In addition, DSS exposure reduced the protein levels of occludin and E-cadherin by 55.2 ± 4.8% (*P* < 0.01) and 65.3 ± 7.8% (*P* < 0.01), respectively. Treatment with Colivin dose-dependently attenuated these DSS-induced reductions. At 10 mg/kg, Colivin significantly mitigated the DSS-induced reductions in occludin and E-cadherin expression by 86.4 ± 23.5% (*P* < 0.01) and 79.3 ± 8.6% (*P* < 0.01), respectively (Fig. 4H,I). Consistent with its robust inhibition of inflammation in the lamina propria, Colivin also dose-dependently suppressed DSS-induced increases in MPO activity and expression of pro-inflammatory factors (Fig. 4J,K). At 10 mg/kg, Colivin significantly dampened the DSS-induced increase in MPO activity by 64.8 ± 0.26% (*P* < 0.05) and the elevations of mRNA expression levels of *Tnf*, *Il1b*, *Cxcl1*, and *Il6* by 62.0 ± 6.6% (*P* < 0.05), 85.8 ± 3.9% (*P* < 0.05), 93.9 ± 3.9% (*P* < 0.01), and 88.3 ± 4.1% (*P* < 0.01), respectively (Fig. 4J,K). Notably, the mitigation of DSS-induced symptoms by 10 mg/kg of Colivin was at least as good as, if not better than 5-aminosalicylic acid (5-ASA, 200 mg/kg), a clinically approved drug for UC treatment (Fig. 4), demonstrating the therapeutic efficacy of Colivin on UC.

**Figure 5:**
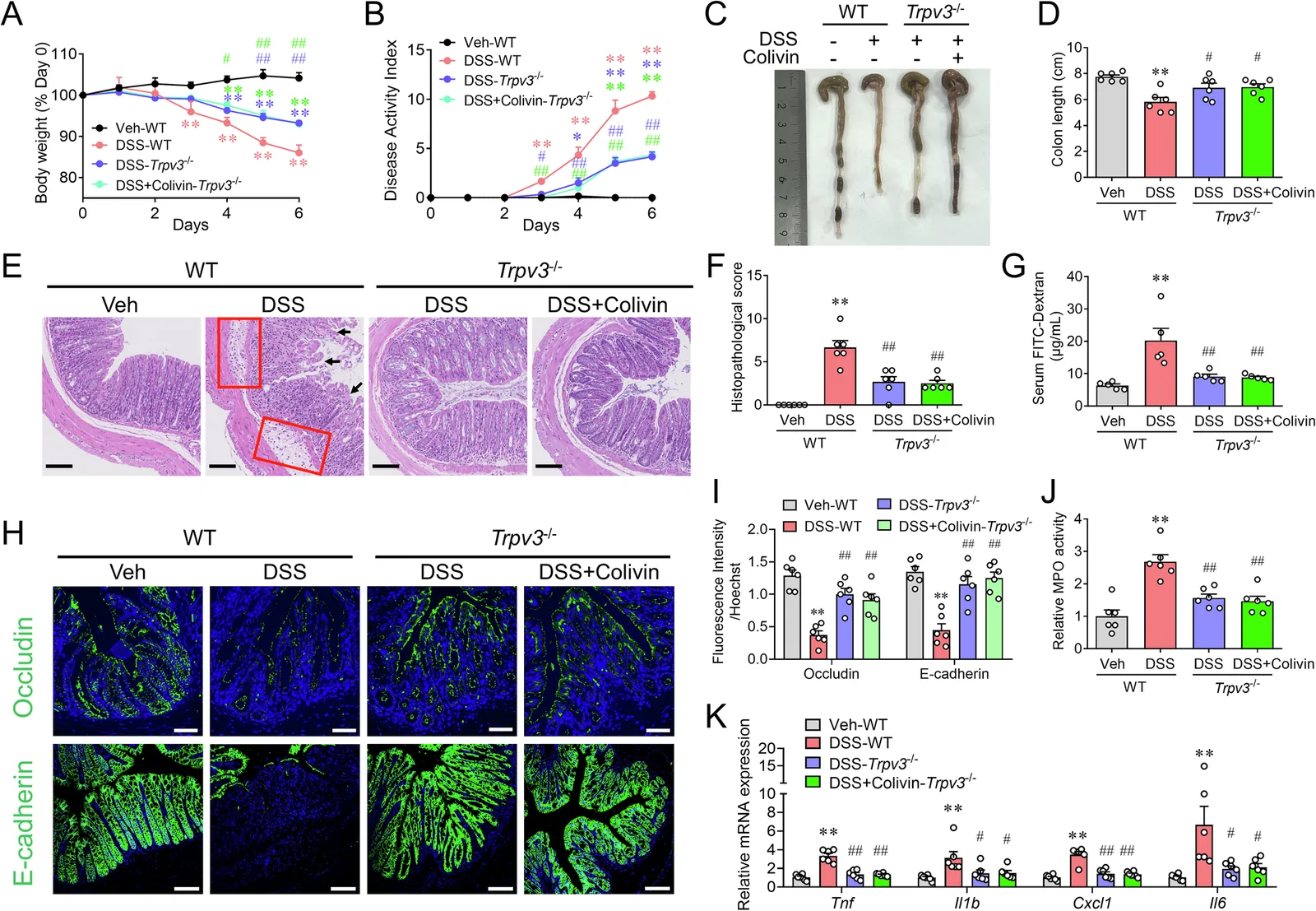
Engagement of TRPV3 by Colivin ameliorated ulcerative colitis. (**A**, **B**) Changes in body weight (**A**) and disease activity index (**B**) of WT mice treated with vehicle (Veh) or DSS and *Trpv3*^−/−^ mice treated with DSS or DSS plus Colivin (10 mg/kg, i.g. twice daily) for 6 days. Data represent mean ± SEM (*N* = 6 mice). **P < 0.01, *vs*. Veh-treated WT mice; ^#^*P* < 0.05, ^##^*P* < 0.01, *vs*. DSS-WT, by two-way ANOVA with Tukey’s multiple comparisons tests. Exact *P* values for all comparisons are listed in Dataset EV1. (**C**, **D**) Representative colon images (**C**) and quantification of colon length (**D**) in WT mice treated with Veh or DSS and *Trpv3*^−/−^ mice treated with DSS or DSS plus Colivin. (**E**) Representative micrographs of colon tissues from WT and *Trpv3*^−/−^ mice stained with H&E. Disappearance of crypts and edematous lamina propria containing inflammatory cell infiltrates are indicated by black arrows and red boxes, respectively. Scale bars = 100 μm. (**F**) Quantification of histopathological scores of H&E-stained colon tissues from WT and *Trpv3*^−/−^ mice derived from (**E**). (**G**) The serum FITC-dextran levels in WT and *Trpv3*^−/−^ mice treated with Veh or Colivin. (**H**) Representative immunofluorescent images of colon tissues stained for occludin (top panel) and E-cadherin (bottom panel) from WT and *Trpv3*^−/−^ mice exposed to DSS for 6 days and treated with vehicle or Colivin (10 mg/kg). Nuclei were stained by Hoechst 33342 (blue). Scale bar = 50 μm. (**I**) Quantification of occludin and E-cadherin expression in different groups. (**J**) MPO activity in colon tissues from WT and *Trpv3*^−/−^ mice with different treatments. (**K**) Quantification of mRNA levels of *Tnf*, *Il1b*, *Cxcl1*, *Il6* in colon tissues from WT and *Trpv3*^−/−^ mice with different treatments. Data in (**D**, **F**, **G**, **I**–**K**) represent mean ± SEM (*N* = 6 mice). **P < 0.01, *vs*. Veh-treated WT mice; ^#^*P* < 0.05, ^##^*P* < 0.01, *vs*. DSS-treated WT mice, by one-way ANOVA with Tukey’s multiple comparisons tests. Exact *P* values for all comparisons are listed in Dataset EV1. Source data are available online for this figure.

To determine whether TRPV3 is the main target for the therapeutic effect of Colivin, we administered Colivin to *Trpv3*-deficient (*Trpv3*^*−/−*^) mice with DSS-induced colitis. Ablation of *Trpv3* alone significantly attenuated the DSS-induced reductions in body weight, increases in DAI scores, colon atrophy (Fig. 5A–D), confirming the role of TRPV3 in UC pathogenesis. However, administration of Colivin (10 mg/kg) to *Trpv3*^−/−^ mice provided no additional improvement on DSS-induced body weight reduction, DAI increase, and colon atrophy (Fig. 5A–D). Histological examination of H&E-stained colon sections showed that DSS treatment in WT mice disrupted crypt architecture, induced edema in the lamina propria, and triggered substantial inflammatory cell infiltration. These pathological alterations were markedly less severe in *Trpv3*^−/−^ mice, irrespective of Colivin treatment (Fig. 5E). Quantitatively, deletion of *Trpv3* significantly reduced the DSS-induced pathological score from 6.67 ± 0.80 to 2.67 ± 0.61 (*P* < 0.01, Fig. 5F).

Furthermore, DSS treatment significantly increased serum FITC-dextran levels from 6.32 ± 0.49 to 20.24 ± 3.78 µg/mL (*P* < 0.01) in WT mice 4 h following oral administration of FITC-dextran (4 kDa, 400 mg/kg) (Fig. 5G), consistent with epithelial barrier disruption associated with UC. Deletion of *Trpv3* significantly reduced this DSS-induced increase by 79.8 ± 5.2% (*P* < 0.01) and also attenuated DSS-induced reductions in occludin and E-cadherin protein levels by 68.6 ± 9.8% (*P* < 0.01) and 78.8 ± 13.2% (*P* < 0.01), respectively (Fig. 5G–I). However, Colivin treatment had no further effect on pathological score, serum FITC-dextran, occludin, or E-cadherin levels in DSS-treated *Trpv3*^*−/−*^ mice (Fig. 5F–I). Consistent with its robust inhibition of inflammation in the lamina propria, deletion of *Trpv3* also robustly suppressed DSS-induced increases in MPO activity by 65.6 ± 7.2% (*P* < 0.01) and expression levels of pro-inflammatory factors, *Tnf*, *Il1b*, *Cxcl1*, and *Il6* by 85.2 ± 8.3% (*P* < 0.01), 78.2 ± 16.3% (*P* < 0.05), 83.2 ± 8.2% (*P* < 0.01) and 83.2 ± 5.4% (*P* < 0.05), respectively (Fig. 5J,K). However, the application of Colivin failed to further suppress the MPO activity and mRNA expression levels of these inflammation mediators in the *Trpv3*^−/−^ mice (Fig. 5J,K). These data suggest that Colivin attenuation of DSS-induced intestinal barrier disruption and inflammation is entirely dependent on TRPV3.

## Discussion

TRP channels, especially the TRPV subfamily members, are implicated in many (patho)physiological functions and are, therefore, actively pursued as drug targets for therapeutic development (Koivisto et al, 2022). The discovery that gain-of-function mutations in TRPV3 underlie Olmsted syndrome in humans, as well as spontaneous hair loss and dermatitis in rodents, has prompted extensive research into the role of TRPV3 in the skin system (Lin et al, 2012; Yoshioka et al, 2009). It has been well-established that TRPV3 serves as a key molecular regulator of skin inflammation and skin barrier formation, in addition to contributing to itch and pain sensations in the sensory neurons (Cheng et al, 2010; Szöllősi et al, 2018; Wang et al, 2022). However, much less is known about TRPV3 outside the skin keratinocytes and sensory neurons. Nonetheless, TRPV3 expression has been found in the oral and nasal epithelium, where the channel plays a role in chemesthesis (Xu et al, 2006), and in the intestinal epithelium, where it was reported to regulate the absorption of ammonium (Liebe et al, 2021; Manneck et al, 2021). In the current study, we show that both genetic deletion and pharmacological inhibition can alleviate intestinal inflammation and pathogenic changes in the DSS-induced mouse UC model, further strengthening the role of TRPV3 in the gastrointestinal system, including the development of UC.

Our findings suggest that inhibiting TRPV3 may be a viable strategy for treating UC. Although several TRPV3 inhibitors have been reported (Black et al, 2016; Gomtsyan et al, 2016; Lv et al, 2021; Qi et al, 2022; Sun et al, 2018; Wang et al, 2022), they often lack the high affinity or selectivity for TRPV3, or they display poor oral bioavailability. The latter issue is not a problem for topical applications if the treatment areas are on exposed surfaces, such as the skin and oral cavity. However, for UC treatment, systemic applications are preferred, which requires the drug to be metabolically stable. For this reason, we synthesized a series of 2-pyridinecarboxamide derivatives based on KM-001, a TRPV3 inhibitor undergoing Phase I clinical trials for the treatment of pruritus (Barak et al, 2023). We aimed to preserve the high affinity and selectivity for TRPV3 but improve the metabolic stability of the compound. Among these derivatives, Colivin was found to display similar high potency and selectivity in inhibiting TRPV3 as the original compound, but markedly improved bioavailability after oral administration. In DSS-induced mouse colitis model, oral administration of Colivin dose-dependently alleviated symptoms, histopathological changes and inflammation in colon tissues. We also demonstrate that deletion of *Trpv3* drastically suppressed DSS-induced colitis. More importantly, Colivin had no additional improvement against these alterations in *Trpv3*^*−/−*^ mice. This provides a novel pharmacological probe to unveil the in vivo pathophysiological role of TRPV3 in a broader range of body systems beyond the skin, establishing this channel as a previously undescribed critical molecular regulator to control intestinal inflammation and a promising target for developing treatment for UC.

In the current study, we demonstrate that TRPV3 is predominantly expressed in CK18^+^ IECs and that inhibiting TRPV3, either by Colivin or gene ablation, alleviates DSS-induced IEC barrier disruption. It is thus tempting to conclude that IEC-expressed TRPV3 serves as a major contributor to UC by disrupting the epithelial barrier. However, TRPV3 has also been reported to be expressed in other cell types, such as sensory neurons and immune cells (Çakır et al, 2021; McGaraughty et al, 2017; Niu et al, 2025), both of which have been shown to regulate UC progression (Engel et al, 2012; Ungaro et al, 2017). Therefore, cell-type-specific *Trpv3* knockout mice should be tested in order to gain a comprehensive understanding of how TRPV3 in different intestinal cell types contributes to UC progression. Additional studies are also needed to unveil the molecular and signaling mechanisms involved. Moreover, as a polymodal sensor, TRPV3 responds not only to exogenous natural and synthetic compounds such as camphor, carvacrol, and 2-APB (Su et al, 2023), but also to endogenous environmental factors, including arachidonic acid derivatives that are elevated in the inflamed colonic mucosa of UC patients (Hu et al, 2006; Nishida et al, 1987) and isopentenyl pyrophosphate, a metabolic alarmin (Bang et al, 2010; Ji et al, 2026). TRPV3 can also be potentiated by phospholipase C (PLC)-dependent release from PIP_2_ inhibition, as well as by ERK-dependent phosphorylation of Thr264 in the N-terminal ankyrin repeat domain (Doerner et al, 2011; Vyklicka et al, 2017), two signaling pathways that are often activated by inflammatory mediators such as IL-1β and TNF-α (Amin et al, 2003; Eliopoulos et al, 2003). Future studies are needed to determine the endogenous signals responsible for TRPV3 activation under inflammatory conditions such as UC.

To unveil the molecular details of Colivin action on TRPV3, we employed single-particle cryo-EM analysis. This method has been successfully applied on the mechanisms of TRPV3 modulation by small molecules, which revealed several distinct binding pockets. For example, osthole binds to two distinct pockets, which are formed by *i*) the intracellular half of the S1–S4 bundle and the C-terminal TRP helix, and *ii*) the nexus of the linker domain, pre-S1, and TRP helices, to convert the channel to a state with a widely open selectivity filter and a closed intracellular gate (Neuberger et al, 2021). Dyclonine binds to a site within the portals formed by the S5 and S6 helices (Neuberger et al, 2022). Here, we show that Colivin binds to another pocket formed by the S3, S4, and S4-S5 linker of one subunit and the S5 and S6 helices of the neighboring subunit. The upper and lower gates of the channel remain closed, consistent with the role of this drug as a TRPV3 inhibitor. This binding pocket, which overlaps with the vanilloid pocket of TRPV1, has previously been shown to be bound by another TRPV3 antagonist, Trpvicin; however, Trpvicin only interacts with the upper part of the pocket without engaging the S4-S5 linker (Fan et al, 2022), indicating different binding modes between the two inhibitors at the same binding pocket. Interestingly, although the vanilloid pocket was originally identified as the binding site of TRPV1 agonists, such as capsaicin and resiniferatoxin (Kwon et al, 2021), later studies demonstrated that it also accommodates many TRPV1 antagonists (Fan et al, 2024). Likewise, the vanilloid pocket of TRPV3 has also been shown to be bound by TRPV3 agonists, including the natural cannabinoid tetrahydrocannabivarin (THCV) and acyclic monoterpenes (Li et al, 2025; Nadezhdin et al, 2024). Similar to the case of TRPV1, it is likely that binding by different agonists/antagonists may engage interactions with distinct sets of amino acid residues within the vanilloid pocket, resulting in unique conformational changes that either promote or inhibit channel opening.

We observed two distinct protein conformations in the Colivin-bound structures: TRPV3_Colivin-1_ and TRPV3_Colivin-2_. In both conformations, Colivin induces an outward movement of the S4-S5 linker and S6 helix compared to TRPV3_apo_. However, the structures differ at the cytoplasmic side. While the ARD in TRPV3_Colivin-1_ maintains a similar structure as TRPV3_apo_, in TRPV3_Colivin-2_, it shifts ~4.5 Å toward the membrane. This upward movement of the intracellular skirt was typically observed in open conformations induced by THCV, 2-APB, and temperature, representing the initial step in TRPV3 channel opening. Such a movement has been reported to facilitate the expulsion of the lipids, which are constitutively associated with the channel under resting conditions and keep the channel in the closed state, from the vanilloid pocket (Nadezhdin et al, 2024; Nadezhdin et al, 2021; Singh et al, 2018). Similarly, structural work has revealed that phosphatidylinositol lipids stabilize the closed state by occupying the vanilloid pocket of TRPV1 (Arnold et al, 2024; Gao et al, 2016; Zhang et al, 2021). At the same time, agonist binding induces a transition of a C-terminal coiled region that wraps around the three-stranded β sheet connecting two adjacent TRPV3 protomers into an α helix that is required for the open conformation of TRPV3 (Nadezhdin et al, 2024; Nadezhdin et al, 2021; Singh et al, 2018). However, in both TRPV3_Colivin-1_ and TRPV3_Colivin-2_, this C-terminal region maintains a coiled conformation wrapping around the three-stranded β sheet. Thus, despite the upward movement of the intracellular skirt, TRPV3_Colivin-2_ does not completely recapitulate the conformational transition typically seen for the open TRPV3 channel, likely representing an intermediate state.

Upon binding, Colivin wedges into the vanilloid site of TRPV3 and induces extensive conformational rearrangements. These include an inward movement of the S4-S5 linker toward the channel pore and a displacement of the S6 helix to accommodate Colivin, accompanied by a rotation of the intracellular skirt toward the membrane. The extensive interactions of Colivin with the S6 helix and S4-S5 linker restrict the outward movement of the pore-lining S6 helices required for channel opening, thereby locking TRPV3 in a non-conducting conformation. These structural insights elucidate the molecular basis of TRPV3 inhibition by Colivin.

Collectively, these data highlight inhibiting TRPV3 as a promising therapeutic strategy for UC and provide the structural foundation for the rational design of potent, selective TRPV3 inhibitors targeting UC and other TRPV3-related disorders.

## Methods

**Table:** Reagents and tools table

Reagent/resource	Reference or source	Identifier or catalog number
**Experimental models**		
C57BL/6J mice	The Experimental Animal Center of Yangzhou University	MGI: 3028467
*Trpv3*^−/−^ mice	The Experimental Animal Center of Nanjing Medical University	
**Recombinant DNA**		
pEX-1	GenePharm Biotechnology Co., Ltd.	Cat#: C05001
**Antibodies**		
Anti-occludin	Cell Signaling Technology	Cat# 91131
Anti-E-cadherin	Cell Signaling Technology	Cat# 3195
Anti-rabbit Alexa Fluor® 488	Cell Signaling Technology	Cat# 4412
Anti-TRPV3	Novus Biologicals	Cat# NBP2-12918
Anti-cytokeratin 18	Bioworld Technology	Cat# BZ17054
**Oligonucleotides and other sequence-based reagents**		
Primers	Beijing Tsingke Biotechnology Co., Ltd.	Appendix Tables 1 and 2
**Chemicals, enzymes and other reagents**		
Chemicals and reagents	This study	Detailed in Reagents section
**Software**		
UCSF ChimeraX	https://www.cgl.ucsf.edu/chimerax/ Goddard et al, 2018	
Coot	www2.mrc-lmb.cam.ac.uk/personal/pemsley/coot/ Emsley and Cowtan, 2004	
Phenix	https://phenix-online.org/download Afonine et al, 2018	
MolProbity	https://phenix-online.org/documentation/reference/molprobity_tool.html Chen et al, 2010	
GraphPad Prism version 8.01	https://www.graphpad.com/	
**Other**		

### Reagents

Dulbecco’s Modified Eagle Medium (DMEM), fetal bovine serum (FBS), penicillin/streptomycin (P/S), and trypsin were purchased from Life Technologies (Grand Island, NY, USA). SMM 293-TII Expression Medium (Cat# M293TII-N) was from SinoBiological (Beijing, China). Dextran sulfate sodium (DSS, Cat# 160110) was obtained from MP Biomedicals (Santa Ana, CA, USA). 2-APB (Cat# HY-W009724) was obtained from MedChemExpress (Monmouth Junction, NJ, USA). The myeloperoxidase (MPO) activity assay kit (Cat# A114-1-1) was supplied by Nanjing Jiancheng Bioengineering Institute (Nanjing, Jiangsu, China). FITC-dextran (MW4,000, Cat#ST2930) was from Beyotime Biotechnology. The following antibodies were sourced from Cell Signaling Technology (Danvers, MA, USA): anti-occludin (Cat# 91131), anti-E-cadherin (Cat# 3195) and anti-rabbit Alexa Fluor^®^ 488 (Cat# 4412). Anti-TRPV3 antibody (Cat# NBP2-12918) and anti-cytokeratin 18 (CK18) antibody (Cat# BZ17054) were from Novus Biologicals (Centennial, CO, USA) and Bioworld Technology (Nanjing, Jiangsu, China), respectively. Glyco-diosgenin (GDN, Cat# GDN101), n-dodecyl-β-D-maltopyranoside (DDM, Cat# D310), and cholesteryl hemisuccinate tris salt (CHS, Cat# CH210) were obtained from Anatrace (Maumee, OH, USA). Desthiobiotin (Cat# 2-1000-002) was from IBA Lifesciences (Göttingen, Germany). Sodium butyrate (Cat# A45269) was purchased from Beijing Vokai Biotechnology Co., LTD (Beijing, China). Aprotinin (Cat# A100429), leupeptin (Cat# A600580), pepstatin A (Cat# A610583), and phenylmethyl sulfonyl fluoride (PMSF, Cat# A100754) were supplied from Sangon Biotech (Shanghai, China). Tribromoethanol and all inorganic salts were supplied from Sigma-Aldrich (St. Louis, MO, USA) unless otherwise mentioned. The pEX-1 vector containing cDNA of human (h)*TRPV3* (NM_001258205.2) was constructed by GenePharm Biotechnology Co., Ltd. (Shanghai, China). Colivin was synthesized by Prof. Jianhua Shen at the Shanghai Institute of Materia Medica, Chinese Academy of Sciences, and its purity (> 99%) was confirmed by High-Performance Liquid Chromatography (Appendix Figs. S4–7).

### Cell culture

HEK-293F cells were cultured in SMM 293-TII Expression Medium (SinoBiological, Beijing, China) supplemented with FBS (1% v/v). HEK-293T cells were maintained in DMEM supplemented with 10% FBS, 100 U/mL of P/S at 37 °C with 5% CO_2_. HEK-293T cells were transiently transfected with h*TRPV3* plasmid using Lipofectamine 2000 reagent. Experiments were conducted 12–24 h after transfection. HEK-293T cells stably expressing h*TRPV1*, mouse (m)*Trpv2*, m*Trpv3*, h*TRPV4*, h*TRPA1*, and h*TRPM8* were the same cell lines used in our previous studies (Wang et al, 2022).

### Intracellular Ca^2+^ measurement

Intracellular Ca^2+^ level measurement was performed to determine the Colivin effect on TRP channels as described previously (Ding et al, 2024). In brief, cells were incubated with Fluo-4/AM (4 μM) in Locke’s buffer (in mM: 8.6 HEPES, 5.6 KCl, 154 NaCl, 5.6 glucose, 1.0 MgCl_2_, 2.3 CaCl_2_, and 0.0001 glycine, pH 7.4) containing 0.5% BSA. After washing with Locke’s buffer for five times, cells were transferred to the cell plate position of a fluorescent imaging plate reader (FLIPR^TETRA®^; Molecular Devices, Sunnyvale, CA, USA). Excitation wavelength was set at 470–495 nm, and emission was recorded at 515–575 nm. To analyze the concentration-response relationships on TRPV3 and thermosensitive TRP channels, cells were treated with different concentrations of Colivin for 5 min, followed by respective agonist: capsaicin (1 μM) for hTRPV1, 2-APB (100 μM) for mTRPV2, 2-APB (50 μM) for hTRPV3, GSK1016790A (10 nM) for hTRPV4, allyl isothiocyanate (AITC, 30 μM) for hTRPA1, and menthol (300 μM) for hTRPM8. All the compounds were dissolved in Locke’s buffer containing 0.1% DMSO. The area under the fluorescence intensity-time curve (AUC) calculated from the 300-s duration after agonist addition in each well was used to determine the effects of the test compounds on TRP channels.

### Electrophysiology

As described previously (Wang et al, 2022), whole-cell patch-clamp recordings were performed at room temperature using an EPC-10 amplifier (HEKA Elektronik, Lambert/Pfalz, Germany). Electrodes were pulled from borosilicate glass using P-1000 Micropipette Puller (Sutter Instrument, Novato, CA, USA) to 3–5 MΩ when filled with a pipette solution containing (in mM): 140 CsCl, 1 MgCl_2_, 5 EGTA, 10 HEPES, pH 7.20. The bath solution contained (in mM): 140 NaCl, 5 KCl, 1 MgCl_2_, 10 glucose, and 10 HEPES, pH 7.40. Membrane potential was held at 0 mV, and currents were elicited by a protocol of a voltage ramp from −100 to +100 mV every 650 ms. Compounds were locally delivered to the recorded cell *via* a pressure-driven multichannel perfusion system (ALA Scientific Instruments, Farmingdale, NY, USA).

### Site-directed mutagenesis

The primers designed to complement the target DNA region and incorporate the desired mutation were synthesized by Beijing Tsingke Biotechnology Co., Ltd. (Beijing, China) and listed in Appendix Table S1. All mutations were generated using the KOD plus mutagenesis kit (Cat# SMK-101, Toyobo, Osaka, Japan) and verified by DNA sequencing. For recombinant expression validation, successful expression was confirmed by a C-terminally fused green fluorescent protein (GFP) tag on TRPV3.

### Protein expression and purification

The coding sequence for full-length h*TRPV3* (NM_001258205.2, UniProt ID: Q8NET8) was subcloned into a pEG BacMam vector containing a 3 C protease cleavage site and a YFP-twin-Strep tag at the C-terminus. The TRPV3 protein was expressed in HEK-293F cells. When the cell density reached approximately 3.0 × 10^6^ cells/mL, one liter of cells was transiently transfected with 1 mg of the plasmids, which were pre-mixed with polyethylenimine (Yeasen Biotechnology, Shanghai, China) at a 1:4 (w/w) ratio. After 10 h of culture at 37 °C with 8% CO_2_, 10 mM sodium butyrate was added to boost protein expression. Cells were cultured for an additional 48 h at 30 °C before being harvested.

All purification procedures were performed at 4 °C unless indicated otherwise. Cells were solubilized in the buffer A containing 20 mM Tris (pH 8.0), 150 mM NaCl, 1% (w/v) DDM, 0.2% (w/v) CHS, 0.8 µM aprotinin, 2 µg/ml leupeptin, 2 mM pepstatin A, and 1 mM PMSF for 1.5 h under gentle agitation, followed by centrifugation at 186,000 × *g* for 1 h at 4 °C. The supernatant was passed through a 0.45-µm polystyrene membrane and incubated with Strep-Tactin resin for 1 h. After washing with buffer B consisting of 20 mM Tris (pH 8.0), 150 mM NaCl, 0.01% (w/v) GDN, proteins were eluted by buffer B supplemented with 5 mM desthiobiotin from the resin. The elution was digested by 3C protease for 1 h, concentrated, and loaded onto a Superose 6 10/300 GL column (GE Healthcare) in the presence of buffer B. The protein peak fractions were collected and further analyzed by SDS-PAGE.

### Cryo-EM sample preparation and data collection

The purified TRPV3 protein was concentrated to ~8 mg/mL for cryo-EM sample preparation. For the TRPV3_Colivin_ structure, Colivin was added to the sample at a final concentration of 1 mM and incubated for 30 min at 4 °C. A droplet of 4 µL of the sample was applied onto a glow-discharged Quantifoil R2/1 200-mesh gold holey carbon grid. The grid was then blotted for 3 s at 8 °C under 95% humidity using a Vitrobot Mark IV (Thermo Fisher Scientific, Waltham, MA, USA), and flash-frozen with liquid ethane cooled by liquid nitrogen. Cryo-EM grids were imaged on a Titan Krios microscope (Thermo Fisher Scientific) operating at 300 kV, equipped with a K3 direct electron detector (Gatan) and an energy filter for data collection. Movies were captured using EPU software at a calibrated magnification of 81,000× in the super-resolution mode, resulting in 0.5275 Å per pixel. The defocus range was set between −1.0 and −2.0 μm. Each micrograph was recorded with a total dose of 50 e^-^/Å^2^ at a dose rate of ~25 e^-^/pixel/s.

### Cryo-EM data processing

All datasets were processed in cryoSPARC v4.5.3 (Punjani et al, 2017). In total, 1002 and 1200 cryo-EM movies were collected for TRPV3_apo_ and TRPV3_Colivin_, respectively. Gain-normalized image stacks were subjected to beam-induced motion correction using Patch Motion Correction, followed by contrast transfer function (CTF) estimation using Patch CTF. Micrographs with a CTF fitting resolution worse than 6 Å were removed by manual curation of exposures to exclude images of poor quality. Particles picked by the blob picker and template picker were combined, and duplicate entries (within ~30 Å) were removed. The particles were used for an ab initio 3D reconstruction to generate initial models and garbage collectors. Further refinement was achieved by heterogeneous refinement using the volumes generated in the ab initio reconstruction as templates.

For the TRPV3_apo_ dataset, two rounds of heterogeneous refinement were performed with C1 symmetry. The subsequent refinements were all carried out with C4 symmetry imposed. The particles corresponding to the best class were refined with homogeneous refinement and non-uniform refinement, giving rise to a 3.24 Å resolution map according to the gold-standard Fourier shell correlation (GSFSC) criterion. Further local refinement was performed to yield a 3.16 Å-resolution map using the gold-standard FSC = 0.143.

For the TRPV3_Colivin_ dataset, initial heterogeneous refinement with C1 symmetry generated two well-defined classes: class 1 (212,593 particles) and class 3 (180,105 particles), with distinct structural features in the ankyrin repeat domain (ARD). Notably, subsequent processes were all carried out with C4 symmetry imposed. To reduce the heterogeneity, we carried out another round of heterogeneous refinement on class 1 and class 3, respectively. For class 1, the particles were refined with homogeneous refinement and non-uniform refinement, followed by a further 3D classification, yielding four subclasses. Among them, three subclasses showed the same conformation as the parental class 1 and were combined and subjected to nonuniform refinement and local refinement, resulting in a 3.29 Å-resolution map for TRPV3_Colivin-1_ (151,361 particles). The fourth subclass displayed the same conformation as class 3 and was named class 3a. For class 3, the best class derived from heterogeneous refinement was combined with class 3a and then subjected to nonuniform refinement and local refinement, resulting in a 3.31 Å-resolution map for TRPV3_Colivin-2_ (217,509 particles). The data processing pipelines are shown in Figs. EV2 and EV3.

### Model building

The AlphaFold-predicted model of TRPV3 was roughly fitted into the cryo-EM density map of TRPV3_apo_ by UCSF ChimeraX (Goddard et al, 2018). The fitted model was manually inspected and corrected in Coot (Emsley and Cowtan, 2004) and subsequently refined in real space using Phenix (Afonine et al, 2018). The resulting TRPV3_apo_ model was further used as a template for generating and refining models for TRPV3_Colivin__-__1_ and TRPV3_Colivin__-__2_. Geometry constraints for Colivin were produced by elBow tool in Phenix (Afonine et al, 2018). The model validation was performed using MolProbity (Chen et al, 2010) and Phenix (Afonine et al, 2018), with model refinement and validation statistics summarized in Table 3. All figures were prepared using UCSF ChimeraX (Goddard et al, 2018).

### Animals and ethics statement

The animal care and usage were conducted following the guidelines established by the National Institutes of Health (NIH), as detailed in NIH Publication No. 8023 (revised in 1978). All experimental procedures involving animals adhered to ethical standards for animal research and received approval from the Ethics Committee at China Pharmaceutical University (#SYXK, 2023-0019). Mice were housed in a vivarium with a controlled temperature of 23 ± 2 °C, under a 12-h light and dark cycle, and had unrestricted access to food and water. Efforts were made to minimize animal suffering and reduce the number of experimental animals. C57BL/6J mice (aged 7–8 weeks, 22–24 g) were sourced from Yangzhou University (Yangzhou, Jiangsu, China). The *Trpv3*^−/−^ mouse line (C57BL/6J background) was the same one as previously described (Wang et al, 2022). Investigators were not blinded to group allocation during data collection; however, outcome assessment was performed by an individual who was blinded to the treatment groups.

### DSS-induced ulcerative colitis mouse model

As described previously (Tang et al, 2024), mice were acclimatized for one week and then randomly assigned to different experimental groups. To induce UC, mice were challenged with DSS (3% in drinking water) for 6 consecutive days. Disease activity index (DAI) was scored daily according to Sann’s method by summing the respective scores for body weight loss, hemorrhage and fecal consistency (Wang et al, 2021). On the seventh day, mice were sacrificed, colon length was measured, and the distal colon tissues were carefully harvested and immediately frozen for further analysis.

To evaluate the therapeutic effects of Colivin on UC, mice received twice-daily oral gavage of the TRPV3 inhibitor, Colivin, at doses of 2, 5, or 10 mg/kg each time for 6 consecutive days, together with the DSS challenge. The positive control was 5-aminosalicylic acid (5-ASA; 200 mg/kg; twice-daily). To evaluate the role of TRPV3 on UC, we also treated time-matched *Trpv3*^*−/−*^ littermates (both male and female) with DSS. To examine the engagement of TRPV3 on Colivin therapeutic effect on UC, we administered Colivin (10 mg/kg, twice-daily) to DSS-treated *Trpv3*^*−/−*^ mice. All compounds were dissolved in the vehicle solution (Veh: 2% DMSO + 2% Tween-80 + 0.5% CMC-Na). Mice in Veh and DSS groups received Veh solution following the same dosing regimen.

### Histology and immunostaining

Colon samples were fixed using 4% paraformaldehyde, embedded in paraffin, and then sectioned to a thickness of 5 µm, after which they were stained with hematoxylin and eosin. Images were captured with the NanoZoomer 2.0 RS slide scanner (Hamamatsu Photonics, Japan). Histological scores were evaluated by aggregating the individual scores for inflammation, the degree of crypt damage, and the scope of lesions as previously described (Wang et al, 2018).

Immunostaining was performed as described previously (Tang et al, 2024). The following antibodies were used: anti-TRPV3 (1:400), anti-CK 18 (1:200), anti-occludin (1:400) and anti-E-cadherin (1:400). Fluorescent images were captured with Leica Stellaris 5 High-resolution Laser Confocal Fluorescence Microscope (Leica Microsystems, Germany).

### Myeloperoxidase (MPO) activity assay

The MPO enzymatic activity in mouse colon samples was assessed using an MPO activity assay kit as instructed by the manufacturer. Briefly, the supernatant of colonic homogenate was mixed with H_2_O_2_ and o-dianisidine. The resulting oxidized o-dianisidine was measured by taking the absorbance at 460 nm, which linearly correlates with the MPO activity. The MPO activities were reported in units per gram (U/g) of the wet weight of the tissue.

### Real time-quantitative polymerase chain reaction (RT-qPCR)

RT-qPCR was performed as previously described (Xue et al, 2023). Total RNA of the colon tissue was extracted using Trizol reagent (Cat# R401-01, Vazyme). Reverse-transcription was performed using the HiScript II Q RT SuperMix kit with anchored oligo (dT) 23 VN primer (Cat# R223-01, Vazyme). RT-qPCR was conducted on a QuantStudio 3 Real Time PCR system (Thermo Fisher Scientific, Massachusetts, USA). mRNA expression levels were quantified using the 2^-ΔΔCt^ method and normalized to GAPDH. All primers listed in Appendix Table S2 were synthesized by Beijing Tsingke Biotechnology Co., Ltd. (Beijing, China).

### Intestinal permeability assay

Intestinal permeability was assessed using FITC-dextran (MW 4000). Briefly, after a 4-h fast, mice received FITC-dextran (400 mg/kg) by oral gavage. Four hours later, blood was collected, and serum was obtained by centrifugation at 3000×*g* for 10 min at 4 °C. Fluorescence was measured at excitation 470–495 nm and emission 515–575 nm, and the serum FITC-dextran concentration was calculated from a standard curve generated with serial dilutions of FITC-dextran.

### Statistical analysis

Statistical analysis was performed using GraphPad Prism version 8.01 (San Diego, CA, USA). Data are presented as mean ± SEM. Statistical significance was assessed using Student’s *t* test, one-way ANOVA or two-way ANOVA, followed by Tukey’s or Dunnett’s multiple comparisons test. A *P* value of less than 0.05 is deemed statistically significant. The concentration-response curves were generated using non-linear regression analysis.

## Supplementary information

Appendix

Peer Review File

Dataset EV1

Source data Fig. 1

Source data Fig. 2

Source data Fig. 4

Source data Fig. 5

Figure EV Source Data

Expanded View Figures

## Data Availability

The raw data supporting the conclusions of this article will be made available by the authors, without undue reservation. The 3D cryo-EM density maps and coordinates of TRPV3_apo_, TRPV3_Colivin-1_, and TRPV3_Colivin-2_ have been deposited in the Electron Microscopy Data Bank (EMDB) under the accession numbers EMD-66282, EMD-66283 and EMD-66284 and the Protein Data Bank (PDB) under accession codes 9WV5, 9WV6 and 9WV7, respectively. The source data of this paper are collected in the following database record: biostudies:S-SCDT-10_1038-S44318-026-00896-9.
