# Supplementary material for: Orally available Colivin traps TRPV3 in an inactive state and suppresses intestinal inflammation
Source: EMBO J. 2026 Aug 13;45(18):6480–500. doi: 10.1038/s44318-026-00896-9 (PMC13578699; doi:10.1038/s44318-026-00896-9)
Supplement: Supplementary file 1 [file 44318_2026_896_MOESM1_ESM.pdf]

Appendix for

Orally available Colivin traps TRPV3 in an inactive state and suppresses intestinal inflammation

Content

Extended Methods 2

Synthesis of Compound Colivin 2

Liver Microsomal Stability Assay 4

Pharmacokinetics 4

Extended Data 5

Appendix Figure S1. 2-APB failed to activate Q580A and I583A mutants of hTRPV3.......... 5

Appendix Figure S2. Structural comparison of Colivin-bound TRPV3 with closed and inactivated TRPV3 states 6

Appendix Figure S3. Representative immunofluorescent images of colon tissues stained with TRPV3 (green) and cytokeratin 18 (CK18, red). 7

Appendix Figure S4. Synthesis route of Colivin. 8

Appendix Figure S5. ^{1}H NMR Spectrum of Colivin. 9

Appendix Figure S6. ^{13}C NMR Spectrum of Colivin. 10

Appendix Figure S7. HRMS Spectrum of Colivin. 11

Appendix Table S1. Primers used for site-directed mutagenesis 12

Appendix Table S2. Primers used for gene expression detection 13

Extended Methods

Synthesis of Compound Colivin

Reagents and conditions: (a) Pd(dppf)Cl_2, Cs_2CO_3, H_2O/1,4-Dioxane (1:6), sealed tube, 100 °C; (b) Pd(dppf)Cl_2, AcOK, 1,4-Dioxane, 90 °C; (c) Pd/C, H_2, Ethyl acetate/MeOH (1:1), rt. (d) Pd(dppf)Cl_2, Cs_2CO_3, H_2O/1,4-Dioxane (1:6), sealed tube, 110 °C; (e) $LiAlD_4$, dry THF, 0 °C; (f) Trifluoroacetic acid, Dichloromethane, rt; (g) 5-Fluoro-2-picolinic acid, HATU, DIPEA, Dichloromethane, rt.

The synthesis route of Colivin is depicted in **Appendix Fig. S4**.

***tert*-Butyl 3-(4-bromo-2-(methoxycarbonyl)phenyl)-2,5-dihydro-1*H*-pyrrole-1-carboxylate (Int-1)**

To a solution of methyl 5-bromo-2-iodobenzoatea (1.3 g, 3.81 mmol), *tert*-butyl 3-(4,4,5,5-tetramethyl-1,3,2-dioxaborolan-2-yl)-2,5-dihydro-1*H*-pyrrole-1-carboxylate (938 mg, 3.18 mmol), and Cs_2CO_3 (2.072 g, 6.36 mmol) in 36 mL of 1,4-dioxane and 6 mL water, Pd(dppf)Cl_2 (117 mg, 0.16 mmol) was added under a nitrogen atmosphere. The reaction mixture was then heated to 100 °C and stirred for 3 h. Upon completion of the reaction, the insoluble residue was removed by filtration, and the filtrate was concentrated under reduced pressure. The crude product was purified by flash chromatography to afford intermediate **Int-1** (927 mg, 64%) as an oil. ^{1}H NMR (500 MHz, Chloroform-*d*) δ 7.94 (dd, *J* = 3.8, 2.1 Hz, 1H), 7.60 (dd, *J* = 8.3, 2.1 Hz, 1H), 7.16 (dd, *J* = 8.3, 3.8 Hz, 1H), 5.74 – 5.65 (m, 1H), 4.39 – 4.23 (m, 4H), 3.88 (s, 3H), 1.49 (s, 9H). MS (ESI) *m/z*: 382.1 $[M + H]^+$.

***tert*-Butyl 3-(2-(methoxycarbonyl)-4-(4,4,5,5-tetramethyl-1,3,2-dioxaborolan-2-yl)phenyl)-2,5-dihydro-1*H*-pyrrole-1-carboxylate (Int-2)**

To a solution of **Int-1** (927 mg, 2.42 mmol), bis(pinacolato)diboron (938 mg, 3.18 mmol), and AcOK (477 mg, 4.87 mmol) in 40 mL of 1,4-dioxane, Pd(dppf)Cl_2 (179 mg, 0.24 mmol) was added under a nitrogen atmosphere. The reaction mixture was then heated to 90°C and stirred overnight. Upon completion of the reaction, the insoluble residue was removed by filtration, and the filtrate was concentrated under reduced pressure. The crude product was purified by flash chromatography to afford intermediate **Int-2** (1 g, 96%) as an oil. ^{1}H NMR (500 MHz, Chloroform-*d*) δ 8.26 – 8.20 (m, 1H), 7.95 – 7.86 (m, 1H), 7.39 (d, *J* = 7.9 Hz, 1H), 4.23 – 4.15 (m, 1H), 4.11 (qt, *J* = 7.0, 3.2 Hz, 1H), 3.90 (t, *J* = 2.8 Hz, 3H), 3.80 – 3.73 (m, 1H), 3.65 – 3.47 (m, 1H), 3.45 – 3.29 (m, 2H), 1.46 (d, *J* = 5.5 Hz, 9H), 1.34 (m, 12H). MS (ESI) *m/z*: 430.2 $[M + H]^+$.

***tert*-Butyl 3-(2-(methoxycarbonyl)-4-(4,4,5,5-tetramethyl-1,3,2-dioxaborolan-2-yl)phenyl)pyrrolidine-1-carboxylate (Int-3)**

Int-2 (1 g, 2.33 mmol) was dissolved in 20 mL of a methanol/ethyl acetate mixture (v/v = 1:1). To this solution, 10% palladium on carbon (200 mg) was added, and the reaction mixture was stirred under a hydrogen atmosphere at room temperature overnight. Upon completion, the mixture was filtered to remove the catalyst and concentrated to afford intermediate **Int-3** (920 mg, 92%) as an oil. MS (ESI): *m/z* 432.3 $[M + H]^+$.

***tert*-Butyl 3-(4'-fluoro-3-(methoxycarbonyl)-2'-(trifluoromethoxy)-[1,1'-biphenyl]-4-yl)pyrrolidine-1-carboxylate (Int-4)**

To a solution of **Int-3** (920 mg, 2.13 mmol), 4-fluoro-1-iodo-2-(trifluoromethoxy)benzene (544 mg, 1.78 mmol), and Cs_2CO_3 (1.16 g, 3.56 mmol) in 20 mL of 1,4-dioxane and 3 mL water, Pd(dppf)Cl_2 (132 mg, 0.18 mmol) was added under a nitrogen atmosphere. The reaction mixture was then heated to 110 °C and stirred for 3 h. Upon completion of the reaction, the insoluble residue was removed by filtration, and the filtrate was concentrated under reduced pressure. The crude product was purified by flash chromatography to afford intermediate **Int-4** (520 mg, 61%) as an oil. MS (ESI) *m/z*: 484.2 $[M + H]^+$.

***tert*-Butyl 3-(4'-fluoro-3-(hydroxymethyl-*d*₂)-2'-(trifluoromethoxy)-[1,1'-biphenyl]-4-yl)pyrrolidine-1-carboxylate (Int-5)**

To a solution of **Int-4** (520 mg, 1.08 mmol) in dry THF, $LiAlD_4$ (55 mg, 1.31 mmol) was added under a nitrogen atmosphere after cooling the solution to 0°C using an ice bath. The reaction mixture was maintained at 0°C and stirred for 30 min. Upon completion of the reaction, the mixture was cautiously quenched with water, extracted with ethyl acetate and washed with saturated brine. The organic layer was then dried over anhydrous $MgSO_4$, and the crude product was purified by flash chromatography to afford intermediate **Int-5** (420 mg, 85%) as an oil. MS (ESI) *m/z*: 458.2 $[M + H]^+$.

(4'-Fluoro-4-(pyrrolidin-3-yl)-2'-(trifluoromethoxy)-[1,1'-biphenyl]-3-yl)methan-*d*₂-ol (Int-6)

Int-5 (420 mg, 0.92 mmol) was dissolved in dichloromethane (3 mL), and 1 mL of trifluoroacetic acid was added to the solution. The mixture was stirred at room temperature for 30 min. Upon completion of the reaction, the solvent was removed under reduced pressure, yielding the crude product **Int-6** (307 mg, 93%) which is used directly for next step. MS (ESI) *m/z*: 358.1 $[M + H]^+$.

(3-(4'-Fluoro-3-(hydroxymethyl-*d*₂)-2'-(trifluoromethoxy)-[1,1'-biphenyl]-4-yl)pyrrolidin-1-yl)(5-fluoropyridin-2-yl)methanone (Colivin)

A solution was prepared by dissolving 5-fluoropyridine carboxylic acid (146 mg, 1.03 mmol), HATU (490 mg, 1.3 mmol), and DIPEA (449 µL, 2.58 mmol) in 15 mL of dichloromethane, and the mixture was stirred at room temperature for 30 min. Subsequently, a solution of **Int-6** (307 mg, 0.86 mmol) in dichloromethane was added, and the reaction mixture was stirred for an additional 2 h. Upon completion of the reaction, the mixture was concentrated under reduced pressure, and the crude product was purified by preparative HPLC using a gradient of acetonitrile/water (0.1% TFA) to afford the target compound Colivin (45 mg, 11%) as a white solid. ^1H NMR (500 MHz, DMSO-d_6, major isomer of the 4:3 stereoisomers) (**Appendix Fig. S5**) δ 8.59 (d, 1H, J = 2.5), 7.94 – 7.89 (m, 2H), 7.57 (dd, J = 8.5, 6.5 Hz, 1H), 7.51 – 7.45 (m, 2H), 7.44 (d, J = 2.0 Hz, 1H), 7.35 (dd, J = 8.5, 2.0 Hz, 1H), 5.14 (s, 1H), 4.10 – 4.04 (m, 1H), 3.85 – 3.79 (m, 1H), 3.72 – 3.68 (m, 2H), 3.66 – 3.59 (m, 1H), 2.30 – 2.22 (m, 1H), 2.15 – 2.07 (m, 1H). ^{13}C NMR (126 MHz, DMSO-d_6, major isomer of the 4:3 stereoisomers) (**Appendix Fig. S6**) δ 164.73 (C=O), 161.67 (d, J = 248.0 Hz), 159.87 (d, J = 257.5 Hz), 150.91 (d, J = 3.9 Hz), 145.90 (d, J = 10.7 Hz), 140.91, 139.01, 136.64 (d, J = 24.2 Hz), 133.77, 132.37 (d, J = 9.3 Hz), 131.52, 128.93, 128.32, 126.43, 126.26 (d, J = 5.3 Hz), 124.59 (d, J = 18.6 Hz), 120.31 (q, J = 258.4 Hz, -CF3), 115.58 (d, J = 20.8 Hz), 109.94 (d, J = 25.6 Hz), 60.78 (p, J = 20.3 Hz), 55.76, 47.15, 37.29, 31.18. HRMS (ESI) (**Appendix Fig. S7**) *m/z*: $[M + H]^+$ calcd for $C_{24}H_{18}D_2F_5N_2O_3^+$, 481.1514; found, 481.1510. HPLC purity: 99.53%.

Liver Microsomal Stability Assay

Live microsomal stability was conducted by incubating microsomes (0.33 mg/mL final concentration) in 0.1 M TRIS buffer (pH 7.4) supplemented with 5 mM $MgCl_2$ as a cofactor. Test compounds were introduced at a final concentration of 1 µM, along with cosolvents (0.01% DMSO and 0.005% bovine serum albumin). After a 10-minute pre-incubation at 37 °C, the reaction was initiated by adding NADPH to a final concentration of 1 mM. Aliquots were taken at 0, 5, 15, 30, and 60 minutes and quenched immediately with ice-cold methanol. The samples were centrifuged at 4,000 rpm for 5 min, and the resulting supernatant was analyzed by LC–MS/MS.

Pharmacokinetics

Colivin was dissolved in a formulation containing 2% DMSO, 2% Tween-80 and 0.5% CMC-Na solution. After oral gavage of 10 mg/kg Colivin to ICR mice (Shanghai Shengchang Biotechnology Co., Ltd.), blood samples were collected at 0.25, 0.5, 1, 2, 4, 8, and 12 h. Plasma was separated by centrifugation and stored at -70 °C. The concentration of the parent drug in plasma was quantified using LC-MS/MS (AB Sciex, CA, USA), and pharmacokinetic parameters including T_{max}, C_{max}, AUC_{0-t}, and $t_{1/2}$ were calculated using WinNonlin software (Certara, NJ, USA), summarized in **Table 2**.

Extended Data

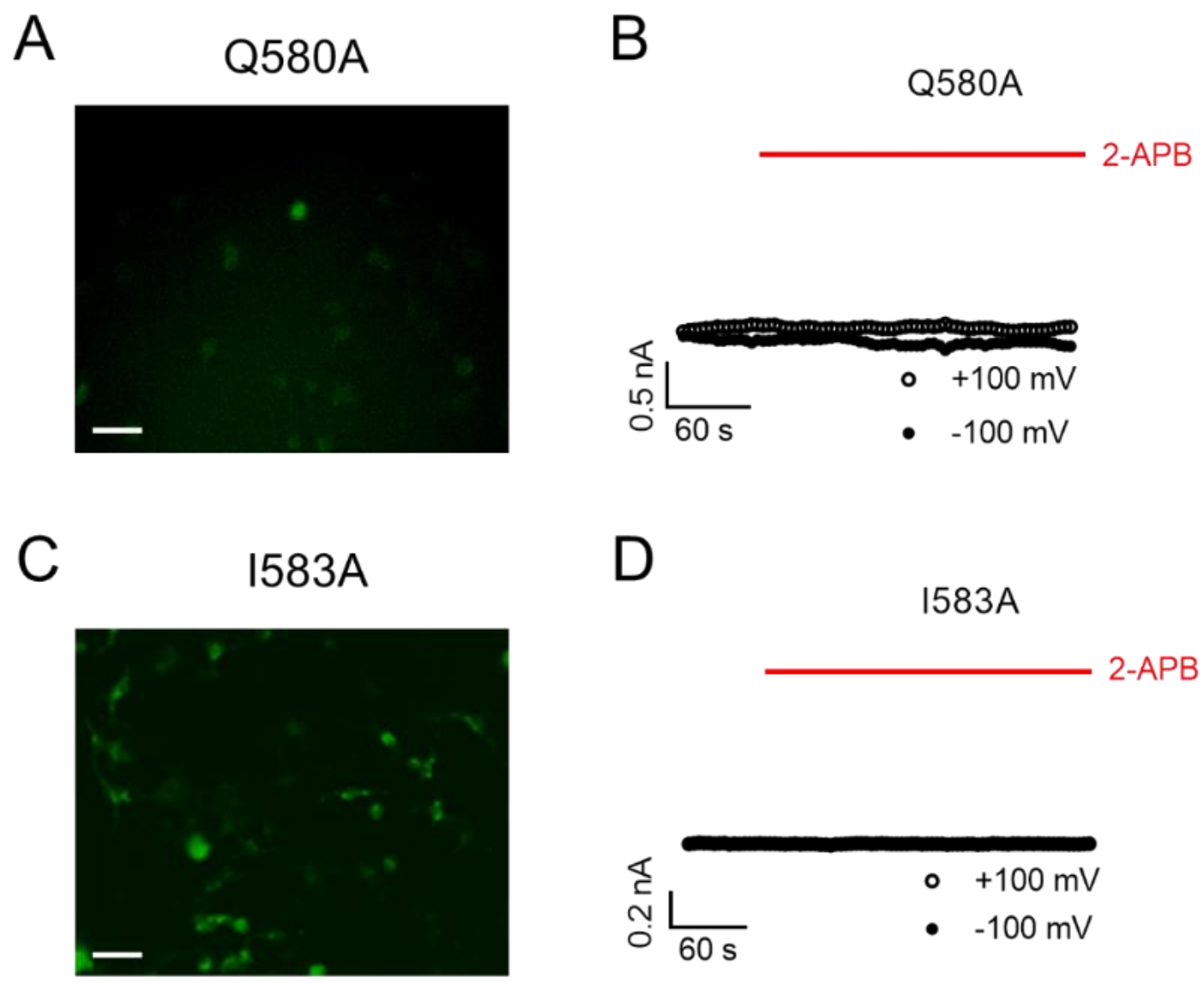

Appendix Figure S1. 2-APB failed to activate Q580A and I583A mutants of hTRPV3. Representative fluorescence micrographs of HEK-293 cells transfected with plasmids encoding Q580A **(A)** and I583A **(C)** hTRPV3 mutants. The successful expression of the encoded protein was confirmed by the green fluorescence. Scale bar = 25 µm. Representative whole-cell current traces at ±100 mV following bath application of 2-APB (100 µM) in HEK-293 cells transiently expressing Q580A **(B)** and I583A **(D)** mutants. Related to **Figure 2**.

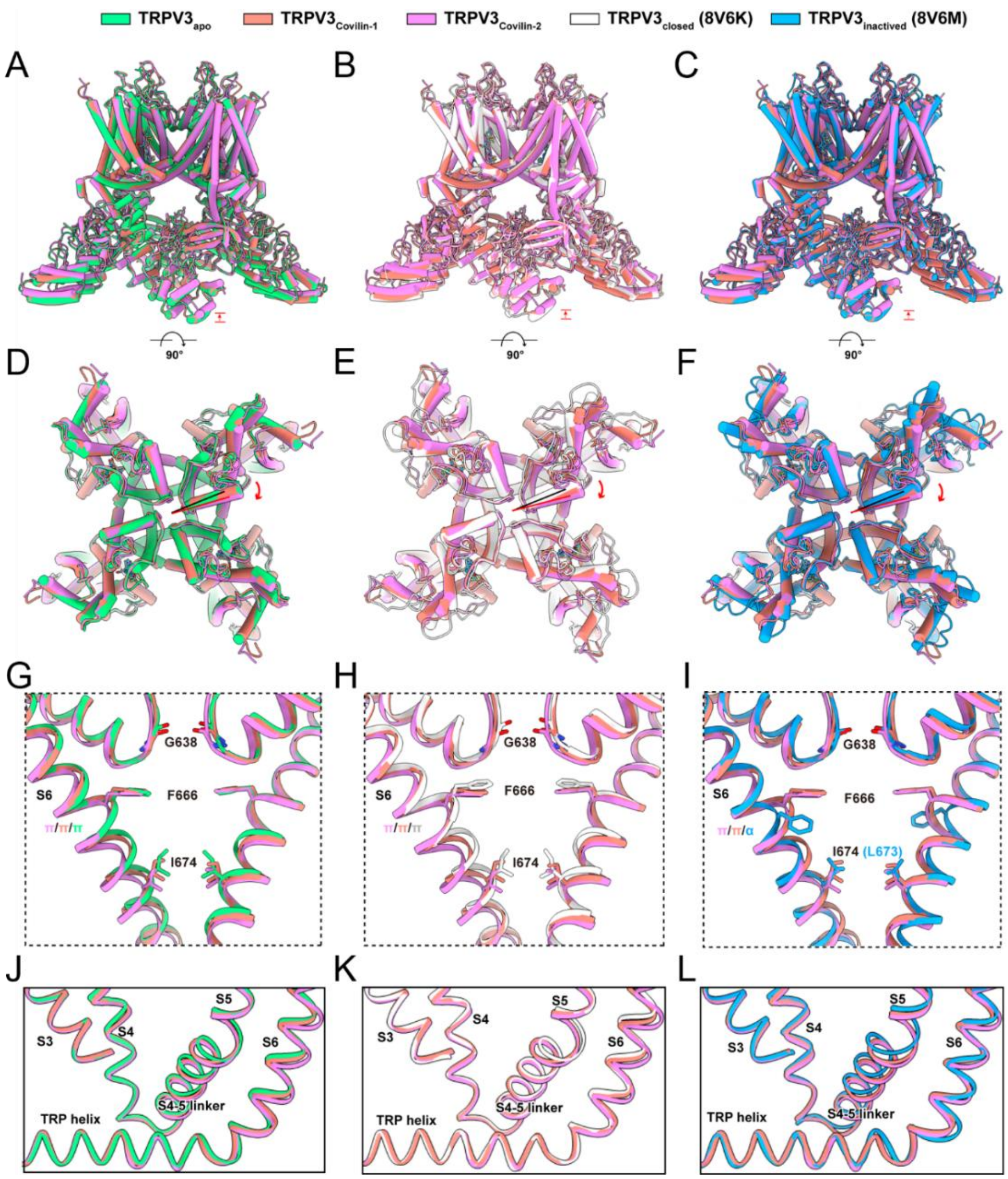

Appendix Figure S2. Structural comparison of Colivin-bound TRPV3 with closed and inactivated TRPV3 states

(A-C) Side views of structural overlays of $TRPV3_{Colivin-1}$ (light salmon) and $TRPV3_{Colivin-2}$ (violet) with $TRPV3_{apo}$ (spring green) (**A**), closed TRPV3 (white; PDB ID: 8V6K) (**B**), and inactivated TRPV3 (blue; PDB ID: 8V6M) (**C**). Red arrows indicate conformational shifts observed in $TRPV3_{Colivin-2}$. **(D-F)** Top-down views of the same structural overlays. Red arrows indicate conformational shifts observed in $TRPV3_{Colivin-2}$. **(G-I)** Boxed regions show pore-forming domains, with residues contributing to pore lining shown as sticks. Only two of the four subunits are shown, with the front and back subunits omitted for clarity. **(J-L)** Boxed regions show the transmembrane domains.

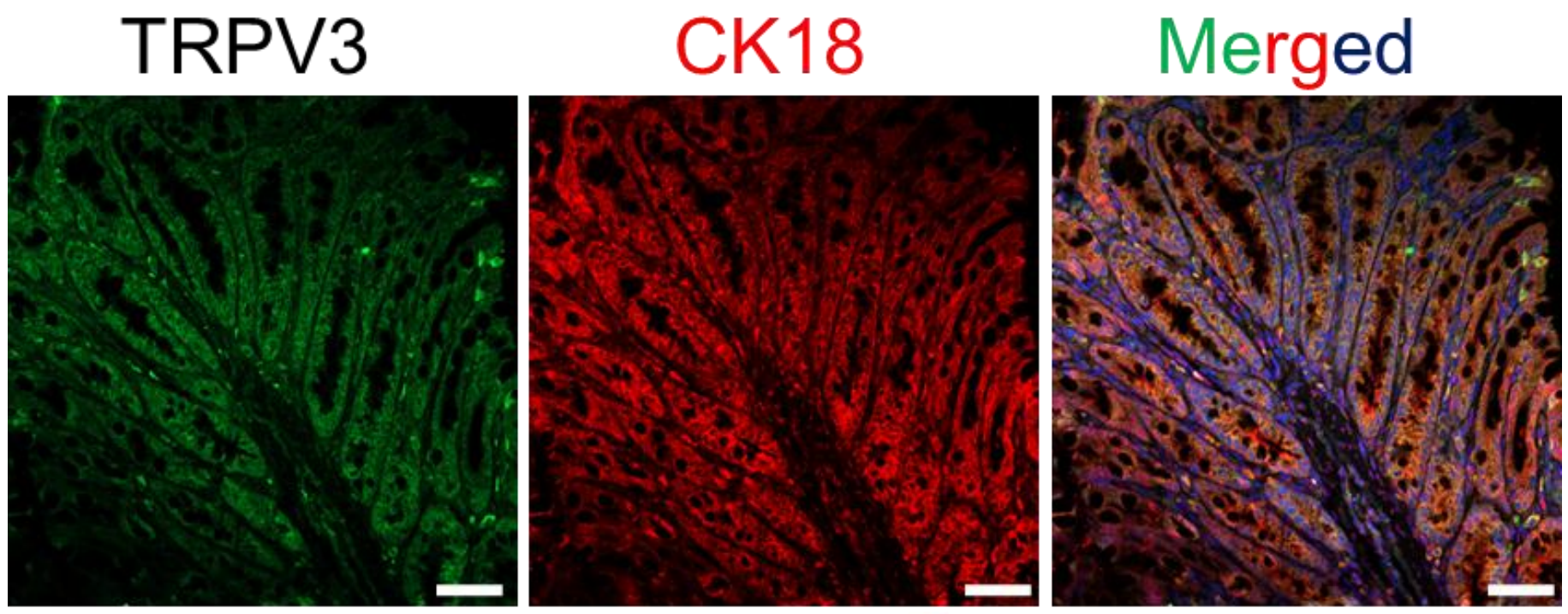

Appendix Figure S3. Representative immunofluorescent images of colon tissues stained with TRPV3 (green) and cytokeratin 18 (CK18, red).

Nuclei were stained by Hoechst 33342 (blue). Scale bar = 50 μm.

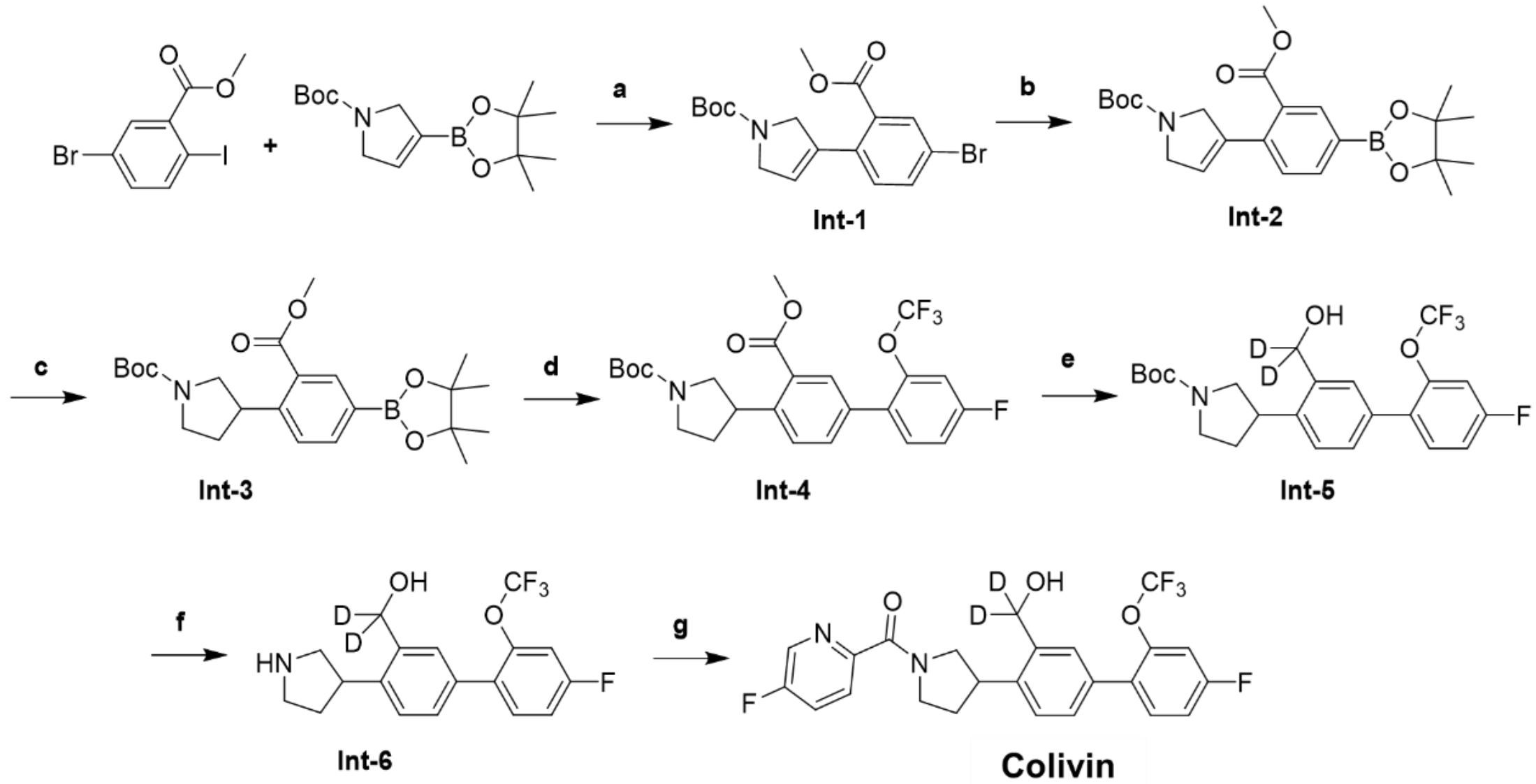

Appendix Figure S4. Synthesis route of Colivin.

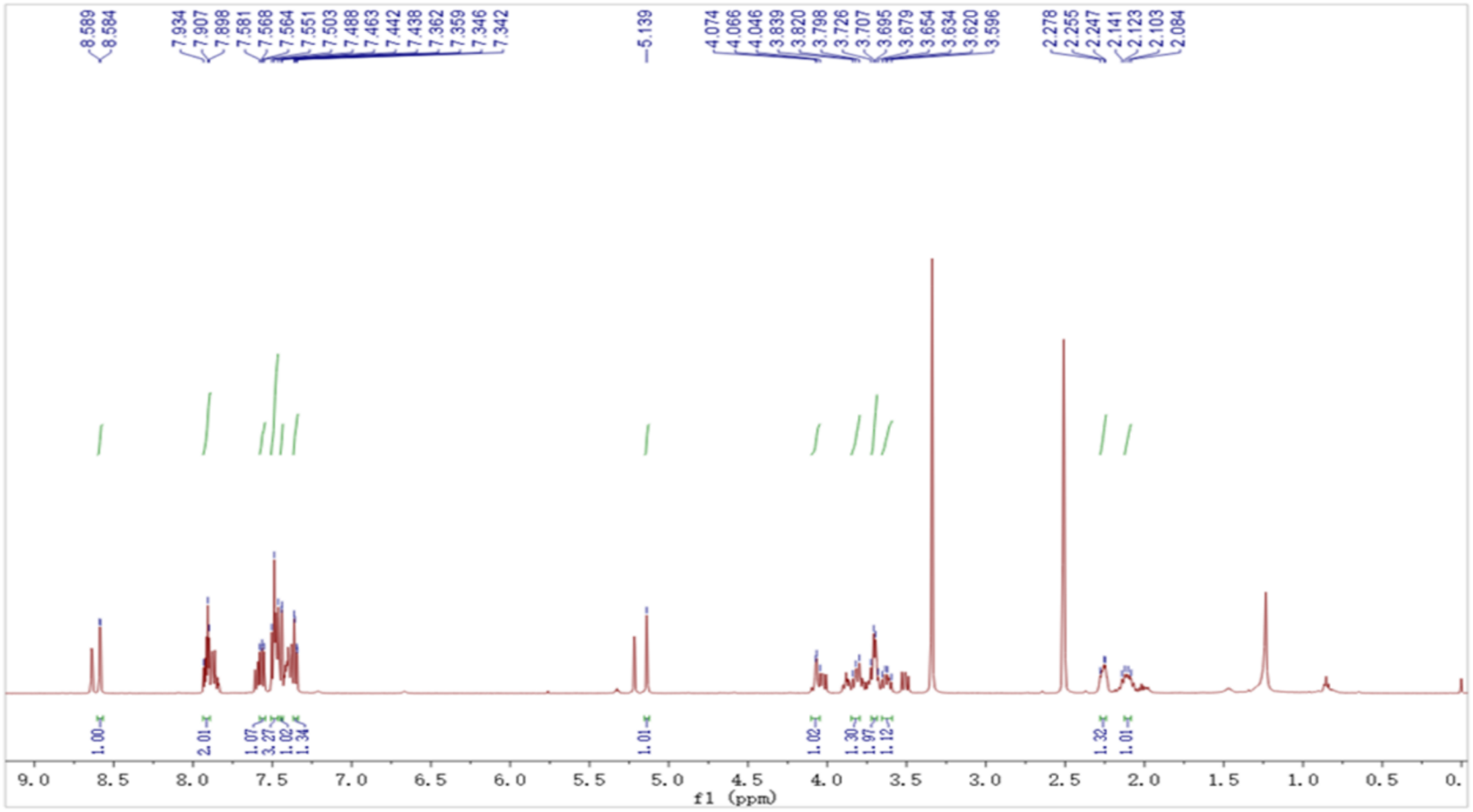

Appendix Figure S5. ^{1}H NMR Spectrum of Colivin.

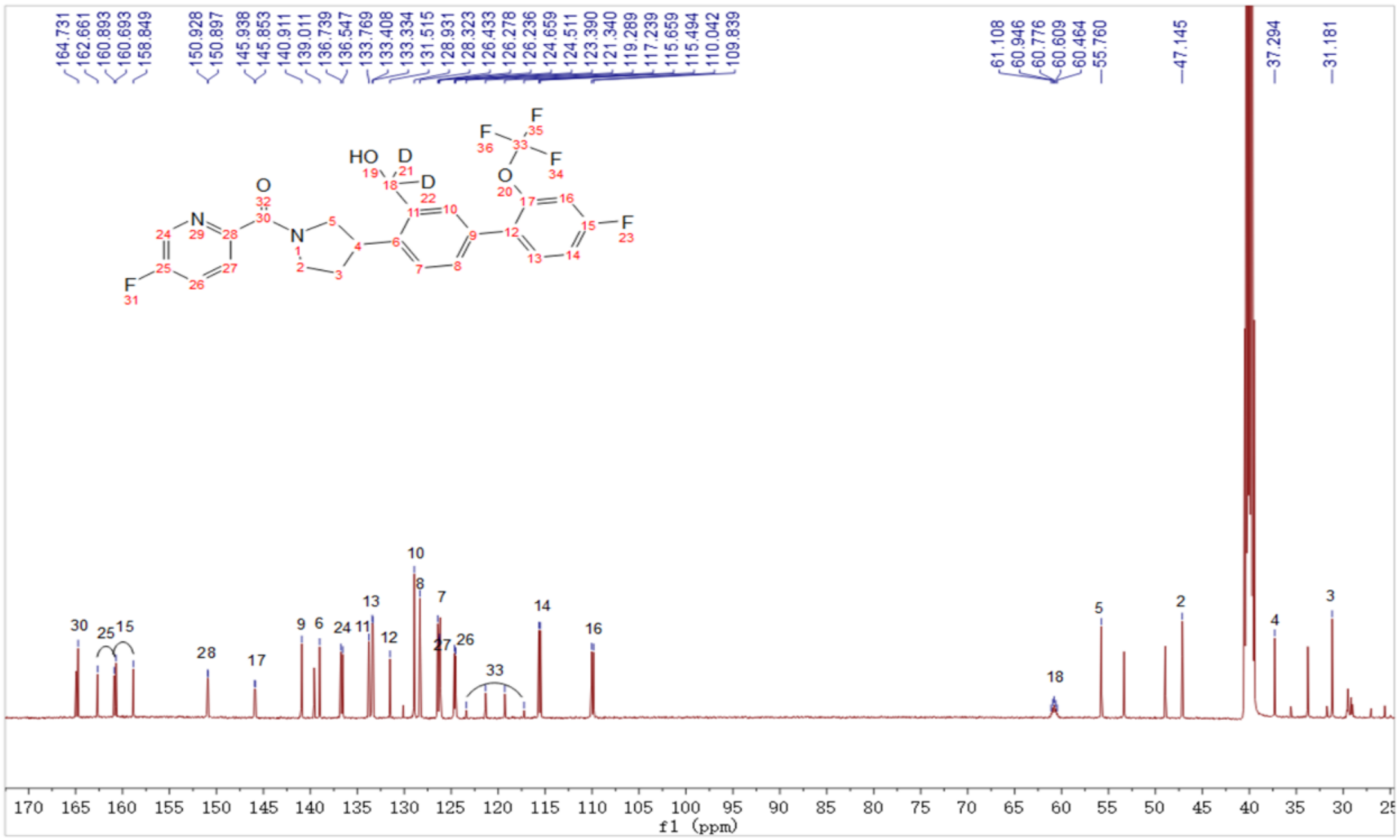

Appendix Figure S6. ^{13}C NMR Spectrum of Colivin.

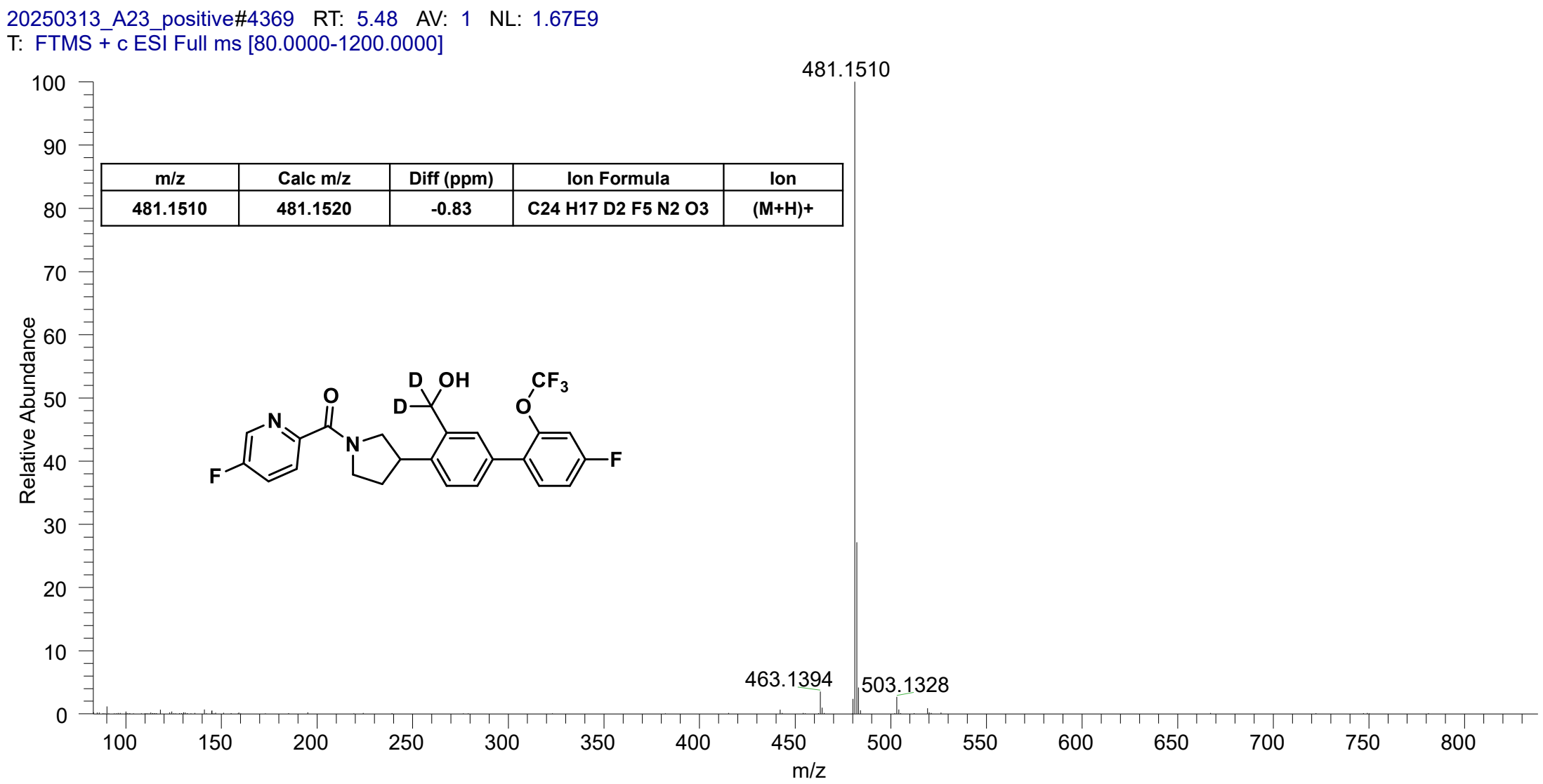

Appendix Figure S7. HRMS Spectrum of Colivin.

Appendix Table S1. Primers used for site-directed mutagenesis

Mutation	Primer	Sequence (5'− 3')
I516A	Forward	AGTCCGCACTCTCGGATGCCTGGTTCCACTTTG
	Reverse	GAGAGTGCGGACTGCAGATCCGAGGGTCTCAGC
W521A	Forward	CTCGGATGCCGCGTTCCACTTTGTCTTTTTATCC
	Reverse	AAAGTGGAACGCGGCATCCGAGAGGATGGACTGCA
F522A	Forward	GCCTGGGCACACTTTGTCTTTTTATCC
	Reverse	AAAGTGTGCCCAGGCATCCGAGAGGATG
L557A	Forward	TGGCCATGGCCGCTGGCTGGGCGAACATGCTCTACTATAC
	Reverse	TTCGCCCAGCCAGCGGCCATGGCCAGCACGAGGCAGGCGA
A560F	Forward	CCTGGGCTGGTTTAACATGCTCTACTATACGCGG
	Reverse	AGAGCATGTTAAACCAGCCCAGGGCCATGGCCAG
N561A	Forward	ATGGCCCTGGGCTGGGCGGCTATGCTCTACTATA
	Reverse	TATAGTAGAGCATAGCCGCCCAGCCCAGGGCCAT
L563A	Forward	GCGAACATGGCATACTATACGCGGGGTTTCC
	Reverse	CGTATAGTATGCCATGTTCGCCCAGCCCAGG
Q580A	Forward	GTCATGATCGCCAAGGTCATTTTGCATG
	Reverse	AATGACCTTGGCGATCATGACGCTGTAC
I583A	Forward	GAAGGTCGCTTTGCATGATGTTCTG
	Reverse	ATGCAAAGCGACCTTCTGGATCATG
L584A	Forward	GGTCATTGCGCATGATGTTCTGAAGTTC
	Reverse	ATCATGCGCAATGACCTTCTGGATCATG
F601A	Forward	TTTGCTTGGAGCTGGAGTAGCCTTGGC
	Reverse	GGCTACTCCAGCTCCAAGCAAAAACAC
F656A	Forward	TTTCTGGCCCTGCTCATCACCTATG
	Reverse	AGCAGGGCCAGAAAGAGAATGGGAT
L664A	Forward	ATGTCATCGCCACCTTTGTTCTCCTC
	Reverse	CAAAGGTGGCGATGACATAGGTGATG

Appendix Table S2. Primers used for gene expression detection

Genes	Sense (5’ − 3’)	Anti-sense (5’ − 3’)
Gapdh	CATCTTCCAGGAGCGAGACC	GAAGGGGCGGAGATGATGAC
Tnf	GACGTGGAACTGGCAGAAGAG	TGCCACAAGCAGGAATGAGA
Cxcl1	GCTGGGATTCACCTCAAGAA	CTTGGGGACACCTTTTAGCA
Il6	GTTCTCTGGGAAATCGTGGA	TTCTGCAAGTGCATCATCGT
Il1b	GAAATGCCACCTTTTGACAGTG	TGGATGCTCTCATCAGGACAG
