# Supplementary material for: Orally available Colivin traps TRPV3 in an inactive state and suppresses intestinal inflammation
Source: EMBO J. 2026 Aug 13;45(18):6480–500. doi: 10.1038/s44318-026-00896-9 (PMC13578699; doi:10.1038/s44318-026-00896-9)
Supplement: Supplementary file 2 [file 44318_2026_896_MOESM2_ESM.pdf]

THE EMBO JOURNAL

Orally available Colivin traps TRPV3 in an inactive state and suppresses intestinal inflammation

Yujing Wang, Jiahui Chen, Qinglian Tang, Xu Qin, Shuxian Wang, Chuchu Xi, Zhaowen Lu, Kerui Ren, Chu Xue, Jianhua Shen, Kai Wang, Michael Zhu, Jie Yu, and Zhengyu Cao

Corresponding author(s): Zhengyu Cao (zycao@cpu.edu.cn) , Kai Wang (kwang@simm.ac.cn), Jie Yu (yujie@sioc.ac.cn)

**Review Timeline:**		
	Submission Date:	25th Nov 25
	Editorial Decision:	4th Dec 25
	Appeal Received:	9th Jan 26
	Editorial Decision:	26th Mar 26
	Revision Received:	10th Jun 26
	Editorial Decision:	6th Jul 26
	Revision Received:	10th Jul 26
	Accepted:	21st Jul 26

Editor: William Teale

Transaction Report:

(Note: With the exception of the correction of typographical or spelling errors that could be a source of ambiguity, letters and reports are not edited. Depending on transfer agreements, referee reports obtained elsewhere may or may not be included in this compilation. Referee reports are anonymous unless the Referee chooses to sign their reports.)

1st Editorial Decision **4th Dec 2025**

Dear Prof. Cao,

Thank you for submitting your manuscript entitled 'Mechanism of Colivin Suppression of TRPV3 to Ameliorate Ulcerative Colitis' (EMBOJ-2025-123133) to our editorial office. We have now considered the study within our editorial team, and unfortunately come to the conclusion that we cannot offer publication in The EMBO Journal. As you carefully explain TRPV3 (a warm-sensitive Ca2+-permeable cation channel) influences the integrity of epidermal barriers by regulating inflammation. In the current manuscript, you examine the properties of Colivin: a derivative of a known TRPV3 inhibitor with more promising properties for oral administration. You find that Colivin binds TRPV3 in an unreported mode, giving new clues as to the mechanism of TRPV3 inhibition. Furthermore, Colivin improved DSS-induced colitis in a manner similar to the TRPV3 loss-of-function mutant. These data are likely to be of interest to the field. However, without a closer examination of the effect of Colivin on DSS-induced colitis in trpv3 loss-of-function mice, your analysis may not be a strong candidate for publication in a broad general journal like The EMBO Journal at this stage.

However, given the potential value of your results to researchers directly in the field, we feel that the study should be well-suited for Life Science Alliance (http://www.life-science-alliance.org/), our open access journal launched in partnership with Rockefeller University Press and Cold Spring Harbor Laboratory Press. LSA aims to publish solid findings of value to particular communities across all areas of biology. I have therefore briefly discussed the work with the editors of Life Science Alliance, who would indeed be pleased to send your work for in-depth external review, without need for prior reformatting. Should you be interested in this option, please simply follow the link below for transfer. Tim Fessenden, Executive Editor of Life Science Alliance (t.fessenden@life-science-alliance.org), will be happy to answer any questions you may have.

I am sorry that I cannot be more positive for The EMBO Journal on this occasion, but very much hope that you will find this transfer option worthwhile.

Yours sincerely,

William Teale

William Teale, PhD
Editor
The EMBO Journal
w.teale@embojournal.org

** As a service to authors, EMBO Press provides authors with the possibility to transfer a manuscript that one journal cannot offer to publish to another EMBO publication or the open access journal Life Science Alliance launched in partnership between EMBO Press, Rockefeller University Press and Cold Spring Harbor Laboratory Press. The full manuscript and if applicable, reviewers' reports, are automatically sent to the receiving journal to allow for fast handling and a prompt decision on your manuscript. For more details of this service, and to transfer your manuscript please click on Link Not Available. **

Please do not share this URL as it will give anyone who clicks it access to your account.

中国药科大学
China Pharmaceutical University
Nanjing, P.R. China

State Key Laboratory of Natural Medicines
China Pharmaceutical University
639, Longmian Ave, Nanjing, China, 211198
Tel: 025-86185158

Jan. 09, 2026
Dr. Facundo D. Batista
Chief Editor
The EMBO Journal

Dear Dr. Facundo D. Batista

I write to request your permission to allow for submission of a revised manuscript of our original research article (MS ID# EMBOJ-2025-123133) entitled "**Mechanism of Colivin Suppression of TRPV3 to Ameliorate Ulcerative Colitis**" by Wang *et al*. to the *EMBO Journal* for publication.

We thank the Executive Editor (Dr. William Teale) for his careful evaluation and constructive comments on our manuscript. These comments are overall quite positive, and the suggested additional experiments can be done within a short period of time.

The editor remarked: "*In the current manuscript, you examine the properties of Colivin: a derivative of a known TRPV3 inhibitor with more promising properties for oral administration. You find that Colivin binds TRPV3 in an unreported mode, giving new clues as to the mechanism of TRPV3 inhibition. Furthermore, Colivin improved DSS-induced colitis in a manner similar to the TRPV3 loss-of-function mutant. These data are likely to be of interest to the field. However, without a closer examination of the effect of Colivin on DSS-induced colitis in Trpv3 loss-of-function mice, your analysis may not be a strong candidate for publication in a broad general journal like The EMBO Journal at this stage*." Specifically, the editor asked us to conduct additional experiments to verify the engagement of TRPV3 in Colivin suppression of ulcerative colitis.

In the **original Fig. 4**, we demonstrated that the ablation of *Trpv3* attenuates DSS-induced ulcerative colitis. To address the concern raised by the editor, we have investigated the therapeutic effect of Colivin on ulcerative colitis in wild-type and *Trpv3*-deficient mice. As shown here in **Fig. R1**, *Trpv3* gene deletion significantly ameliorated DSS-induced colitis, as evidenced by decreased weight loss, lower DAI scores, reduced colon atrophy, improved histopathology and attenuated inflammation. However, oral administration of Colivin did not confer any further protective effect in *Trpv3*-deficient mice. We have replaced the **original Fig. 4** with **Fig. R1**, which is now **Fig. 5**.

Therefore, although the original manuscript was not sent to review, the positive comments of the editor and our successful acquisition of new data to address the concerns encouraged us to submit a revised manuscript.

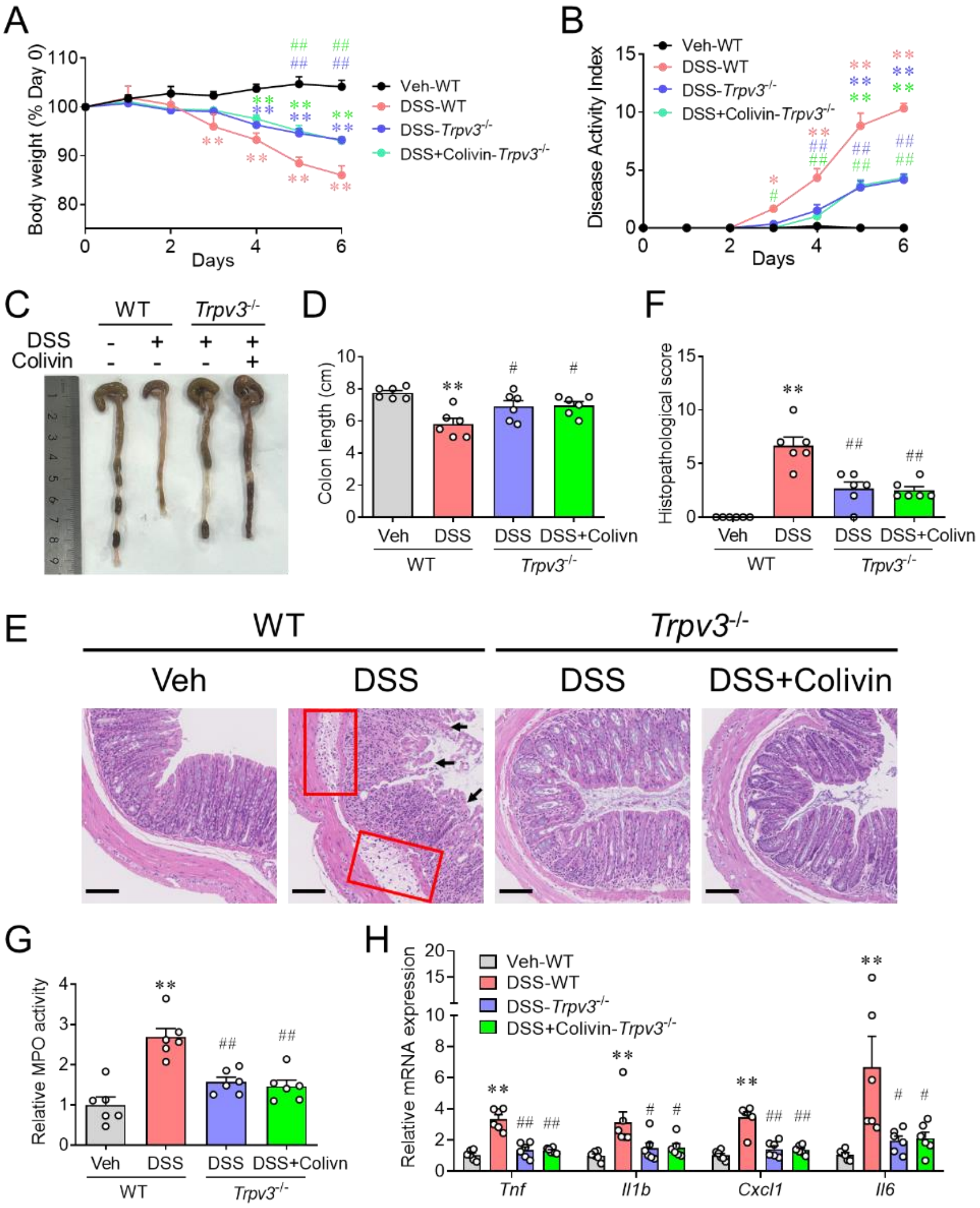

Fig. R1. Engagement of TRPV3 on Colivin ameliorated ulcerative colitis.

(A-B) Changes in body weight **(A)** and disease activity index **(B)** of WT mice treated with Vehicle (Veh) or DSS and *Trpv3*$^{-/-}$ mice treated with DSS or DSS plus Colivin (10 mg/kg, *i.g.* twice daily) for 6 days. Data represent mean ± SEM (N = 6 mice); **, $P < 0.01$, *vs.* Veh-treated WT mice; $^{\#}$, $P < 0.05$, $^{\#\#}$, $P < 0.01$, *vs.* DSS-WT, by Two-way ANOVA with Dunnett's multiple comparisons tests. **(C-D)** Representative colon images **(C)** and quantification of colon length **(D)** in WT mice treated with Vehicle (Veh) or DSS and *Trpv3*$^{-/-}$ mice treated with DSS or DSS plus Colivin. **(E)** Representative micrographs of colon tissues from WT and *Trpv3*$^{-/-}$ mice stained with H&E. Disappearance of crypts and edematous lamina propria containing inflammatory cell infiltrates are indicated by black arrows and red boxes, respectively. Scale bars = 100 μm. **(F)** Quantification of histopathological scores of H&E-stained colon tissues from WT and *Trpv3*$^{-/-}$ mice derived from (E). **(G)** MPO activity in colon tissues from WT and *Trpv3*$^{-/-}$ mice with different treatments. **(H)** Quantification of mRNA levels of *Tnf*, *Il1b*, *Cxcl1*, *Il6* in colon tissues from WT and *Trpv3*$^{-/-}$ mice with different treatments. Data represent mean ± SEM for N = 6 mice; **, $P < 0.01$, *vs.* Veh-treated WT mice; $^{\#}$, $P < 0.05$, $^{\#\#}$, $P < 0.01$, *vs.* DSS-treated WT mice, by One-way ANOVA with Bonferroni's multiple comparisons tests.

We confirm that the material is original research, has not been previously published, and is not currently being considered for publication elsewhere. Authors declare no conflict of interest. All authors have approved the manuscript for resubmission.

Thank you for considering our resubmitted manuscript for publication in *EMBO Journal*.

Sincerely

Prof. Zhengyu Cao, Ph.D.
State Key Laboratory of Natural Medicines
China Pharmaceutical University

Dear Prof. Cao,

Thank you again for the submission of your manuscript entitled "Mechanism of Colivin Suppression of TRPV3 to Ameliorate Ulcerative Colitis" and for your patience during the review process. We have now received reports from three referees, which I copy below.

As you can see from their comments, all thought that your manuscript could represent a useful and timely development to our understanding of the causes of colitis. That said, all of them point out areas that need to be strengthened with additional experimentation. This will require your attention before your manuscript can be published in The EMBO Journal.

Based on the overall interest expressed in the reports, however, I would like to invite you to address the comments of all referees in a revised version of the manuscript. I should add that it is The EMBO Journal policy to allow only a single major round of revision and that it is therefore important to resolve the main concerns at this stage. I believe the concerns of the referees are reasonable and addressable, but please contact me if you have any questions, need further input on the referee comments or if you anticipate any problems in addressing any of their points. I am available to discuss you revision plan by Zoom. Please, follow the instructions below when preparing your manuscript for resubmission.

I would also like to point out that as a matter of policy, competing manuscripts published during this period will not be taken into consideration in our assessment of the novelty presented by your study ("scooping" protection). We have extended this 'scooping protection policy' beyond the usual 3 month revision timeline to cover the period required for a full revision to address the essential experimental issues. Please contact me if you see a paper with related content published elsewhere to discuss the appropriate course of action.

When preparing your letter of response to the referees' comments, please bear in mind that this will form part of the Review Process File, and will therefore be available online to the community. For more details on our Transparent Editorial Process, please visit our website: https://link.springer.com/journal/44318/submission-guidelines#cms-Revised-submissions

Again, please contact me at any time during revision if you need any help or have further questions.

Thank you very much again for the opportunity to consider your work for publication. I look forward to your revision.

Best regards,

William Teale

William Teale, Ph.D.
Editor
The EMBO Journal

When submitting your revised manuscript, please carefully review the instructions below and include the following items:

1) a .docx formatted version of the manuscript text (including legends for main figures, EV figures and tables). Please make sure that the changes are highlighted to be clearly visible.

2) individual production quality figure files as .eps, .tif, .jpg (one file per figure).

3) a .docx formatted letter INCLUDING the reviewers' reports and your detailed point-by-point response to their comments. As part of the EMBO Press transparent editorial process, the point-by-point response is part of the Review Process File (RPF), which will be published alongside your paper.

4) a complete author checklist, which you can download from our author guidelines (https://link.springer.com/journal/44318/submission-guidelines#cms-Revised-submissions). Please insert information in the checklist that is also reflected in the manuscript. The completed author checklist will also be part of the RPF.

5) Please note that all corresponding authors are required to supply an ORCID ID for their name upon submission of a revised manuscript.

6) We require a 'Data Availability' section after the Materials and Methods. Before submitting your revision, primary datasets produced in this study need to be deposited in an appropriate public database, and the accession numbers and database listed under 'Data Availability'. Please remember to provide a reviewer password if the datasets are not yet public (see

https://link.springer.com/partners/embo-press/editorial-policies#Data%20deposition). If no data deposition in external databases is needed for this paper, please then state in this section: This study includes no data deposited in external repositories. Note that the Data Availability Section is restricted to new primary data that are part of this study.

Note - All links should resolve to a page where the data can be accessed.

7) When assembling figures, please refer to our figure preparation guideline in order to ensure proper formatting and readability in print as well as on screen:
https://link.springer.com/journal/44318/submission-guidelines#cms-Figure-and-data-presentation

Please remember: Digital image enhancement is acceptable practice, as long as it accurately represents the original data and conforms to community standards. If a figure has been subjected to significant electronic manipulation, this must be noted in the figure legend or in the 'Materials and Methods' section. The editors reserve the right to request original versions of figures and the original images that were used to assemble the figure.

8) For data quantification: please specify the name of the statistical test used to generate error bars and P values, the number (n) of independent experiments (specify technical or biological replicates) underlying each data point and the test used to calculate p-values in each figure legend. The figure legends should contain a basic description of n, P and the test applied. Graphs must include a description of the bars and the error bars (s.d., s.e.m.).

9) We would also encourage you to include the source data for figure panels that show essential data. Numerical data can be provided as individual .xls or .csv files (including a tab describing the data). For 'blots' or microscopy, uncropped images should be submitted (using a zip archive or a single pdf per main figure if multiple images need to be supplied for one panel). Additional information on source data and instruction on how to label the files are available at
https://link.springer.com/journal/44318/submission-guidelines#cms-Source-data

10) We replaced Supplementary Information with Expanded View (EV) Figures and Tables that are collapsible/expandable online (see examples in https://www.embopress.org/doi/10.15252/embj.201695874). A maximum of 5 EV Figures can be typeset. EV Figures should be cited as 'Figure EV1, Figure EV2" etc. in the text and their respective legends should be included in the main text after the legends of regular figures.

- For the figures that you do NOT wish to display as Expanded View figures, they should be bundled together with their legends in a single PDF file called *Appendix*, which should start with a short Table of Content. Appendix figures should be referred to in the main text as: "Appendix Figure S1, Appendix Figure S2" etc. See detailed instructions regarding expanded view here: < https://link.springer.com/journal/44318/submission-guidelines#cms-Expanded-View-data >.

- Additional Tables/Datasets should be labelled and referred to as Table EV1, Dataset EV1, etc. Legends have to be provided in a separate tab in case of .xls files. Alternatively, the legend can be supplied as a separate text file (README) and zipped together with the Table/Dataset file.

11) At EMBO Press we ask authors to provide source data for the main manuscript figures. Our source data coordinator will contact you to discuss which figure panels we would need source data for and will also provide you with helpful tips on how to upload and organize the files.

12) Our journal encourages inclusion of *data citations in the reference list* to directly cite datasets that were re-used and obtained from public databases. Data citations in the article text are distinct from normal bibliographical citations and should directly link to the database records from which the data can be accessed. In the main text, data citations are formatted as follows: "Data ref: Smith et al, 2001" or "Data ref: NCBI Sequence Read Archive PRJNA342805, 2017". In the Reference list, data citations must be labeled with "[DATASET]". A data reference must provide the database name, accession number/identifiers and a resolvable link to the landing page from which the data can be accessed at the end of the reference. Further instructions are available at < https://link.springer.com/journal/44318/submission-guidelines#cms-Reference-guidelines >.

13) In order to increase the reproducibility and reach of your work, The EMBO Journal includes a table of reagents that were used in the study. Please provide this along with your revisions.

Further instructions for preparing your revised manuscript:

Read our guidance for manuscript revisions and related editorial policies: https://link.springer.com/journal/44318/submission-guidelines#cms-Revised-submissions

Please make sure you upload a letter of response to the referees' comments together with the revised manuscript.

Please also check that the title and abstract of the manuscript are brief, yet explicit, even to non-specialists.

When assembling figures, please refer to our figure preparation guideline in order to ensure proper formatting and readability in print as well as on screen:
https://media.springernature.com/original/springer-cms/rest/v1/content/27825798/data/v1

At EMBO Press we ask authors to provide source data for the main manuscript figures. You will receive a separate email with instructions for providing source data with your revised manuscript, including how to upload and organize the files.

IMPORTANT: When you send the revision we will require
- a point-by-point response to the referees' comments, with a detailed description of the changes made (as a word file).
- a word file of the manuscript text.
- individual production quality figure files (one file per figure)
- a complete author checklist
- Expanded View files (replacing Supplementary Information)
- a Reagents and Tools Table as part of the Methods section

Please remember: Digital image enhancement is acceptable practice, as long as it accurately represents the original data and conforms to community standards. If a figure has been subjected to significant electronic manipulation, this must be noted in the figure legend or in the 'Materials and Methods' section. The editors reserve the right to request original versions of figures and the original images that were used to assemble the figure.

We realize that it is difficult to revise to a specific deadline. In the interest of protecting the conceptual advance provided by the work, we recommend a revision within 3 months (24th Jun 2026). Please discuss the revision progress ahead of this time with the editor if you require more time to complete the revisions.

Referee #1:

Ulcerative colitis (UC) is a chronic relapsing inflammatory bowel disease characterized by mucosal inflammation, impaired intestinal barrier integrity, and increased colorectal cancer risk. Transient receptor potential vanilloid 3 (TRPV3), is a warm-sensitive Ca^{2+}-permeable ion channel, known for its role in skin barrier function, inflammation, itch, and pain, but its physiological and pathological roles in the gastrointestinal system remain poorly understood despite its abundant intestinal expression. While several TRPV3 inhibitors have been developed, most lack sufficient selectivity or oral bioavailability, limiting their systemic therapeutic potential.

In this study, the authors developed Colivin, a novel, selective, and orally bioavailable TRPV3 inhibitor derived from the compound KM-001. Cryo-EM structural analysis demonstrated that Colivin binds the TRPV3 vanilloid pocket and stabilizes non-conducting channel conformations, providing mechanistic insight into TRPV3 inhibition. Colivin significantly reduced intestinal inflammation in a dextran sulfate sodium (DSS)-induced mouse model of colitis in a TRPV3-dependent manner.

The work describing the inhibition of TRPV3 by Colivin is state of the art, comprehensive, and convincing. The pharmacological characterization is rigorous, and overall the study is well executed. I would not recommend major changes to this aspect of the manuscript.

The study is complete as presented. However, one major limitation is that there is currently no direct evidence demonstrating that TRPV3 activity in the intestinal epithelium specifically is responsible for the pathogenic effects observed, nor that reducing channel activity within epithelial cells themselves is sufficient to provide protection. Because TRPV3 is expressed in multiple cell types, including epithelial and non-epithelial compartments, clarification of the cellular locus of action would strengthen the mechanistic conclusions.

At a minimum, the authors could assess intestinal barrier permeability, as this would help determine whether TRPV3 inhibition prevents epithelial barrier disruption, a key pathogenic feature in many inflammatory gut models. Such data would provide functional support for an epithelial mechanism.

In addition, while the authors employ a global KO model, the inclusion of an epithelium-specific conditional knockout (e.g., Villin-Cre-mediated deletion) would substantially strengthen their interpretation. Given the strong pharmacological component already presented, it would be helpful for the authors to comment on whether incorporation of a conditional KO approach could be achieved within a reasonable experimental timeframe, or alternatively discuss this limitation explicitly.

Another important unresolved question concerns what endogenous signals activate TRPV3 in the disease model. Identifying or discussing potential activating mechanisms (e.g., inflammatory mediators, lipid metabolites, or tissue stress signals) would help place the findings into the context of a colitis model and improve mechanistic clarity. At minimum, this point warrants further discussion.

Minor comment:
Figure 4F is not referenced in the text. Please clarify its purpose. If the H&E staining is intended to illustrate tissue damage and/or immune cell infiltration, this should be explicitly stated in the Results section and figure legend.

Referee #2:

General summary and opinion
In this study, the authors developed a synthetic inhibitor of TRPV3, Colivin, using KM-001 as the molecular structural scaffold. They employed functional assays like cellular calcium imaging and whole cell patch-clamp to evaluate the selectivity and percent inhibition of the molecule. The authors also investigated the structural mechanisms underlying TRPV3 inhibition. Finally, the therapeutic effect of the drug was assessed using a DSS-induced colitis model in both wild-type and TRPV3 knockout mice. Overall, the experiments were carefully designed and conducted with appropriate controls. The study provides a comprehensive understanding of the compound, from its molecular mechanism to its physiological effects in vivo. However, I would like to suggest a few additional experiments that could further strengthen the reported results.

Major Concerns
1.In Figure 1B and C, the application of drugs at 60s appears to have some effect on the basal intracellular calcium level. If the molecule is plasma membrane permeable (as is typically the case for small molecule drugs), it may enter the cell and exert non-specific effects on intracellular proteins that could influence baseline calcium levels. As a control, I suggest that the authors measure the effect of Colivin on basal intracellular calcium levels in untransfected cells. If a reduction in baseline calcium is observed, determining the IC50 in untransfected cells and comparing it with the IC50 measured in TRPV3-transfected cells would help distinguish the TRPV3 specific inhibitory effect from potential non-specific effects of the compound.

2.In the subsequent structural experiments, the authors report that the molecule binds to the vanilloid pocket, with no density observed in the pore region. This suggests that the compound does not act as a pore blocker but instead inhibits the channel through negative allosteric modulation or by stabilizing an inactive state. In this context, I suggest the authors compare the EC50 and Emax of 2APB induced activation in the presence and absence of Colivin to measure the maximum level of inhibition the drug can achieve.

3.In continuation of the previous comment, since the structural data suggest that the molecule does not function as a pore blocker, it may not be necessary for the channel to be open before applying the drug in the patch clamp experiments. I suggest that the authors perform an experiment in which the channel is first activated by 2APB, followed by application of Colivin for a defined period (for example-1min) to allow potential channel inactivation, and then another 2APB activation to determine the extent of inhibition. Additionally, measuring the recovery of channel activity after washout of the drug would provide information about the reversibility and recovery kinetics of the channel.

4.In Figure 3, I suggest that the authors compare the structures of TRPV3-colivin1 and TRPV3-colivin2 with the apo closed state as well as with previously published closed and inactivated TRPV3 structures (for example- 8V6K and 8V6M). Comparing key structural features like the width of the upper selectivity filter, the rotation of cytoplasmic skirt and pore domain, the length and orientation of TRP helix, S6 helix, and height of the channel with known closed and inactivated states could help determine whether the drug bound structures more closely resemble a closed or inactivated conformation. This analysis would provide additional insight into whether the drug stabilizes the closed state, the inactivated state, or a distinct intermediate conformation.

5.Molecules that bind in the vanilloid pocket often act as agonists or potentiators of TRPV3. Did the authors check if Colivin has any activating or potentiating effect on the channel? This could be addressed using the experiments suggested above in comment#3.

Minor Concerns
1.In Figure EV1, the black and blue traces used in calcium imaging recordings are difficult to visually distinguish, particularly in the labels. I suggest modifying the color scheme.

2.The composition of the buffer used in the calcium imaging experiments should be mentioned in the Methods section. Is the same vehicle solution used in the mouse experiments also used as control in calcium imaging and patch-clamp experiments? If so, please include in the Methods.

Non-essential suggestions (at the author's/editor's discretion)
1.TRPV3 knockout significantly reduces DSS-induced colitis. It would be interesting to know whether Colivin also modulates

TRPV3 expression levels in mouse tissues by measuring TRPV3 mRNA levels following Colivin treatment.

Referee #3:

This is a very interesting and comprehensive manuscript describing the derivatization of a relatively high affinity inhibitor of TRPV3 to enhance oral availability and stability in vivo for treatment of inflammatory bowel disease. The activity of the new inhibitor named Colivin was carefully characterized using calcium flux assays, whole-cell patch clamp recordings, cryo-EM structures of apo hTRPV3 and with Colivin bound, mutagenesis and then in vivo using a model of ulcerative colitis in both WT mice and those lacking TRPV3. Overall, the authors have done a wonderful job of executing a beautiful study that provides definitive mechanistic insight and promise for the treatment of rehabilitating human diseases. Although I do not have sufficient expertise to critique the in vivo component of the study, I was impressed with how carefully the other parts of the study were done and also impressed with the use of both WT and TRPV3 KO mice to implicate TRPV3 in the model of ulcerative colitis and to establish the efficacy of their new inhibitor. I have only minor suggestions for improving an already very strong manuscript.

1) The cryo-EM density for Colivin in Fig EV 3 looks quite reasonable and it's also shown in Fig. 2A,B, but it's easy to miss as the surface is quite transparent. I suggest making the density for Colivin more pronounced and then also including cryo-EM density for the side chains of interacting residues so the reader can really appreciate the quality of the maps where the inhibitor binds.

2) It would be good for the authors to include data on the stability of KM-001 and Colivin for both human and mouse liver microsomes since this is a key component of the study. The results are mentioned in the text, but I could not find any of the data in the figures or appendix.

3) Perhaps I missed it but I could not find the data comparing the inhibitory activity of Colivin for mouse TRPV3, which is an important foundation for all the in vivo experiments.

4) I appreciate that the role of TRPV3 in the intestine is in its early days, but I think the discussion could be improved if the authors were to add a paragraph about how they think TRPV3 might be mechanistically involved in inflammatory bowel disease. The authors mention TRPV3 expression in the intestine, but it's also expressed in the immune system and this could well be where Colivin is acting. I understand that this added discussion will be speculative, but there is so much strong data in this manuscript that I think the authors have latitude to lay out possibilities for future studies to explore in search for an underlying mechanism.

5) 5-ASA is not defined when it is first mentioned on line 239 and why it is a positive control is never stated and should be.

6) In the discussion on line 339 the authors mention studies from Sobolevsky where they show lipids binding in the vanilloid binding pocket of TRPV3, but it would be also good to cite the related work by Cheng and Julius on TRPV1 [W. R. Arnold, A. Mancino, F. R. Moss, 3rd, A. Frost, D. Julius, Y. Cheng, Structural basis of TRPV1 modulation by endogenous bioactive lipids. Nat Struct Mol Biol, (2024); K. Zhang, D. Julius, Y. Cheng, Structural snapshots of TRPV1 reveal mechanism of polymodal functionality. Cell 184, 5138-5150 e5112 (2021); Y. Gao, E. Cao, D. Julius, Y. Cheng, TRPV1 structures in nanodiscs reveal mechanisms of ligand and lipid action. Nature 534, 347-351 (2016).]

EMBOJ-2025-123133R1

Point-by-point response to reviewers

To our reviewers:

Thank you very much for taking the time to review our manuscript "***Mechanism of Colivin Suppression of TRPV3 to Ameliorate Ulcerative Colitis***". In the following, we hope to address all concerns to your satisfaction. For your convenience, our answers are ***highlighted in red***.

With best regards,

Cao and colleagues.

Reviewers' Comments to Author:

Referee #1:

Ulcerative colitis (UC) is a chronic relapsing inflammatory bowel disease characterized by mucosal inflammation, impaired intestinal barrier integrity, and increased colorectal cancer risk. Transient receptor potential vanilloid 3 (TRPV3), is a warm-sensitive Ca^{2+}-permeable ion channel, known for its role in skin barrier function, inflammation, itch, and pain, but its physiological and pathological roles in the gastrointestinal system remain poorly understood despite its abundant intestinal expression. While several TRPV3 inhibitors have been developed, most lack sufficient selectivity or oral bioavailability, limiting their systemic therapeutic potential.

In this study, the authors developed Colivin, a novel, selective, and orally bioavailable TRPV3 inhibitor derived from the compound KM-001. Cryo-EM structural analysis demonstrated that Colivin binds the TRPV3 vanilloid pocket and stabilizes non-conducting channel conformations, providing mechanistic insight into TRPV3 inhibition. Colivin significantly reduced intestinal inflammation in a dextran sulfate sodium (DSS)-induced mouse model of colitis in a TRPV3-dependent manner.

The work describing the inhibition of TRPV3 by Colivin is state of the art,

comprehensive, and convincing. The pharmacological characterization is rigorous, and overall the study is well executed. I would not recommend major changes to this aspect of the manuscript.

Response: Thank you for your positive and insightful comments. In response to your concerns, we have conducted additional experiments and integrated the findings into the revised manuscript.

(1) The study is complete as presented. However, one major limitation is that there is currently no direct evidence demonstrating that TRPV3 activity in the intestinal epithelium specifically is responsible for the pathogenic effects observed, nor that reducing channel activity within epithelial cells themselves is sufficient to provide protection. Because TRPV3 is expressed in multiple cell types, including epithelial and non-epithelial compartments, clarification of the cellular locus of action would strengthen the mechanistic conclusions.

Response: Thank you for your constructive and insightful comments. We fully agree that defining the cell-type-specific role of TRPV3 in regulating ulcerative colitis (UC) is critical for further understanding the molecular mechanism. To address your concerns, we first performed immunofluorescence staining to analyze TRPV3 expression in colon tissues. As shown in **Figure R1** (also shown in **Appendix Fig. 3**), TRPV3 is expressed in colon tissues, predominantly in CK-18^{+} intestinal epithelial cells (IECs). Second, we are currently breeding a mouse line with IEC-specific deletion of TRPV3 (*Trpv3*$^{\Delta IEC}$) by crossing *Trpv3*$^{flox/flox}$ (*Trpv3*$^{f/f}$) mice with Villin-Cre mice. We have obtained preliminary data indicating that IEC-specific deletion of TRPV3 attenuates body weight loss, the severity of disease activity index (DAI), pathological scores, and the mRNA levels of inflammatory mediators such as TNF-α, IL-1β, CXCL-1, and IL-6 in response to DSS treatment, compared to the control, *Trpv3*$^{f/f}$ mice (**Figure R2**). These data are encouraging for an IEC-specific role of TRPV3 in UC. However, given the small sample size (n = 1-2), we consider the data preliminary and not ready for publication.

As indicated in our manuscript, although TRPV3 is abundantly expressed in intestinal tissues, its function in the intestinal system has not been thoroughly

investigated. Our current study demonstrates, for the first time, that either global deletion or pharmacological suppression of TRPV3 confers protective effects against UC. In addition, we uncover the molecular mechanism by which Colivin suppresses TRPV3 function. These major findings are of significant value to the field and need to be disseminated without delay. In the meantime, we will continue to complete the data collection from the *Trpv3*$^{\Delta IEC}$ mice, as well as additional negative controls, for example, conditional *Trpv3* knockout in other intestinal cell types, to solidify the cell-type specificity of TRPV3 in UC. We will also explore how TRPV3 is activated under inflammatory conditions and how such activation disrupts the IEC barrier. These interesting questions will take some time to address and are outside the scope of the current study.

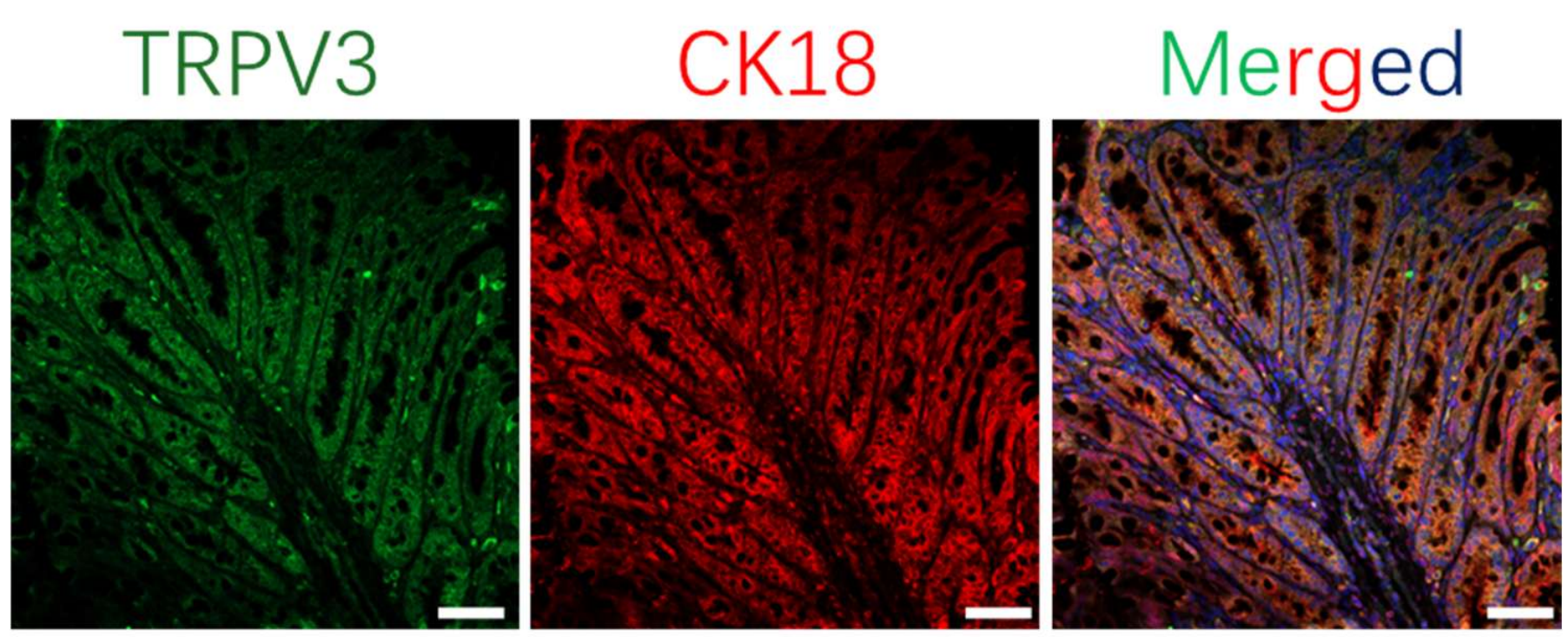

Figure R1. Representative immunofluorescent images of colon tissues stained with TRPV3 (*green*) and cytokeratin 18 (CK18, *red*). Nuclei were stained by Hoechst 33342 (*blue*). Scale bar = 50 µm.

Figure for referee with unpublished data and its description has been removed upon request by the authors.

(2) At a minimum, the authors could assess intestinal barrier permeability, as this would help determine whether TRPV3 inhibition prevents epithelial barrier disruption, a key pathogenic feature in many inflammatory gut models. Such data would provide functional support for an epithelial mechanism.

Response: Thank you for your insightful and constructive comments. In the revised

manuscript, we performed an additional experiment to validate the role of TRPV3 in regulating intestinal barrier permeability by measuring serum FITC fluorescence following oral administration of FITC-dextran (4 kDa, 400 mg/kg) for 4 h (Lee et al., 2015). As shown in **Figure R3**, DSS treatment for 6 consecutive days significantly increased serum FITC-dextran levels from 6.32 ± 0.49 to 20.24 ± 3.78 μg/mL ($P < 0.01$). Treatment with Colivin (10 mg/kg) significantly decreased serum FITC-dextran levels by 63.3 ± 16.2% ($P < 0.05$), supporting the view that Colivin can suppress DSS-induced barrier disruption. In DSS-treated *Trpv3*$^{-/-}$ mice, the serum level of FITC-dextran was 9.13 ± 1.63 μg/mL, representing a 79.8 ± 5.2% reduction ($P < 0.01$) compared to that in DSS-treated wild-type mice. Furthermore, administering Colivin (10 mg/kg) to *Trpv3*$^{-/-}$ mice did not further reduce serum FITC-dextran levels, indicating that the protective effect of Colivin on intestinal barrier integrity is solely dependent on TRPV3. These data have been incorporated into **Figure 5G**, with corresponding changes made in the main text (Page 10, line 285-293).

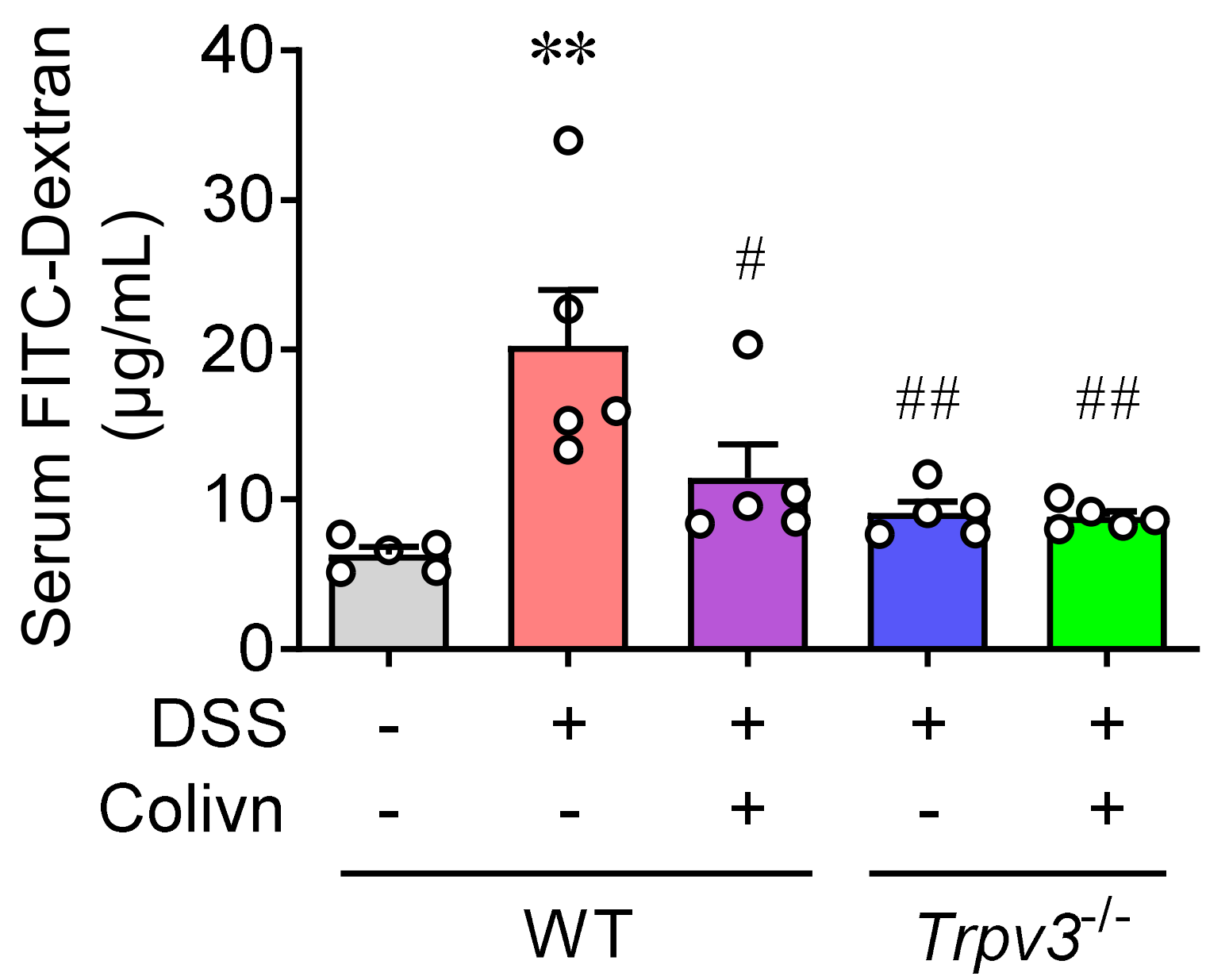

Figure R3. Effects of pharmacological suppression of TRPV3 by Colivin or genetical deletion of *Trpv3* on intestinal barrier permeability. Data represent mean ± SEM (N = 5 mice); **, $P < 0.01$, *vs.* Veh-treated WT mice; #, $P < 0.05$, ##, $P < 0.01$, *vs.* DSS-treated WT mice, by One-way ANOVA with Bonferroni's multiple comparisons tests.

To further demonstrate that TRPV3 inhibition prevents epithelial barrier disruption, we examined the expression of occludin and E-cadherin, two key proteins that regulate the tight junctions and adherent junctions of the intestinal epithelial barrier. As shown in **Figure R4** (also in **Figure 4H&I**), DSS exposure reduced the protein levels of occludin and E-cadherin by 55.2 ± 4.8% ($P < 0.01$) and 65.3 ± 7.8% ($P < 0.01$), respectively. Treatment with Colivin dose-dependently attenuated these DSS-induced reductions. At 10 mg/kg, Colivin significantly mitigated the DSS-induced reductions in occludin and E-cadherin expression by 86.4 ± 23.5% ($P < 0.01$) and 79.3 ± 8.6% ($P < 0.01$), respectively, with efficacy comparable to, if not better than, the positive control, 5-ASA (200 mg/kg; **Figure R4**). Consistent with the pharmacological inhibition, genetic deletion of *Trpv3* also significantly attenuated the DSS-induced reductions in occludin and E-cadherin protein levels, by 68.6 ± 9.8% ($P < 0.01$) and 78.8 ± 13.2% ($P < 0.01$), respectively (**Figure R5**). In DSS-treated *Trpv3*$^{-/-}$ mice, Colivin had no further effect on the expression levels of occludin and E-cadherin (**Figure R5**). Thus, both pharmacological inhibition and genetic ablation of TRPV3 prevent epithelial barrier disruption. These new data have been incorporated into **Figure 5H&I**. Corresponding changes have been made in the main text Page 9, line 257-262 and Page 10, line 289-293, respectively.

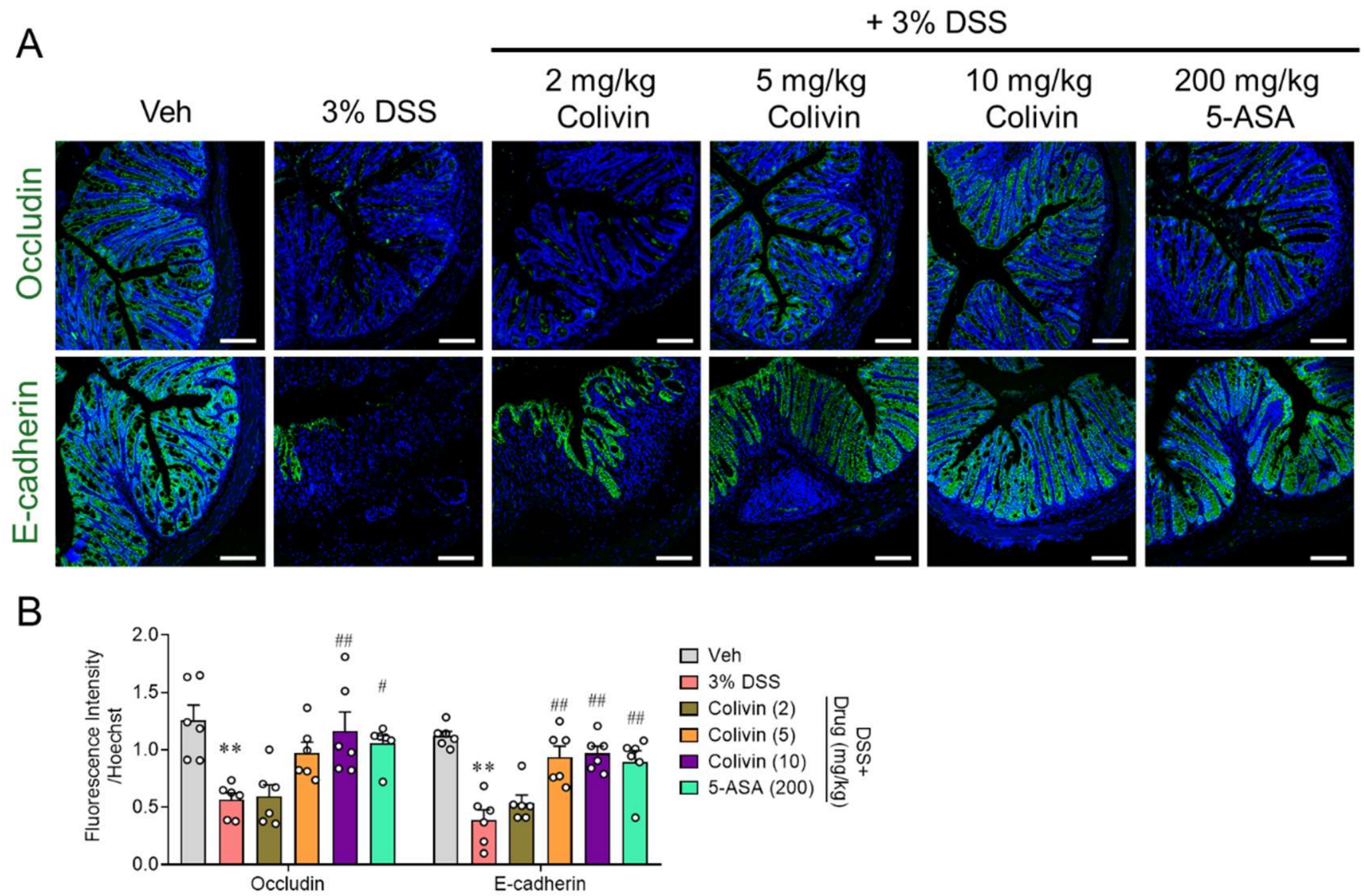

Figure R4. Colivin dose-dependently mitigated DSS-induced reductions in the protein expression of occludin and E-cadherin

(A) Representative immunofluorescent images of colon tissues stained for occludin (*top panel*) and E-cadherin (*bottom panel*) from mice exposed to 3% DSS for 6 days and treated with vehicle, different doses of Colivin, or the positive control 5-ASA (200 mg/kg). Nuclei were stained by Hoechst 33342 (*blue*). Scale bar = 100 µm. **(B)** Quantification of occludin and E-cadherin expression levels in different groups. Data represent mean ± SEM (N = 6); **, $P < 0.01$, *vs.* Veh; $^{\#}$, $P < 0.05$, $^{\#\#}$, $P < 0.01$, *vs.* DSS alone, by One-way ANOVA followed by Bonferroni's multiple comparisons tests.

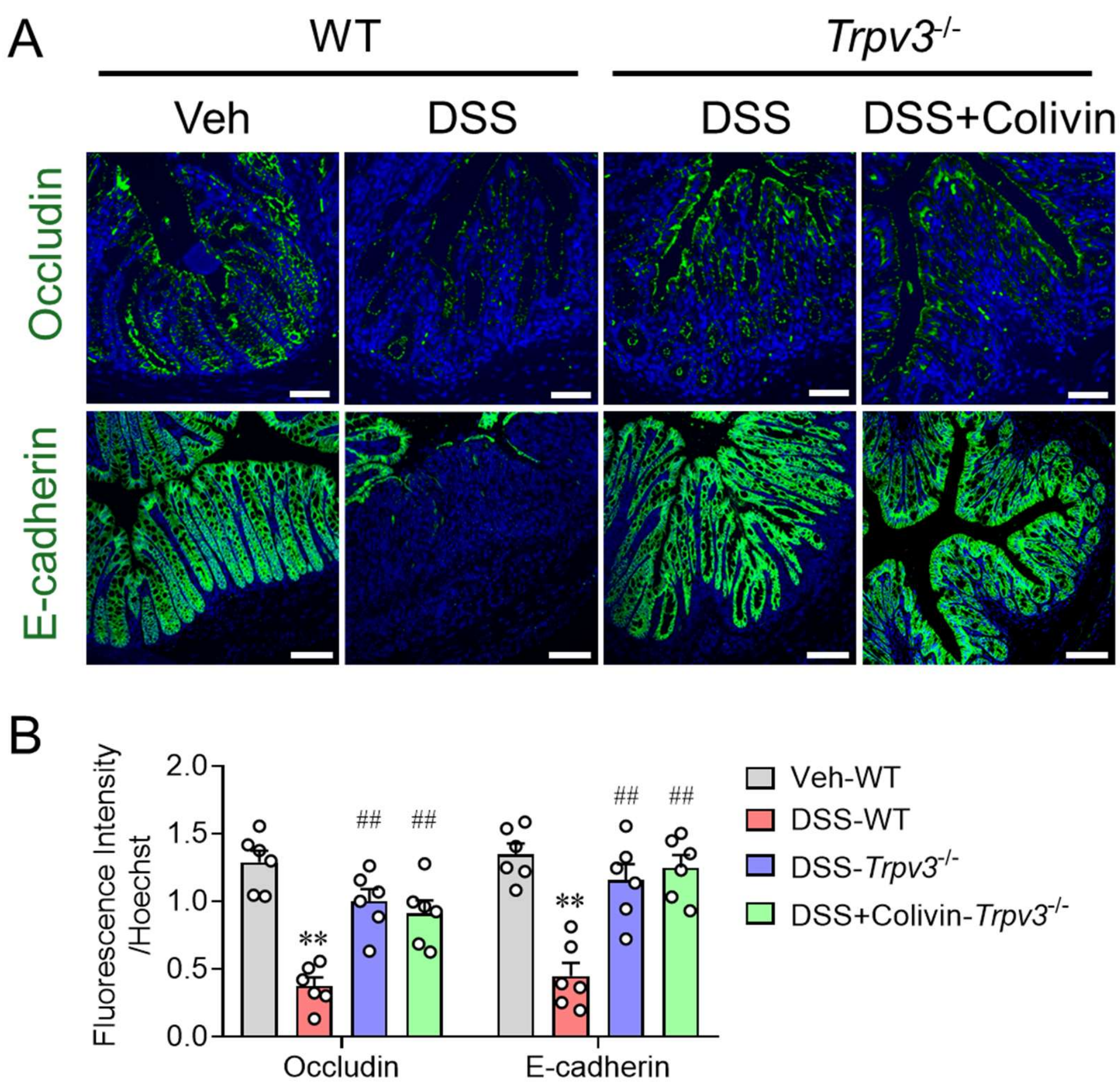

Figure R5. Colivin ameliorated DSS-induced reductions of occludin and E-cadherin expression through TRPV3

(**A**) Representative immunofluorescent images of colon tissues stained for occludin (*top panel*) and E-cadherin (*bottom panel*) from WT and *Trpv3*$^{-/-}$ mice exposed to DSS for 6 days and treated with vehicle or Colivin (10 mg/kg). Nuclei were stained by Hoechst 33342 (*blue*). Scale bar = 50 µm. **(B)** Quantification of occludin and E-cadherin expression levels in different groups. Data represent mean ± SEM. N = 6; **, $P < 0.01$, *vs.* Veh-WT; $^{\#\#}$, $P < 0.01$, *vs.* DSS-WT, by One-way ANOVA with Bonferroni's multiple comparisons tests.

(3) In addition, while the authors employ a global KO model, the inclusion of an epithelium-specific conditional knockout (e.g., Villin-Cre-mediated deletion) would substantially strengthen their interpretation. Given the strong pharmacological component already presented, it would be helpful for the authors to comment on

whether incorporation of a conditional KO approach could be achieved within a reasonable experimental timeframe, or alternatively discuss this limitation explicitly.

Response: Thank you for the valuable and insightful comments. As mentioned above (**Response to comment 1**), we fully agree that defining the cell-type-specific role of TRPV3 in regulating ulcerative colitis (UC) is critical for further understanding the molecular mechanism. We have obtained preliminary data employing mice with IEC-specific TRPV3 deletion (*Trpv3*$^{\Delta IEC}$) that IEC-expressed TRPV3 is a major contributor to UC (**Figure R2**). However, we need to increase the sample size and perform more thorough analyses of other cell types as well. Despite the predominance in IECs, TRPV3 expression has also been reported in other intestinal cell types, such as sensory neurons and immune cells (Çakır et al., 2021, McGaraughty et al., 2017, Niu et al., 2025), both of which have been shown to regulate UC progression (Engel et al., 2012, Ungaro et al., 2017). Therefore, more cell-type-specific knockout mice are required to provide a comprehensive understanding of how TRPV3 in different intestinal cell types contributes to UC progression. Additional studies are also needed to unveil the molecular and signaling mechanisms involved. We have incorporated these points in the Discussion Section, including the limitations of the current study (Page 12, line 345-355).

(4) Another important unresolved question concerns what endogenous signals activate TRPV3 in the disease model. Identifying or discussing potential activating mechanisms (e.g., inflammatory mediators, lipid metabolites, or tissue stress signals) would help place the findings into the context of a colitis model and improve mechanistic clarity. At minimum, this point warrants further discussion.

Response: Thank you for your valuable and insightful comments. The following sentences have been incorporated into the Discussion Section.

Moreover, as a polymodal sensor, TRPV3 responds not only to exogenous natural and synthetic compounds such as camphor, carvacrol, and 2-APB (Su et al., 2023) but also to endogenous environmental factors, including arachidonic acid derivatives that are elevated in the inflamed colonic mucosa of UC patients (Hu et al., 2006, Nishida et

al., 1987) and isopentenyl pyrophosphate, a metabolic alarmin (Bang et al., 2010, Ji et al., 2026). TRPV3 channel activity can also be potentiated by phospholipase C (PLC)-dependent release from PIP_2 inhibition, as well as by ERK-dependent phosphorylation of Thr^{264} in the N-terminal ankyrin repeat domain (Doerner et al., 2011, Vyklicka et al., 2017), two signaling pathways that are often activated by inflammatory mediators such as IL-1β and TNF-α (Amin et al., 2003, Eliopoulos et al., 2003). Future studies are needed to determine the endogenous signals responsible for TRPV3 activation under inflammatory conditions such as UC (Page 12, line 355-361 and Page 13, line 362-367).

(5) Minor comment:

Figure 4F is not referenced in the text. Please clarify its purpose. If the H&E staining is intended to illustrate tissue damage and/or immune cell infiltration, this should be explicitly stated in the Results section and figure legend.

Response: Thank you. In the original manuscript, we wrote "DSS administration also led to colon atrophy and histopathologic changes, most notably crypt loss and lamina propria edema accompanied by inflammatory infiltration, which were suppressed by Colivin administration (**Fig. 4D-G**)". To avoid the confusion, we have changed the sentence to "DSS administration also led to colon atrophy and histopathologic changes, most notably crypt loss and lamina propria edema accompanied by inflammatory infiltration, which were suppressed by Colivin administration (**Fig. 4D, E, F, G**)" (Page 9, Line 250-253).

Referee #2:

General summary and opinion

In this study, the authors developed a synthetic inhibitor of TRPV3, Colivin, using KM-001 as the molecular structural scaffold. They employed functional assays like cellular calcium imaging and whole cell patch-clamp to evaluate the selectivity and percent inhibition of the molecule. The authors also investigated the structural mechanisms underlying TRPV3 inhibition. Finally, the therapeutic effect of the drug was assessed using a DSS-induced colitis model in both wild-type and TRPV3 knockout mice. Overall, the experiments were carefully designed and conducted with appropriate controls. The study provides a comprehensive understanding of the compound, from its molecular mechanism to its physiological effects *in vivo*.

However, I would like to suggest a few additional experiments that could further strengthen the reported results.

Response: Thank you for your positive comments. We have addressed your concerns as follows.

Major Concerns

1. In Figure 1B and C, the application of drugs at 60s appears to have some effect on the basal intracellular calcium level. If the molecule is plasma membrane permeable (as is typically the case for small molecule drugs), it may enter the cell and exert non-specific effects on intracellular proteins that could influence baseline calcium levels. As a control, I suggest that the authors measure the effect of Colivin on basal intracellular calcium levels in untransfected cells. If a reduction in baseline calcium is observed, determining the IC_{50} in untransfected cells and comparing it with the IC_{50} measured in TRPV3-transfected cells would help distinguish the TRPV3 specific inhibitory effect from potential non-specific effects of the compound.

Response: Thank you for raising this point. The baseline change is likely an artifact created by fluid transfer, as it is also seen with the addition of vehicle (0.1% DMSO) (**Figure 1B**, Blue line) or the addition of the same buffer in which cells were maintained (not shown). This is very typical with plate-reader-based assays (in our case, $FLIPR^{tetra}$), in which the treatment solution is added directly to the well without washing or removing the pre-existing solution.

2. In the subsequent structural experiments, the authors report that the molecule binds to the vanilloid pocket, with no density observed in the pore region. This suggests that the compound does not act as a pore blocker but instead inhibits the channel through negative allosteric modulation or by stabilizing an inactive state. In this context, I suggest the authors compare the EC_{50} and E_{max} of 2APB induced activation in the presence and absence of Colivin to measure the maximum level of inhibition the drug can achieve.

Response: Thank you for your valuable comments. To address this concern, we performed whole-cell voltage-clamp recordings on HEK293 cells expressing hTRPV3. Cells were first treated with 1 mM 2-APB to elicit maximal TRPV3 currents. After washing, increasing concentrations of 2-APB were applied in the presence or absence of 1 μM Colivin. Co-application of Colivin caused a slight

rightward shift in the EC_{50} value of 2-APB (67.5 ± 13.4 μM without Colivin *vs.* 104.5 ± 19.2 μM with Colivin), while strongly suppressing the maximal current amplitude (**Figure R6**, also shown in **Fig. 1K, L, M**). These results align with structural evidence, supporting the idea that Colivin acts as a non-competitive negative allosteric modulator of TRPV3. Corresponding changes have been made in the Results section (Page 6, line 140-143).

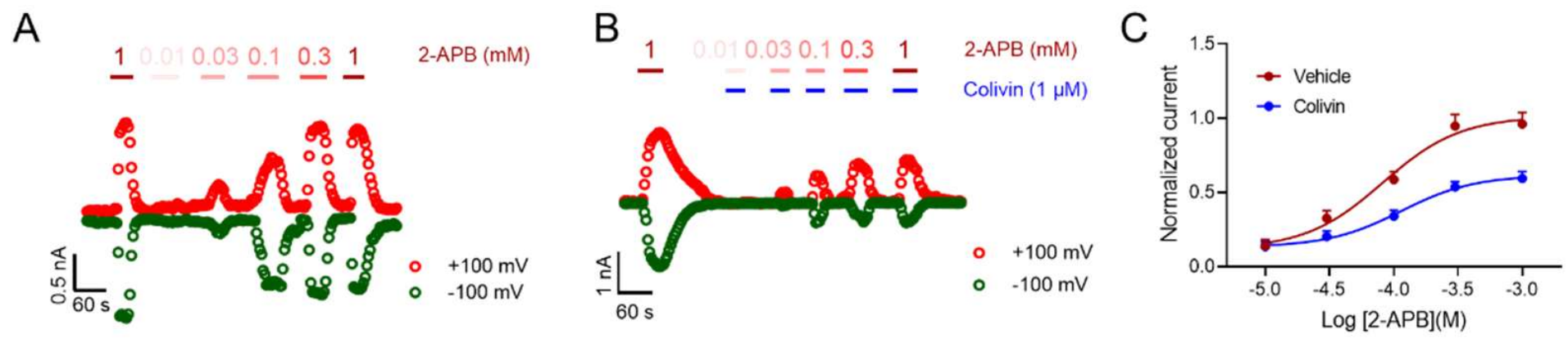

Figure R6. Colivin acts as a non-competitive inhibitor of TRPV3

(A-B) Whole-cell hTRPV3 currents at ±100 mV evoked by the 2-APB (0.01-1 mM) in the absence (**A**) and presence (**B**) of Colivin (1 μM). **(C)** Concentration-response curves of 2-APB-induced hTRPV3 currents at +100 mV in the absence and presence of Colivin. The currents were normalized to the maximal current evoked by 1 mM 2-APB in the absence of Colivin for each cell. Each data point represents mean ± SEM (N = 5 cells).

3. In continuation of the previous comment, since the structural data suggest that the molecule does not function as a pore blocker, it may not be necessary for the channel to be open before applying the drug in the patch clamp experiments. I suggest that the authors perform an experiment in which the channel is first activated by 2-APB, followed by application of Colivin for a defined period (for example-1min) to allow potential channel inactivation, and then another 2-APB activation to determine the extent of inhibition. Additionally, measuring the recovery of channel activity after washout of the drug would provide information about the reversibility and recovery kinetics of the channel.

Response: Thank you for your valuable comment. As shown in **Figure R7** (also in **Fig. 1N&O**), application of 2-APB (100 μM) rapidly evoked robust currents in hTRPV3-expressing HEK-293 cells. After washout, cells were treated with Colivin (1 μM) for ~2 min. Although this treatment did not alter basal hTRPV3 currents, it did suppress 2-

APB response by 71.3 ± 14.1% and 77.0 ± 9.3% (+100 mV and -100 mV, respectively), suggesting that Colivin does not rely on channel activation to exert an inhibitory effect on hTRPV3. Following an extended washout period, the response to 2-APB recovered to 77.3 ± 29.3% and 64.2 ± 20.6% (+100 mV and -100 mV, respectively) of the initial current amplitudes, indicating that the inhibition by Colivin is reversible. Corresponding changes have been made in the Results section (Page 6, line 143-150).

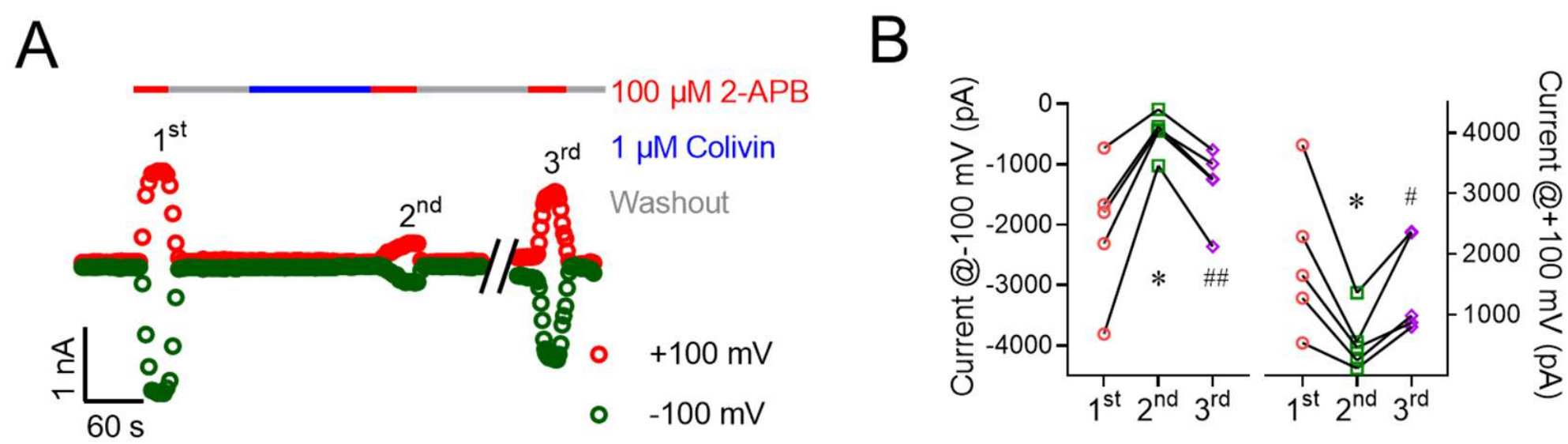

Figure R7. Colivin suppression of TRPV3 currents does not require TRPV3 activation and is reversible

(A) Whole-cell hTRPV3 currents at ±100 mV recorded during sequential perfusion with 2-APB (100 µM) and Colivin (1 µM), as indicated. **(B)** Quantification of the current amplitude evoked by sequential applications of 2-APB. Each connected line (*black*) represents a data set from the same cell (N = 5 cells). *, $P < 0.05$, *vs.* first application of 2-APB; #, $P < 0.05$, ##, $P < 0.01$, *vs.* second application of 2-APB, by One-way ANOVA with Bonferroni's multiple comparisons tests.

4. In Figure 3, I suggest that the authors compare the structures of $TRPV3_{colivin1}$ and $TRPV3_{colivin2}$ with the apo closed state as well as with previously published closed and inactivated TRPV3 structures (for example- 8V6K and 8V6M). Comparing key structural features like the width of the upper selectivity filter, the rotation of cytoplasmic skirt and pore domain, the length and orientation of TRP helix, S6 helix, and height of the channel with known closed and inactivated states could help determine whether the drug bound structures more closely resemble a closed or inactivated conformation. This analysis would provide additional insight into whether the drug stabilizes the closed state, the inactivated state, or a distinct intermediate conformation.

Response: Thank you for your valuable comment. In response, we have conducted a structural comparison of $TRPV3_{Colivin\text{-}1}$ and $TRPV3_{Colivin\text{-}2}$ with the apo closed state, as well as with previously published closed (PDB ID: 8V6K) and inactivated (PDB ID: 8V6M) TRPV3 structures (**Figure R8**, also in **Appendix Fig. 2**). For comparison, we also calculated the pore radii along the permeation pathways of the closed and inactivated TRPV3 structures (**Figure R9**, also in **Figure 2J**). Corresponding changes have been incorporated into the Results section (Page 7, line 201-202 and Page 8, line 203-232)

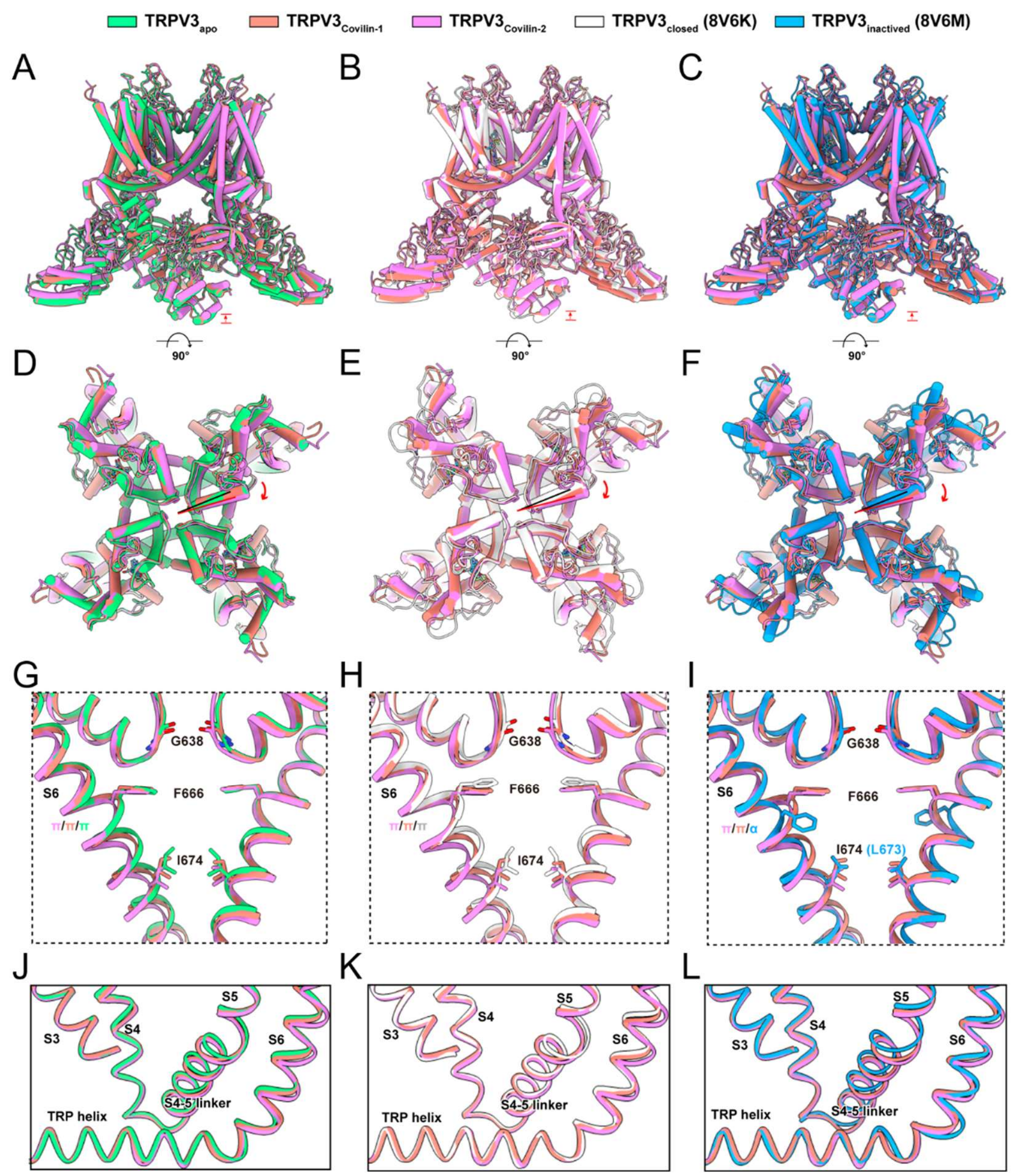

Figure R8. Structural comparison of Colivin-bound TRPV3 with closed and inactivated TRPV3 states

(A-C) Side views of structural overlays of TRPV3$_{Colivin-1}$ (light salmon) and TRPV3$_{Colivin-2}$ (violet) with TRPV3$_{apo}$ (spring green) (**A**), closed TRPV3 (white; PDB ID: 8V6K) (**B**), and inactivated TRPV3 (blue; PDB ID: 8V6M) (**C**). Red arrows indicate conformational shifts observed in TRPV3$_{Colivin-2}$.

(D-F) Top-down views of the same structural overlays. Red arrows indicate conformational shifts observed in TRPV3$_{Colivin-2}$.

(G-I) Boxed regions show pore-forming domains, with residues contributing to pore lining shown as sticks. Only two of the four subunits are shown, with the front and back subunits omitted for clarity.

(J-L) Boxed regions show the transmembrane domains.

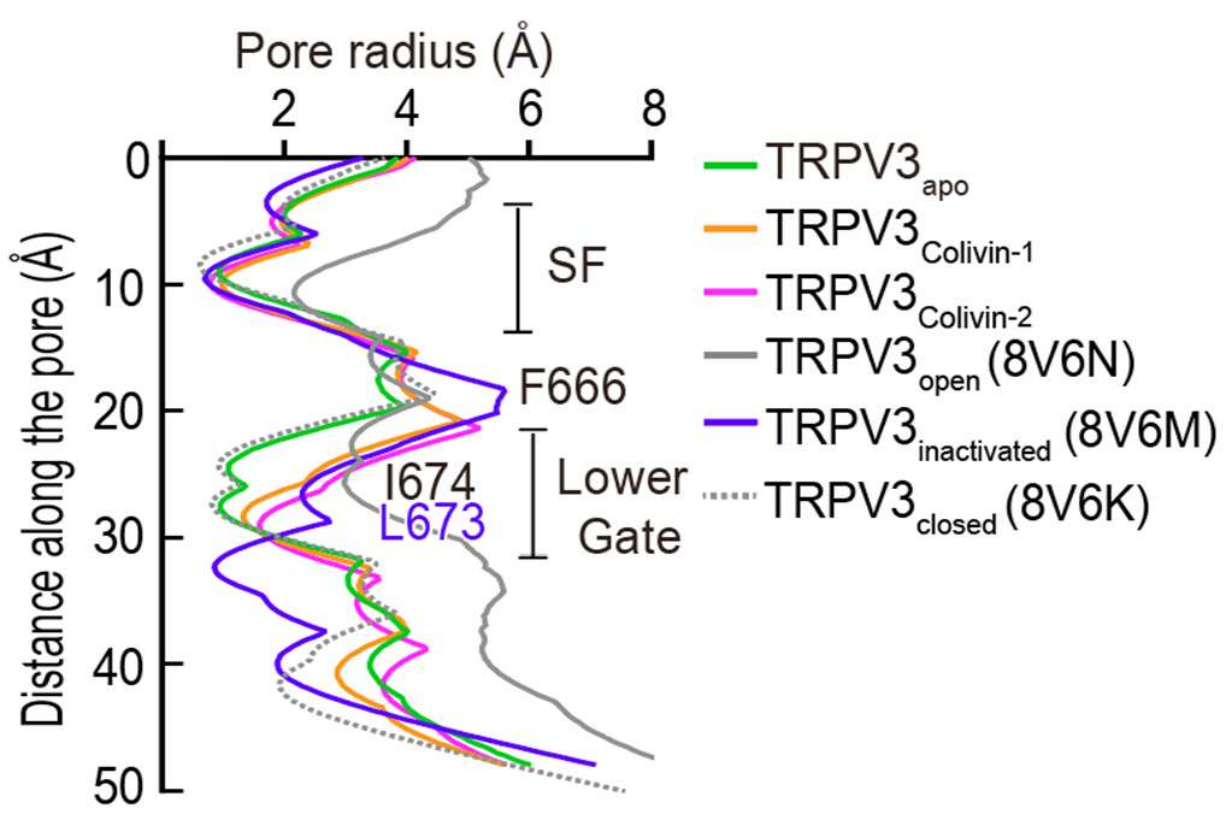

Figure R9. Pore radii along the permeation pathways of TRPV3$_{apo}$, TRPV3$_{Colivin-1}$, TRPV3$_{Colivin-2}$, TRPV3$_{closed}$ (PDB ID: 8V6K), TRPV3$_{inactivated}$ (PDB ID: 8V6M) and TRPV3$_{open}$ (PDB ID: 8V6N), calculated with HOLE. Two constriction sites of the selectivity filters and the activation gates are shown as SF and Lower Gate.

5. Molecules that bind in the vanilloid pocket often act as agonists or potentiators of TRPV3. Did the authors check if Colivin has any activating or potentiating effect on the channel? This could be addressed using the experiments suggested above in comment#3.

Response: Thank you for your insightful comment. As shown in **Figure R7A**, we did not observe any inhibitory or activating effect of Colivin on TRPV3 currents in the absence of 2-APB.

Minor Concerns

1.In Figure EV1, the black and blue traces used in calcium imaging recordings are difficult to visually distinguish, particularly in the labels. I suggest modifying the color scheme.

Response: Thank you for the comment. To facilitate distinction, we have changed the colors of the Fluo-4 fluorescence traces (**Figure R10**). The original Figure EV1 has been replaced in the revised manuscript.

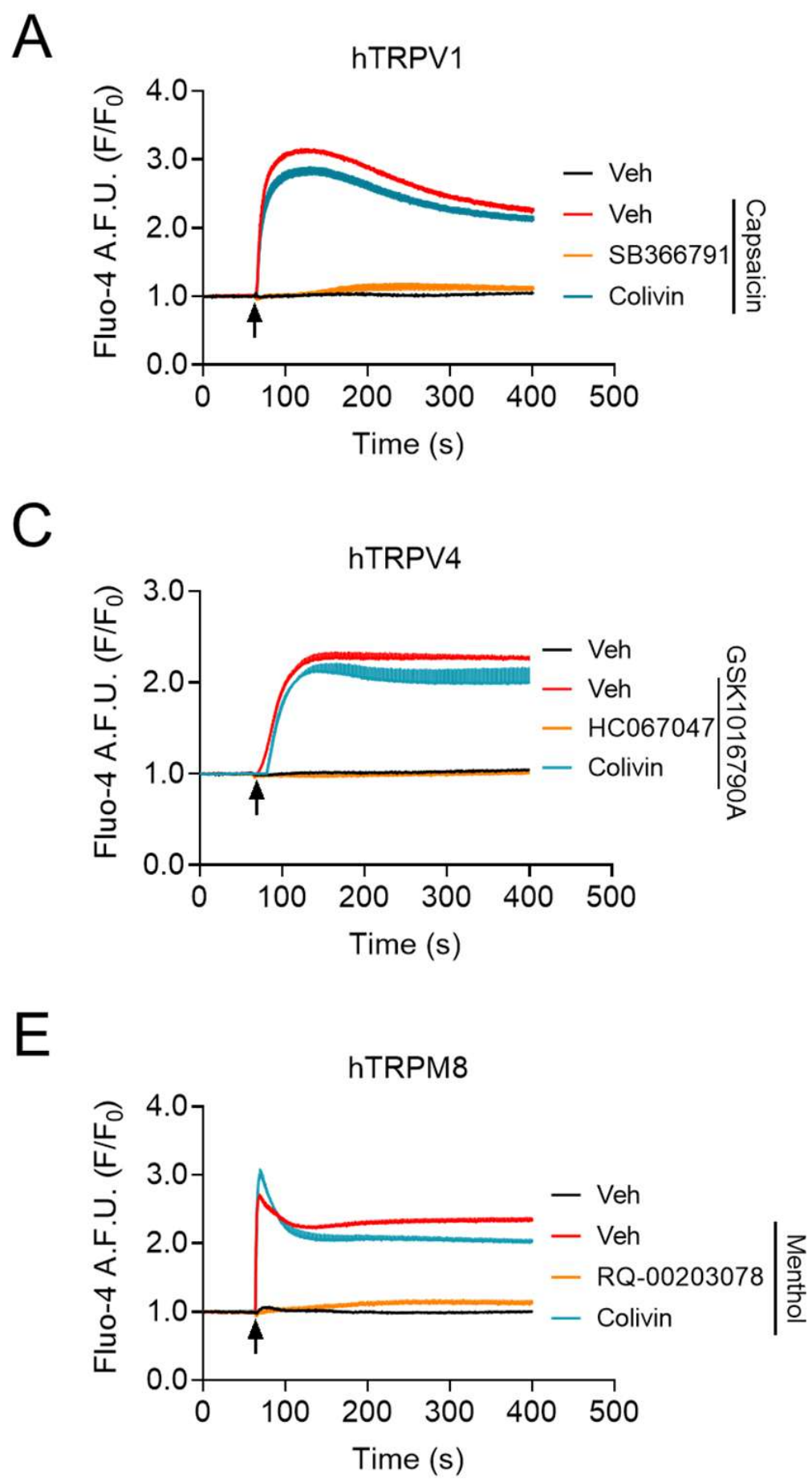

Figure R10. Effects of Colivin on thermosensitive TRP channels.

Time courses of Ca^{2+} influx induced by capsaicin (1 μM), 2-APB (100 μM), GSK1016790A (10 nM), AITC (30 μM), and menthol (300 μM) in HEK-293 cells expressing hTRPV1 (**A**), mTRPV2 (**B**), hTRPV4 (**C**), hTRPA1 (**D**), and hTRPM8 (**E**), respectively. Colivin (10 μM), along with the corresponding inhibitors: SB366791 (1 μM), ruthenium red (30 μM), HC067047 (1 μM), A-967079 (1 μM), and RQ-00203078 (1 μM), was incubated for 5 mins, followed by the addition of agonists at 60 seconds (*black arrow*). Each data point represents Mean ± SEM (N = 3 wells). Each experiment was repeated in three independent cultures with similar results.

2.The composition of the buffer used in the calcium imaging experiments should be mentioned in the Methods section. Is the same vehicle solution used in the mouse experiments also used as control in calcium imaging and patch-clamp experiments? If so, please include in the Methods.

Response: Thank you for the valuable suggestions. The buffer compositions used in the calcium imaging experiments is not the same solution used in patch-clamp and animal study. We have included buffer compositions used in the calcium imaging experiments into the revised manuscript (Page 16, line 465-468).

Non-essential suggestions (at the author's/editor's discretion)

1. TRPV3 knockout significantly reduces DSS-induced colitis. It would be interesting to know whether Colivin also modulates TRPV3 expression levels in mouse tissues by measuring TRPV3 mRNA levels following Colivin treatment.

Response: Thank you for the insightful comment. To address this, we quantified *Trpv3* mRNA levels by RT-qPCR in colon tissues from mice treated with vehicle (Veh), 3% DSS, or 3% DSS plus 10 mg/kg Colivin. As shown in **Figure R11**, DSS treatment induced a 2.96 ± 0.19-fold increase in *Trpv3* mRNA expression. Notably, application of Colivin (10 mg/kg) attenuated the DSS-induced upregulation of *Trpv3* mRNA by 86.4 ± 14.9% ($P < 0.01$).

Figure for referee with unpublished data and its description has been removed upon request by the authors.

Referee #3:

This is a very interesting and comprehensive manuscript describing the derivatization of a relatively high affinity inhibitor of TRPV3 to enhance oral availability and stability *in vivo* for treatment of inflammatory bowel disease. The activity of the new inhibitor

named Colivin was carefully characterized using calcium flux assays, whole-cell patch clamp recordings, cryo-EM structures of apo hTRPV3 and with Colivin bound, mutagenesis and then *in vivo* using a model of ulcerative colitis in both WT mice and those lacking TRPV3. Overall, the authors have done a wonderful job of executing a beautiful study that provides definitive mechanistic insight and promise for the treatment of rehabilitating human diseases. Although I do not have sufficient expertise to critique the *in vivo* component of the study, I was impressed with how carefully the other parts of the study were done and also impressed with the use of both WT and TRPV3 KO mice to implicate TRPV3 in the model of ulcerative colitis and to establish the efficacy of their new inhibitor. I have only minor suggestions for improving an already very strong manuscript.

Response: Thank you for the positive comments.

1) The cryo-EM density for Colivin in Fig. EV 3 looks quite reasonable and it's also shown in **Fig. 2A, B**, but it's easy to miss as the surface is quite transparent. I suggest making the density for Colivin more pronounced and then also including cryo-EM density for the side chains of interacting residues so the reader can really appreciate the quality of the maps where the inhibitor binds.

Response: We appreciate this insightful comment. In response, we have optimized the visualization of the Colivin cryo-EM density map in the revised figures to enhance its clarity. (**Figure 2A and Figure EV3**). Specifically, the density is now displayed in mesh. Furthermore, as suggested, we have now included the cryo-EM density for the key side chains of residues involved in inhibitor binding, as shown in **Figure R12**.

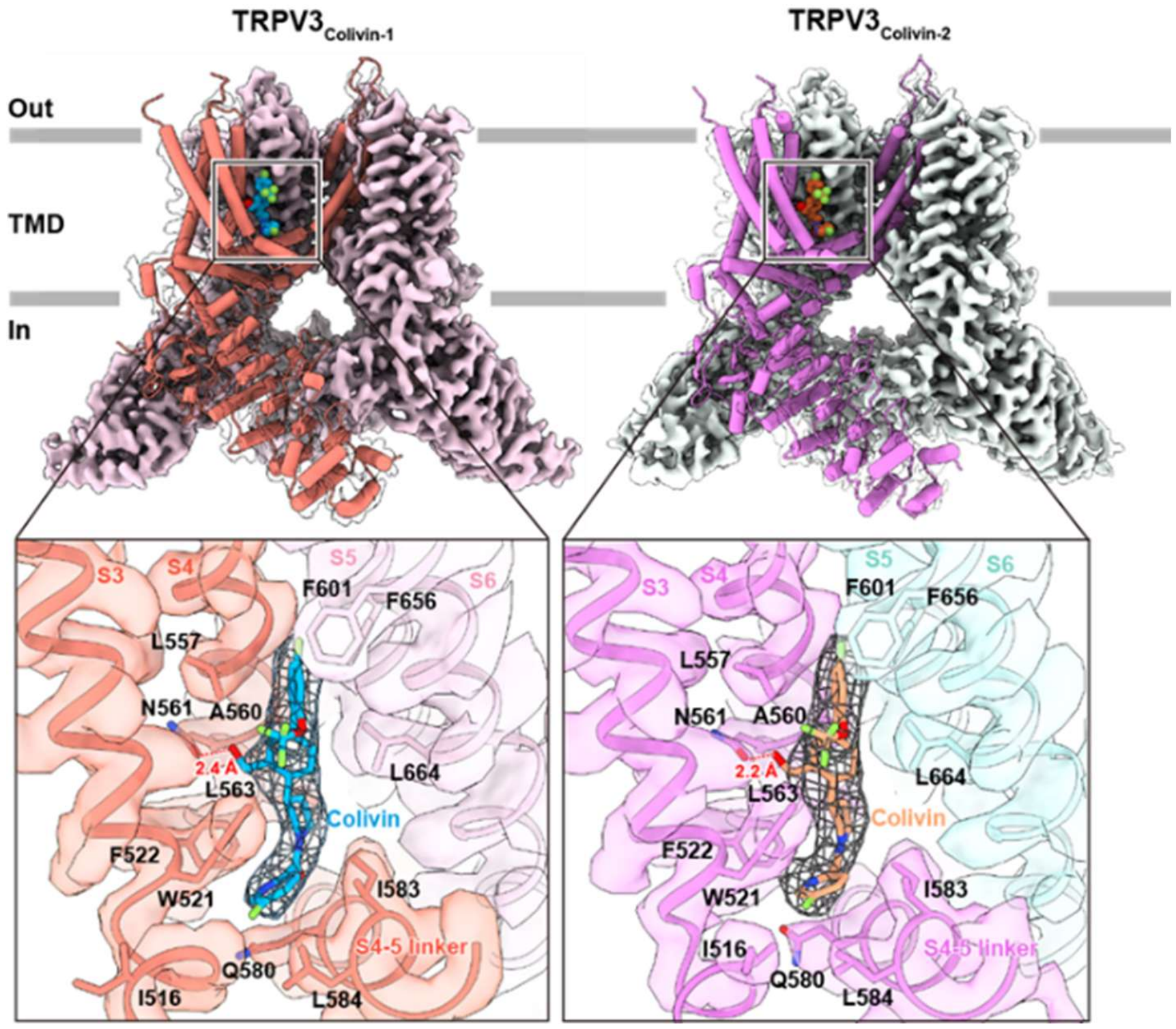

Figure R12. Structures of TRPV3$_{Colivin-1}$ (deep skyblue) and TRPV3$_{Colivin-2}$ (wheat) tetramers determined by single-particle cryo-EM, with the Colivin-binding sites enlarged in the boxes. One subunit is shown in light salmon or violet cartoon, with a transparent surface, while the other three are displayed in a density map colored in pink or cyan, contoured at 0.25. Cryo-EM densities for Colivin are shown as mesh in the expanded boxes. The hydrogen bonds between the carboxyl group of Asn^{561} and the hydroxyl group of Colivin are shown as red dashed lines, with distances marked below.

2) It would be good for the authors to include data on the stability of KM-001 and Colivin for both human and mouse liver microsomes since this is a key component of the study. The results are mentioned in the text, but I could not find any of the data in the figures or appendix.

Response: Thank you for your valuable suggestion. The liver microsome stability data of KM-001 and Colivin for both human and mouse liver microsomes have now been incorporated into the revised manuscript as **Table 1** (also provided here as **Table R1** for your convenience).

Table R1. The metabolic profile of KM001 and Colivin in mouse and human liver microsomes

Test Compound	Species	$T_{1/2}$ (min)	Cl_{Hep} (mL/min/kg)
KM001	Mouse	2.24	86.79
	Human	12.5	1343
Colivin	Mouse	12.77	74.34
	Human	70.8	903

Hepatic clearance: $Cl_{Hep} = HBF \times Cl / (HBF + Cl)$

Clearance: $Cl = 0.693 \times V / T_{1/2} / BW$

HBF: Hepatic blood flow; V: The incubation volume; BW: Body weight.

3) Perhaps I missed it but I could not find the data comparing the inhibitory activity of Colivin for mouse TRPV3, which is an important foundation for all the in vivo experiments.

Response: Thank you. The inhibition of mTRPV3 by Colivin is in the **Figure 1H, I, J** in the original manuscript.

4) I appreciate that the role of TRPV3 in the intestine is in its early days, but I think the discussion could be improved if the authors were to add a paragraph about how they think TRPV3 might be mechanistically involved in inflammatory bowel disease. The authors mention TRPV3 expression in the intestine, but it's also expressed in the immune system and this could well be where Colivin is acting. I understand that this added discussion will be speculative, but there is so much strong data in this manuscript that I think the authors have latitude to lay out possibilities for future studies to explore in search for an underlying mechanism.

Response: Thank you for the valuable and insightful suggestion. Please see our response to Reviewer 1, comments 1 & 3.

A paragraph has been added to discuss the limitations and future direction as follows:

In the current study, we demonstrate that TRPV3 is predominantly expressed in $CK18^+$

IECs and that inhibiting TRPV3, either by Colivin or gene ablation, alleviates DSS-induced IEC barrier disruption. It is thus tempting to conclude that IEC-expressed TRPV3 serves as a major contributor to UC by disrupting the epithelial barrier. However, TRPV3 has also been reported to be expressed in other cell types, such as sensory neurons and immune cells (Çakır et al., 2021, McGaraughty et al., 2017, Niu et al., 2025), both of which have been shown to regulate UC progression (Engel et al., 2012, Ungaro et al., 2017). Therefore, cell-type-specific *Trpv3* knockout mice should be tested in order to gain a comprehensive understanding of how TRPV3 in different intestinal cell types contributes to UC progression. Additional studies are also needed to unveil the molecular and signaling mechanisms involved. Moreover, as a polymodal sensor, TRPV3 responds not only to exogenous natural and synthetic compounds such as camphor, carvacrol, and 2-APB (Su et al., 2023) but also to endogenous environmental factors, including arachidonic acid derivatives that are elevated in the inflamed colonic mucosa of UC patients (Hu et al., 2006, Nishida et al., 1987) and isopentenyl pyrophosphate, a metabolic alarmin (Bang et al., 2010, Ji et al., 2026). TRPV3 can also be potentiated by phospholipase C (PLC)-dependent release from PIP_2 inhibition, as well as by ERK-dependent phosphorylation of Thr^{264} in the N-terminal ankyrin repeat domain (Doerner et al., 2011, Vyklicka et al., 2017), two signaling pathways that are often activated by inflammatory mediators such as IL-1β and TNF-α (Amin et al., 2003, Eliopoulos et al., 2003). Future studies are needed to determine the endogenous signals responsible for TRPV3 activation under inflammatory conditions such as UC (Page 12, line 345-361 and Page 13, line 362-367).

5) 5-ASA is not defined when it is first mentioned on line 239 and why it is a positive control is never stated and should be.

Response: Thank you for your concern. 5-Aminosalicylic Acid (5-ASA) is a clinically approved drug for UC and is widely used as a positive control in animal studies (Xue et al., 2023). This information has been incorporated into the revised manuscript (Page 10, line 270-271).

6) In the discussion on line 339 the authors mention studies from Sobolevsky where they show lipids binding in the vanilloid binding pocket of TRPV3, but it would be also good to cite the related work by Cheng and Julius on TRPV1 [W. R. Arnold, A. Mancino, F. R. Moss, 3rd, A. Frost, D. Julius, Y. Cheng, Structural basis of TRPV1 modulation

by endogenous bioactive lipids. Nat Struct Mol Biol, (2024); K. Zhang, D. Julius, Y. Cheng, Structural snapshots of TRPV1 reveal mechanism of polymodal functionality. Cell 184, 5138-5150 e5112 (2021); Y. Gao, E. Cao, D. Julius, Y. Cheng, TRPV1 structures in nanodiscs reveal mechanisms of ligand and lipid action. Nature 534, 347-351 (2016).]

Response: We thank the reviewer for this valuable suggestion. We agree that citing the related work by Cheng, Julius, and colleagues on TRPV1-lipid interactions would provide important context and strengthen our discussion. Accordingly, we have now cited these three papers in the revised manuscript. A sentence "Similarly, structural work has revealed that phosphatidylinositol lipids stabilize the closed state by occupying the vanilloid pocket of TRPV1 (Arnold et al., 2024, Gao et al., 2016, Zhang et al., 2021)" has been added to the discussion section (Page 14, line 405-407).

References

1. Amin AR, Ichigotani Y, Oo ML, Biswas MH, Yuan H, Huang P, Mon NN, Hamaguchi M (2003) The PLC-PKC cascade is required for IL-1beta-dependent Erk and Akt activation: their role in proliferation. *Int J Oncol* 23: 1727-31
2. Bang S, Yoo S, Yang TJ, Cho H, Hwang SW (2010) Farnesyl pyrophosphate is a novel pain-producing molecule via specific activation of TRPV3. *J Biol Chem* 285: 19362-71
3. Çakır M, Saçmacı H, Sabah-Özcan S (2021) Selected transient receptor potential channel genes' expression in peripheral blood mononuclear cells of multiple sclerosis. *Hum Exp Toxicol* 40: S406-s413
4. Doerner JF, Hatt H, Ramsey IS (2011) Voltage- and temperature-dependent activation of TRPV3 channels is potentiated by receptor-mediated PI(4,5)P2 hydrolysis. *J Gen Physiol* 137: 271-88
5. Eliopoulos AG, Wang CC, Dumitru CD, Tsichlis PN (2003) Tpl2 transduces CD40 and TNF signals that activate ERK and regulates IgE induction by CD40. *EMBO J* 22: 3855-64
6. Engel MA, Khalil M, Mueller-Tribbensee SM, Becker C, Neuhuber WL, Neurath MF, Reeh PW (2012) The proximodistal aggravation of colitis depends on substance P released from TRPV1-expressing sensory neurons. *J Gastroenterol* 47: 256-65
7. Hu HZ, Xiao R, Wang C, Gao N, Colton CK, Wood JD, Zhu MX (2006) Potentiation of TRPV3 channel function by unsaturated fatty acids. *J Cell Physiol* 208: 201-12

8. Ji Z, Gao J, Zhang S, Li J, Wu H, Yao J, Ma X, Xin Y, Zhu Y, Zhao M, Zhao Z, Shen K, Wu T, Qian X, Wang J, An H, Li Y, Sun W, Zhao Q, Zhou X et al. (2026) A metabolic alarmin from keratinocytes potentiates systemic humoral immunity. *Nature* 652: 209-219
9. Lee JS, Tato CM, Joyce-Shaikh B, Gulen MF, Cayatte C, Chen Y, Blumenschein WM, Judo M, Ayanoglu G, McClanahan TK, Li X, Cua DJ (2015) Interleukin-23-Independent IL-17 Production Regulates Intestinal Epithelial Permeability. *Immunity* 43: 727-38
10. McGaraughty S, Chu KL, Xu J, Leys L, Radek RJ, Dart MJ, Gomtsyan A, Schmidt RG, Kym PR, Brederson JD (2017) TRPV3 modulates nociceptive signaling through peripheral and supraspinal sites in rats. *J Neurophysiol* 118: 904-916
11. Nishida T, Miwa H, Shigematsu A, Yamamoto M, Iida M, Fujishima M (1987) Increased arachidonic acid composition of phospholipids in colonic mucosa from patients with active ulcerative colitis. *Gut* 28: 1002-7
12. Niu X, Guo A, Liu X, Li H, Ji H, Wang C (2025) A novel TRP channel-related prognostic model of glioma based on transcriptomics and single cell sequencing analysis. *Discov Oncol* 17: 29
13. Su W, Qiao X, Wang W, He S, Liang K, Hong X (2023) TRPV3: Structure, Diseases and Modulators. *Molecules* 28: 774
14. Ungaro R, Mehandru S, Allen PB, Peyrin-Biroulet L, Colombel JF (2017) Ulcerative colitis. *Lancet* 389: 1756-1770
15. Vyklicka L, Boukalova S, Macikova L, Chvojka S, Vlachova V (2017) The human transient receptor potential vanilloid 3 channel is sensitized via the ERK pathway. *J Biol Chem* 292: 21083-21091
16. Xue C, Zhang X, Ge H, Tang Q, Jeon J, Zhao F, Wang Y, Zhu MX, Cao Z (2023) Total flavone of flowers of *Abelmoschus manihot* (L.) Medic inhibits the expression of adhesion molecules in primary mesenteric arterial endothelial cells and ameliorates dextran sodium sulphate-induced ulcerative colitis in mice. *Phytomedicine* 112: 154713

Dear Prof. Cao,

We have now received re-review reports from three referees, which I have included below. As you will see, you have addressed their concerns satisfactorily. Before I can finally accept the manuscript, there are some remaining editorial points which need to be addressed. In this regard would you please:

- include an ORCID for Jie Yu,
- rename the 'Conflict of interest' statement to the "Disclosure and Competing Interests Statement",
- acknowledge employment in a biotech company in this Disclosure and Competing Interests Statement,
- for long author lists in the reference section, use the first ten authors followed by 'et al.',
- rename the "Data and materials availability" section as "Data Availability",
- remove the AC/CrediT section from the text,
- ensure all figure callouts are listed sequentially in the manuscript main text, and include callouts for panels for EV figures and Appendix Figures S4-S7,
- upload the appendix file in PDF format; the title page should contain "Appendix for + Mechanism of Colivin Suppression of TRPV3 to Ameliorate Ulcerative Colitis" (author and affiliation list not necessary) and a table of contents with the page numbers for the listed items; here, the nomenclature should be Appendix Figure Sx and Appendix Table Sx throughout ms and Appendix PDF,
- upload the synopsis image at exactly 550 pixels wide and 300 pixels high, ensuring that the text is legible at the final size and that the synopsis image has appropriate proportions,
- provide suggestions for a short 'blurb' text prefacing and summing up the conceptual aspect of the study in two sentences (max. 250 characters), followed by 3-5 one-sentence 'bullet points' with brief factual statements of key results of the paper; they will form the basis of an editor-written 'Synopsis' accompanying the online version of the article,
- ensure datasets EMD-66282, EMD-66283 and EMD-66284, 9WV5, 9WV6 and 9WV7 datasets are publicly available,
- provide exact p values in the legends of figures 2D, 3D, 4B, C, E, G, I, J, K; 5A, B, D, F, G, I, J, K,
- define n in the legend of figure 4B,
- define error bars in the legends of figures 1D, G, J; 2D, E; 4B,
- rename "Discussion and Conclusion" as "Discussion",
- rename "Materials and Methods" as "Methods", and

use the following section order: Title page - Abstract - Introduction - Results - Discussion - Methods - Data Availability - Acknowledgements - Disclosure and Competing Interests Statement - References - Figure Legends - Table(s) - Expanded View Figure Legends.

I am looking forward to receiving your revised manuscript.

EMBO Press is an editorially independent publishing platform for the development of EMBO scientific publications.

Best wishes,

William

William Teale, PhD
Editor
The EMBO Journal
w.teale@embojournal.org

Instructions for preparing your revised manuscript:

Read our guidance for manuscript revisions and related editorial policies: https://link.springer.com/journal/44318/submission-guidelines#cms-Revised-submissions

Please also check that the title and abstract of the manuscript are brief, yet explicit, even to non-specialists.

When assembling figures, please refer to our figure preparation guideline in order to ensure proper formatting and readability in print as well as on screen:
https://media.springernature.com/original/springer-cms/rest/v1/content/27825798/data/v1

IMPORTANT: When you send the revision we will require
- a point-by-point response to the referees' comments, with a detailed description of the changes made (as a word file).

- a word file of the manuscript text.
- individual production quality figure files (one file per figure)
- a complete author checklist
- Expanded View files (replacing Supplementary Information)
- a Reagents and Tools Table as part of the Methods section

Please remember: Digital image enhancement is acceptable practice, as long as it accurately represents the original data and conforms to community standards. If a figure has been subjected to significant electronic manipulation, this must be noted in the figure legend or in the 'Methods' section. The editors reserve the right to request original versions of figures and the original images that were used to assemble the figure.

We realize that it is difficult to revise to a specific deadline. In the interest of protecting the conceptual advance provided by the work, we recommend a revision within 3 months (4th Oct 2026). Please discuss the revision progress ahead of this time with the editor if you require more time to complete the revisions. :

Referee #1:

The authors have addressed my concerns. I believe the manuscript is now suitable for publication.

Referee #2:

All major and minor concerns have been addressed. The newly added electrophysiological experiments clearly demonstrate that Colivin acts as an allosteric, non-competitive inhibitor. The authors have provided satisfactory responses to my comments.

Referee #3:

As stated in my original review, I think this is an outstanding paper. The authors have been very responsive to all of the reviewers' suggestions, and in my view, the study is now ready for publication. I think that this study is one of the most convincing and compelling studies I have reviewed in the past few years.

EMBOJ-2025-123133R1

Point-by-point response to editors

To editors:

Thank you very much for handling our manuscript “***Mechanism of Colivin Suppression of TRPV3 to Ameliorate Ulcerative Colitis***”. We have carefully addressed all editorial concerns point by point below, and hope our responses satisfy your requirements. For ease of reading, all responses are *highlighted in red.*

With best regards,

Cao and colleagues.

- include an ORCID for Jie Yu,

Response: The ORCID of Jie Yu is 0000-0001-9227-038X.

- rename the 'Conflict of interest' statement to the "Disclosure and Competing Interests Statement",

Response: The “Conflict of interest statement” in the original manuscript has been changed to "Disclosure and Competing Interests Statement" in the revised manuscript (page 23, line 678).

- acknowledge employment in a biotech company in this Disclosure and Competing Interests Statement,

Response: A statement has been added to the “Disclosure and Competing Interests Statement” (page 23, lines 679–681) as follows:

"Author Zhengyu Cao is the founder of Nanjing Poregen Biotechnology Co., Ltd. The company had no role in the study design, data collection, data analysis, manuscript preparation, or the decision to publish."

- for long author lists in the reference section, use the first ten authors followed by 'et al.',

Response: The author names in the reference list have been reformatted to comply

with the journal's reference style and formatting requirements.

- rename the "Data and materials availability" section as "Data Availability",

Response: The section title “Data and Materials Availability” has been revised to “Data Availability” in the revised manuscript (page 22, line 658) to comply with the journal's formatting requirements.

- remove the AC/CrediT section from the text,

Response: The “Author Contributions” section has been removed in the revised manuscript.

- ensure all figure callouts are listed sequentially in the manuscript main text, and include callouts for panels for EV figures and Appendix Figures S4-S7,

Response: We have carefully checked the manuscript to ensure that all figure callouts, including those for the EV Figures and Appendix Figures, appear in the correct sequential order throughout the main text.

- upload the appendix file in PDF format; the title page should contain "Appendix for + Mechanism of Colivin Suppression of TRPV3 to Ameliorate Ulcerative Colitis" (author and affiliation list not necessary) and a table of contents with the page numbers for the listed items; here, the nomenclature should be Appendix Figure Sx and Appendix Table Sx throughout ms and Appendix PDF,

Response: We have uploaded a revised Appendix file in PDF format that includes a detailed table of contents on the title page. In addition, the nomenclature of the Appendix Figures and Tables has been revised consistently throughout both the main manuscript and the Appendix, in accordance with the journal's requirements.

- upload the synopsis image at exactly 550 pixels wide and 300 pixels high, ensuring that the text is legible at the final size and that the synopsis image has appropriate proportions,

Response: We uploaded a synopsis image with resolution meets the requirements.

- provide suggestions for a short 'blurb' text prefacing and summing up the conceptual aspect of the study in two sentences (max. 250 characters), followed by 3-5 one-

sentence 'bullet points' with brief factual statements of key results of the paper; they will form the basis of an editor-written 'Synopsis' accompanying the online version of the article,

Response: A “Synopsis description” file containing a short blurb and bullet points has been uploaded as a Related Manuscript File

- ensure datasets EMD-66282, EMD-66283 and EMD-66284, 9WV5, 9WV6 and 9WV7 datasets are publicly available,

Response: The Cryo-EM structure datasets (EMD-66282, EMD-66283 and EMD-66284, 9WV5, 9WV6 and 9WV7) will be publicly accessible on July 15th.

- provide exact p values in the legends of figures 2D, 3D, 4B, C, E, G, I, J, K; 5A, B, D, F, G, I, J, K,

Response: Thank you. For the clarity of the figure legends, the exact p values in Figures 1O, 2D, 4B, C, E, G, I, J, K, and 5A, B, D, F, G, I, J, K have been listed in Dataset EV1. This file has been uploaded as a Related Manuscript File. We have also added the following statement to the figure legends: “Exact *P* values for all comparisons are listed in Dataset EV1.”

- define n in the legend of figure 4B,

Response: N = 6 mice has been added into the legend of Fig. 4B (page 32, line 941).

- define error bars in the legends of figures 1D, G, J; 2D, E; 4B,

Response: The error bars in the legends of Fig. 1D, G, J; 2D, E; 4B have been defined (page 30, line 868, 875-876, page 31, line 912 and page 32, line 916, 941).

- rename "Discussion and Conclusion" as "Discussion",

Response: The section title “Discussion and Conclusion” has been revised into “Discussion” (page 11, line 304).

- rename "Materials and Methods" as "Methods",

Response: The section title “Materials and Methods” has been revised into “Methods” (page 15, line 428).

and use the following section order: Title page - Abstract - Introduction - Results -

Discussion - Methods - Data Availability - Acknowledgements - Disclosure and Competing Interests Statement - References - Figure Legends - Table(s) - Expanded View Figure Legends.

Response: We have carefully checked the revised manuscript to ensure that it follows the required section order: Title Page, Abstract, Introduction, Results, Discussion, Methods, Data Availability, Acknowledgements, Disclosure and Competing Interests Statement, References, Figure Legends, Tables, and Expanded View Figure Legends.

2nd Revision - Editorial Decision 21st Jul 2026

Dear Zhengyu,

I am pleased to inform you that your manuscript has been accepted for publication in the EMBO Journal.

Congratulations to you and your team!

Your manuscript will be processed for publication by EMBO Press. It will be copy edited and you will receive page proofs prior to publication. Please note that you will be contacted by Springer Nature Author Services to complete licensing and payment information.

You may qualify for financial assistance for your publication charges - either via a Springer Nature fully open access agreement or an EMBO initiative. Check your eligibility: https://link.springer.com/journal/44318/how-to-publish-with-us

Should you be planning a Press Release on your article, please notify the production team via this form in order to coordinate publication and release dates: https://forms.cloud.microsoft/e/eH4A8JVP6g

If you have any questions, please do not hesitate to contact the Editorial Office. Thank you for your contribution to The EMBO Journal.

Best wishes,

William

William Teale, PhD
Editor
The EMBO Journal
w.teale@embojournal.org

*****
Please note that it is The EMBO Journal policy for the transcript of the editorial process (containing referee reports and your response letters) to be published as an online supplement to each paper. If you should prefer removal of any referee-only figures included in the point-by-point response(s), e.g. because they may still be used for future publication or because they have been reproduced from published work by others, please do let us know immediately via response email.
More information is available here: https://link.springer.com/partners/embo-press/editorial-policies#Peer%20review

EMBO Press Author Checklist

Corresponding Author Name: Zhengyu Cao, Jie Yu, Kai Wang	
Journal Submitted to: EMBO Journal	
Manuscript Number: EMBOJ-2025-123133R1	

USEFUL LINKS FOR COMPLETING THIS FORM
The EMBO Journal - Author Guidelines
EMBO Reports - Author Guidelines
Molecular Systems Biology - Author Guidelines
EMBO Molecular Medicine - Author Guidelines

Reporting Checklist for Life Science Articles (updated January
This checklist is adapted from Materials Design Analysis Reporting (MDAR) Checklist for Authors. MDAR establishes a minimum set of requirements in transparent reporting in the life sciences (see Statement of Task: 10.31222/osf.io/9sm4x). Please follow the journal's guidelines in preparing your
Please note that a copy of this checklist will be published alongside your article.

Abridged guidelines for figures
1. Data
The data shown in figures should satisfy the following conditions:
- the data were obtained and processed according to the field's best practice and are presented to reflect the results of the experiments in an accurate and unbiased manner.
- ideally, figure panels should include only measurements that are directly comparable to each other and obtained with the same assay.
- plots include clearly labeled error bars for independent experiments and sample sizes. Unless justified, error bars should not be shown for technical
- if n<5, the individual data points from each experiment should be plotted. Any statistical test employed should be justified.
- Source Data should be included to report the data underlying figures according to the guidelines set out in the authorship guidelines on Data

2. Captions
Each figure caption should contain the following information, for each panel where they are relevant:
- a specification of the experimental system investigated (eg cell line, species name).
- the assay(s) and method(s) used to carry out the reported observations and measurements.
- an explicit mention of the biological and chemical entity(ies) that are being measured.
- an explicit mention of the biological and chemical entity(ies) that are altered/varied/perturbed in a controlled manner.
- the exact sample size (n) for each experimental group/condition, given as a number, not a range;
- a description of the sample collection allowing the reader to understand whether the samples represent technical or biological replicates (including how many animals, litters, cultures, etc.).
- a statement of how many times the experiment shown was independently replicated in the laboratory.
- definitions of statistical methods and measures:
  - common tests, such as t-test (please specify whether paired vs. unpaired), simple χ2 tests, Wilcoxon and Mann-Whitney tests, can be unambiguously identified by name only, but more complex techniques should be described in the methods section;
  - are tests one-sided or two-sided?
  - are there adjustments for multiple comparisons?
  - exact statistical test results, e.g., P values = x but not P values < x;
  - definition of 'center values' as median or average;
  - definition of error bars as s.d. or s.e.m.

Please complete ALL of the questions below.
Select "Not Applicable" only when the requested information is not relevant for your study.

Materials

Newly Created Materials	Information included in the manuscript?	In which section is the information available? (Reagents and Tools Table, Materials and Methods, Figures, Data Availability Section)
New materials and reagents need to be available; do any restrictions apply?	Not Applicable	

Antibodies	Information included in the manuscript?	In which section is the information available? (Reagents and Tools Table, Materials and Methods, Figures, Data Availability Section)
For **antibodies** provide the following information:<br>- Commercial antibodies: RRID (if possible) or supplier name, catalogue number and or/clone number<br>- Non-commercial: RRID or citation	Yes	Materials and Methods

DNA and RNA sequences	Information included in the manuscript?	In which section is the information available? (Reagents and Tools Table, Materials and Methods, Figures, Data Availability Section)
**Short novel DNA or RNA including primers, probes**: provide the sequences.	Yes	Appendix

Cell materials	Information included in the manuscript?	In which section is the information available? (Reagents and Tools Table, Materials and Methods, Figures, Data Availability Section)
**Cell lines:** Provide species information, strain. Provide accession number in repository **OR** supplier name, catalog number, clone number, and/**OR** RRID.	Yes	Materials and Methods
**Primary cultures:** Provide species, strain, sex of origin, genetic modification status.	Not Applicable	
Report if the cell lines were recently **authenticated** (e.g., by STR profiling) and tested for mycoplasma contamination.	Not Applicable	

Experimental animals	Information included in the manuscript?	In which section is the information available? (Reagents and Tools Table, Materials and Methods, Figures, Data Availability Section)
**Laboratory animals or Model organisms:** Provide species, strain, sex, age, genetic modification status. Provide accession number in repository **OR** supplier name, catalog number, clone number, **OR** RRID.	Yes	Materials and Methods
**Animal observed in or captured from the field:** Provide species, sex, and age where possible.	Not Applicable	
Please detail **housing and husbandry conditions**.	Yes	Materials and Methods

Plants and microbes	Information included in the manuscript?	In which section is the information available? (Reagents and Tools Table, Materials and Methods, Figures, Data Availability Section)
**Plants:** provide species and strain, ecotype and cultivar where relevant, unique accession number if available, and source (including location for collected wild specimens).	Not Applicable	
**Microbes:** provide species and strain, unique accession number if available, and source.	Not Applicable	

Human research participants	Information included in the manuscript?	In which section is the information available? (Reagents and Tools Table, Materials and Methods, Figures, Data Availability Section)
If collected and within the bounds of privacy constraints report on age, sex and gender or ethnicity for all study participants.	Not Applicable	

Core facilities	Information included in the manuscript?	In which section is the information available? (Reagents and Tools Table, Materials and Methods, Figures, Data Availability Section)
If your work benefited from core facilities, was their service mentioned in the acknowledgments section?	Yes	Acknowledgments

Design

Study protocol	Information included in the manuscript?	In which section is the information available? (Reagents and Tools Table, Materials and Methods, Figures, Data Availability Section)
If study protocol has been **pre-registered, provide DOI in the manuscript**. For clinical trials, provide the trial registration number **OR** cite DOI.	Not Applicable	
Report the **clinical trial registration number** (at ClinicalTrials.gov or equivalent), where applicable.	Not Applicable	

Laboratory protocol	Information included in the manuscript?	In which section is the information available? (Reagents and Tools Table, Materials and Methods, Figures, Data Availability Section)
Provide DOI OR other citation details if **external detailed step-by-step protocols** are available.	Yes	Materials and Methods

Experimental study design and statistics	Information included in the manuscript?	In which section is the information available? (Reagents and Tools Table, Materials and Methods, Figures, Data Availability Section)
Include a statement about **sample size** estimate even if no statistical methods were used.	Yes	Materials and Methods
Were any steps taken to minimize the effects of subjective bias when allocating animals/samples to treatment (e.g. **randomization procedure**)? If yes, have they been described?	Yes	Materials and Methods
Include a statement about **blinding** even if no blinding was done.	Yes	Materials and Methods
Describe **inclusion/exclusion criteria** if samples or animals were excluded from the analysis. Were the criteria pre-established?<br><br>If sample or data points were omitted from analysis, report if this was due to attrition or intentional exclusion and provide justification.	Not Applicable	
For every figure, are **statistical tests** justified as appropriate? Do the data meet the assumptions of the tests (e.g., normal distribution)? Describe any methods used to assess it. Is there an estimate of variation within each group of data? Is the variance similar between the groups that are being statistically compared?	Yes	Materials and Methods

Sample definition and in-laboratory replication	Information included in the manuscript?	In which section is the information available? (Reagents and Tools Table, Materials and Methods, Figures, Data Availability Section)
In the figure legends: state number of times the experiment was **replicated** in laboratory.	Yes	Figure legends
In the figure legends: define whether data describe **technical or biological replicates**.	Yes	Figure legends

Ethics

Ethics	Information included in the manuscript?	In which section is the information available? (Reagents and Tools Table, Materials and Methods, Figures, Data Availability Section)
Studies involving **human participants**: State details of **authority granting ethics approval** (IRB or equivalent committee(s), provide reference number for approval.	Not Applicable	
Studies involving **human participants**: Include a statement confirming that **informed consent** was obtained from all subjects and that the experiments conformed to the principles set out in the WMA Declaration of Helsinki and the Department of Health and Human Services Belmont Report.	Not Applicable	
Studies involving **human participants:** For publication of **patient photos**, include a statement confirming that consent to publish was obtained.	Not Applicable	
Studies involving experimental **animals**: State details of **authority granting ethics approval** (IRB or equivalent committee(s), provide reference number for approval. Include a statement of compliance with ethical regulations.	Yes	Materials and Methods
Studies involving **specimen and field samples:** State if relevant **permits** obtained, provide details of authority approving study; if none were required, explain why.	Not Applicable	

Dual Use Research of Concern (DURC)	Information included in the manuscript?	In which section is the information available? (Reagents and Tools Table, Materials and Methods, Figures, Data Availability Section)
Could your study fall under dual use research restrictions? Please check biosecurity documents and list of **select agents and toxins** (CDC): https://www.selectagents.gov/sat/list.htm	Not Applicable	
If you used a select agent, is the security level of the lab appropriate and reported in the manuscript?	Not Applicable	
If a study is subject to dual use research of concern regulations, is the name of the **authority granting approval and reference number** for the regulatory approval provided in the manuscript?	Not Applicable	

Reporting

The MDAR framework recommends adoption of discipline-specific guidelines, established and endorsed through community initiatives. Journals have their own policy about requiring specific guidelines and recommendations to complement MDAR.

Adherence to community standards	Information included in the manuscript?	In which section is the information available? (Reagents and Tools Table, Materials and Methods, Figures, Data Availability Section)
State if relevant guidelines or checklists (e.g., **ICMJE, MIBBI, ARRIVE, PRISMA**) have been followed or provided.	Yes	Materials and Methods
For **tumor marker prognostic studies,** we recommend that you follow the **REMARK** reporting guidelines (see link list at top right). See author guidelines, under 'Reporting Guidelines'. Please confirm you have followed these guidelines.	Not Applicable	
For **phase II and III randomized controlled trials**, please refer to the **CONSORT** flow diagram (see link list at top right) and submit the CONSORT checklist (see link list at top right) with your submission. See author guidelines, under 'Reporting Guidelines'. Please confirm you have submitted this list.	Not Applicable	

Data Availability

Data availability	Information included in the manuscript?	In which section is the information available? (Reagents and Tools Table, Materials and Methods, Figures, Data Availability Section)
Have **primary datasets** been deposited according to the journal's guidelines (see 'Data Deposition' section) and the respective accession numbers provided in the Data Availability Section?	Yes	Data and materials availability
Were **human clinical and genomic datasets** deposited in a public access-controlled repository in accordance to ethical obligations to the patients and to the applicable consent agreement?	Not Applicable	
Are **computational models** that are central and integral to a study available without restrictions in a machine-readable form? Were the relevant accession numbers or links provided?	Not Applicable	
If publicly available data were reused, provide the respective **data citations in the reference list**.	Not Applicable	
