# Supplementary material for: Orally available Colivin traps TRPV3 in an inactive state and suppresses intestinal inflammation
Source: EMBO J. 2026 Aug 13;45(18):6480–500. doi: 10.1038/s44318-026-00896-9 (PMC13578699; doi:10.1038/s44318-026-00896-9)
Supplement: Supplementary file 3 [file 44318_2026_896_MOESM9_ESM.pdf]

Expanded View Figures

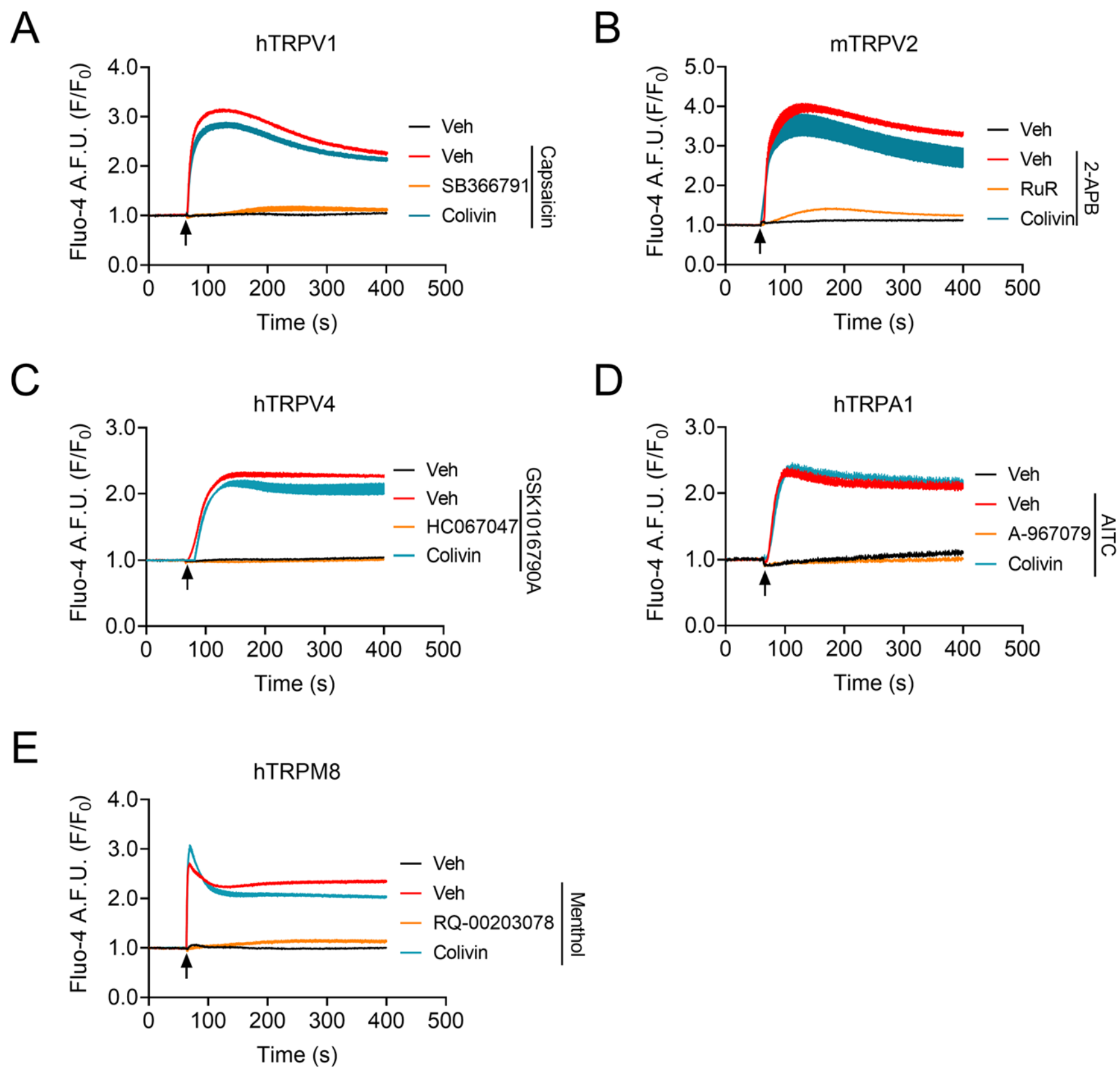

Figure EV1. Effects of Colivin on thermosensitive TRP channels.

Time courses of Ca^{2+} influx induced by capsaicin (1 μM), 2-APB (100 μM), GSK1016790A (10 nM), AITC (30 μM), and menthol (300 μM) in HEK-293 cells expressing hTRPV1 (**A**), mTRPV2 (**B**), hTRPV4 (**C**), hTRPA1 (**D**), and hTRPM8 (**E**), respectively. Colivin (10 μM), along with the corresponding inhibitors: SB366791 (1 μM), ruthenium red (30 μM), HC067047 (1 μM), A-967079 (1 μM), and RQ-00203078 (1 μM), was incubated for 5 min, followed by the addition of agonists at 60 s (black arrow). Each data point represents mean ± SEM (*N* = 3 wells). Each experiment was repeated in three independent cultures with similar results. Related to Fig. 1. Source data are available online for this figure.

© The Author(s)

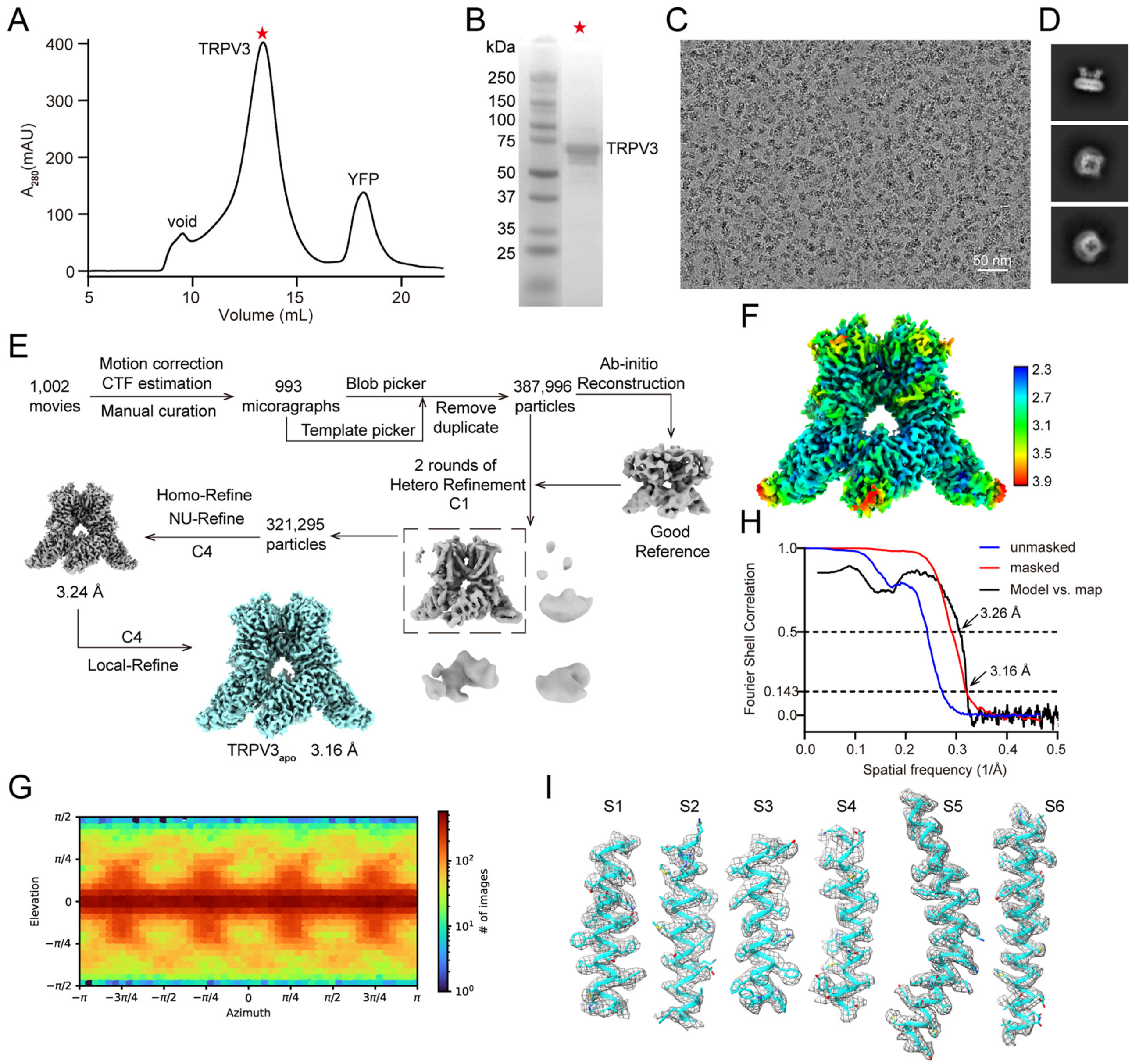

Figure EV2. Protein purification and Cryo-EM reconstruction of TRPV3$_{apo}$.

(**A**) Size-exclusion chromatography of purified human TRPV3. The fractions were pooled for cryo-EM sample preparation. (**B**) SDS-PAGE gel of purified human TRPV3. (**C**) A representative motion-corrected micrograph of the dataset. Scale bar = 50 nm. (**D**) Representative 2D class averages generated from particles for the TRPV3$_{apo}$ map. (**E**) Flow chart of cryo-EM data processing of TRPV3$_{apo}$. Further details can be found in Methods. The contour level of the final map is 0.25. (**F**) Local resolution map of TRPV3$_{apo}$, colored according to the estimated values ranging from 2.3 to 3.9 Å. (**G**) Particle angular distribution calculated in cryoSPARC for the final reconstruction. (**H**) Gold standard Fourier Shell Correlations (FSC) curves of unmasked map (blue), masked map (red), and model versus map (black), marked with resolutions corresponding to FSC = 0.5 and 0.143. (**I**) Representative cryo-EM densities of transmembrane helices (S1-S6) of TRPV3$_{apo}$, contoured at 0.25. Related to Fig. 2.

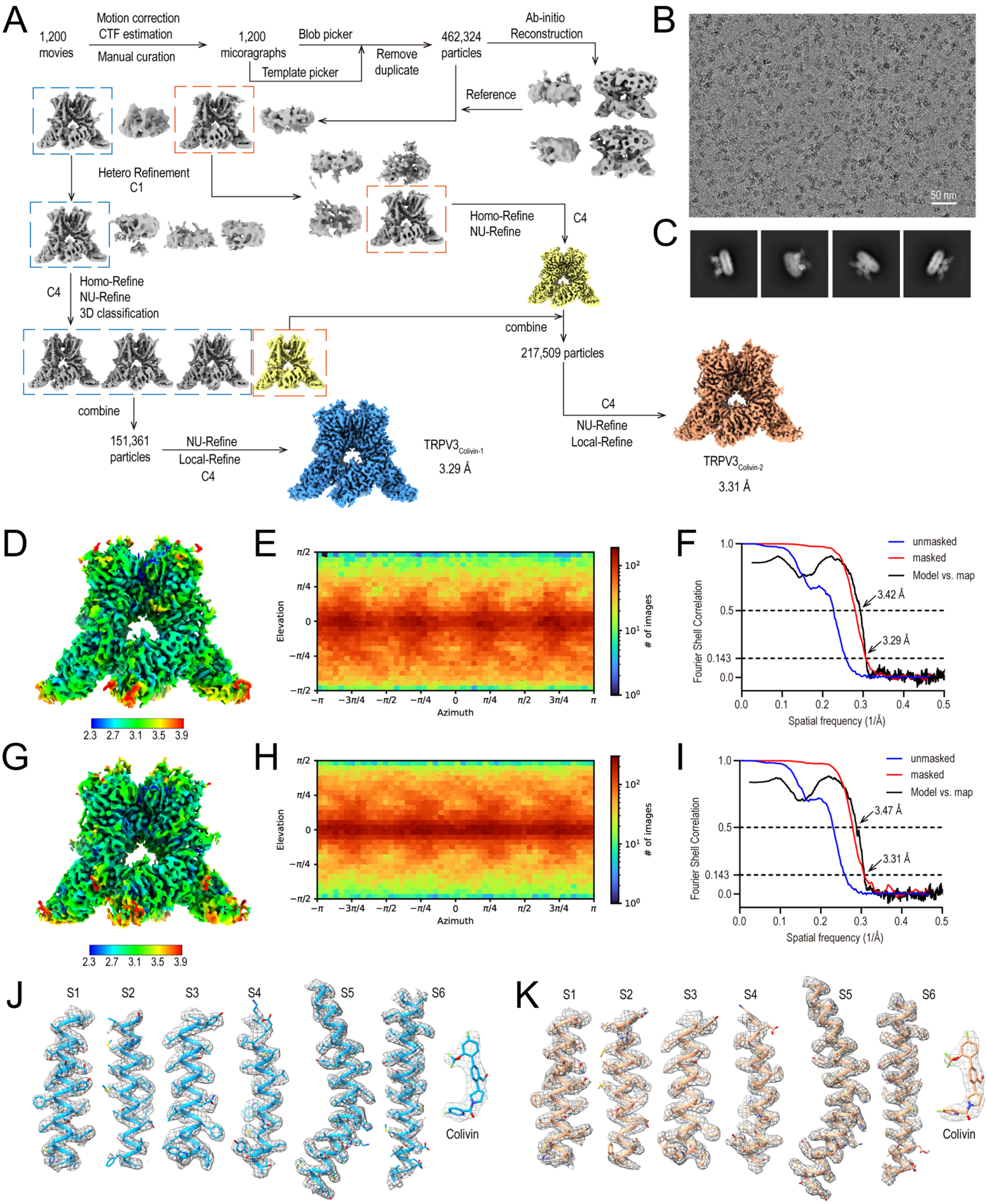
A
1,200 movies
Motion correction
CTF estimation
Manual curation
1,200 micoragraphs
Blob picker
Template picker
Remove duplicate
462,324 particles
Ab-initio Reconstruction
Reference
Hetero Refinement C1
Homo-Refine NU-Refine
C4
Homo-Refine NU-Refine 3D classification
combine
217,509 particles
151,361 particles
NU-Refine Local-Refine C4
TRPV3Colivin-1 3.29 Å
NU-Refine Local-Refine
TRPV3Colivin-2 3.31 Å
B
50 nm
C
D
2.3 2.7 3.1 3.5 3.9
E
Elevation
Azimuth
# of images
F
Fourier Shell Correlation
Spatial frequency (1/Å)
unmasked
masked
Model vs. map
3.42 Å
3.29 Å
G
H
I
3.47 Å
3.31 Å
J
S1 S2 S3 S4 S5 S6
Colivin
K

◀ **Figure EV3. Cryo-EM reconstruction of $TRPV3_{Colivin}$.**

(**A**) Flow chart of cryo-EM data processing of $TRPV3_{Colivin}$. Further details can be found in "Methods". The contour level of the final maps is 0.3. (**B**) A representative motion-corrected micrograph of the dataset. Scale bar = 50 nm. (**C**) Representative 2D class averages generated from particles. (**D**, **G**) Local resolution map of $TRPV3_{Colivin-1}$ (**D**) and $TRPV3_{Colivin-2}$ (**G**), colored according to the estimated values ranging from 2.3 to 3.9 Å. (**E**, **H**) Particle angular distributions calculated in cryoSPARC for the final reconstruction of $TRPV3_{Colivin-1}$ (**E**) and $TRPV3_{Colivin-2}$ (**H**). (**F**, **I**) FSC curves of unmasked map (blue), masked map (red), and model versus map (black) for the final map of $TRPV3_{Colivin-1}$ (**F**) and $TRPV3_{Colivin-2}$ (**I**), marked with resolutions corresponding to FSC = 0.5 and 0.143. (**J**, **K**) Representative cryo-EM densities of transmembrane helices (S1–S6) and ligands for $TRPV3_{Colivin-1}$ (**J**) and $TRPV3_{Colivin-2}$ (**K**), contoured at 0.3. Related to Fig. 2.

© The Author(s)

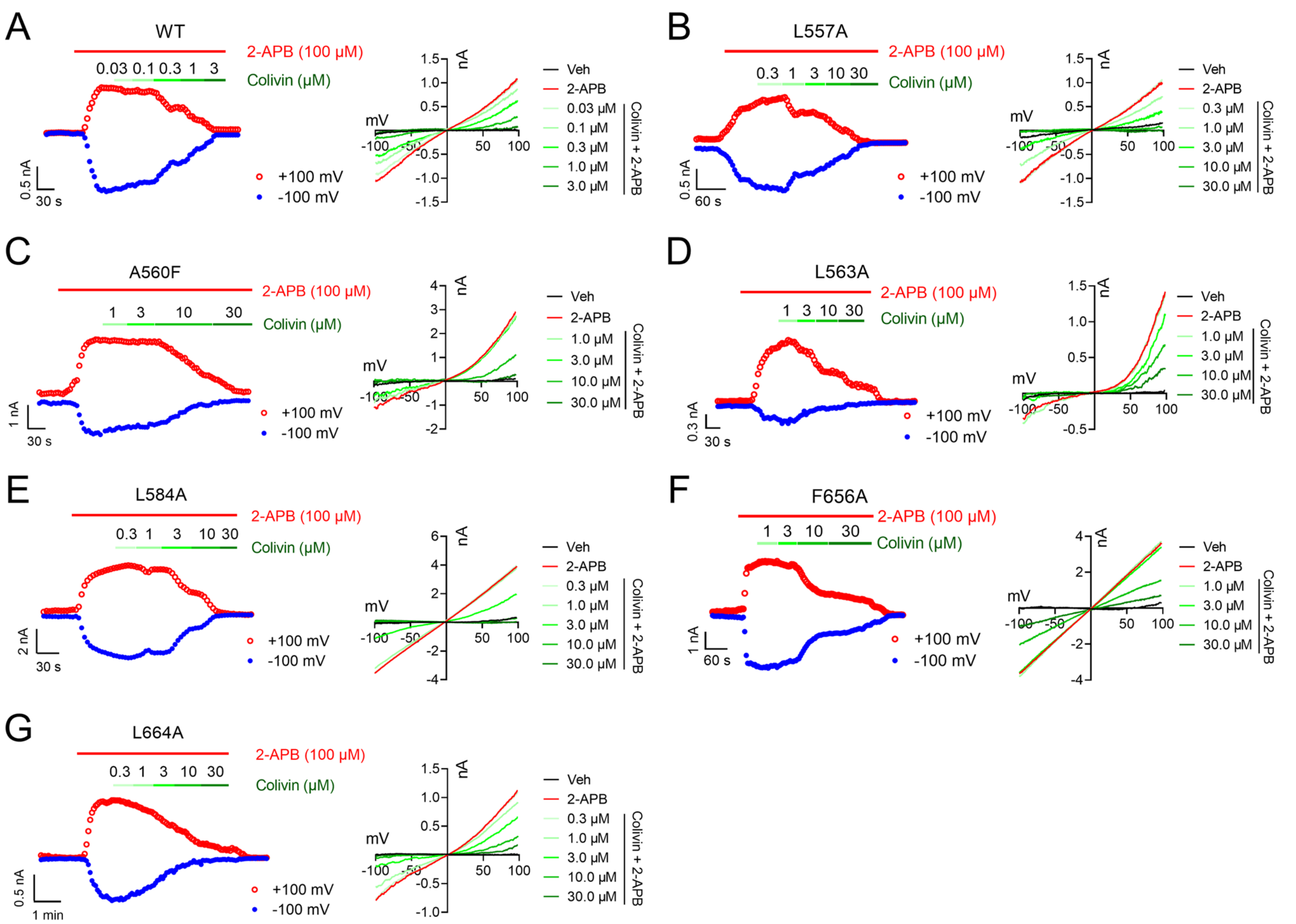

Figure EV4. Mutations in the vanilloid site reduced the apparent affinity of Colivin on hTRPV3.

Effects of different concentrations of Colivin on 2-APB (100 μM)-evoked whole-cell currents at ±100 mV in HEK-293 cells transiently expressing wild-type (WT) hTRPV3 (**A**) and L557A (**B**), A560F (**C**), L563A (**D**), L584A (**E**), F656A (**F**), and L664A (**G**) mutants of hTRPV3. Also shown are representative I–V curves of WT hTRPV3 and its mutants, derived from the different time points of drug applications. Related to Fig. 2. Source data are available online for this figure.

© The Author(s)

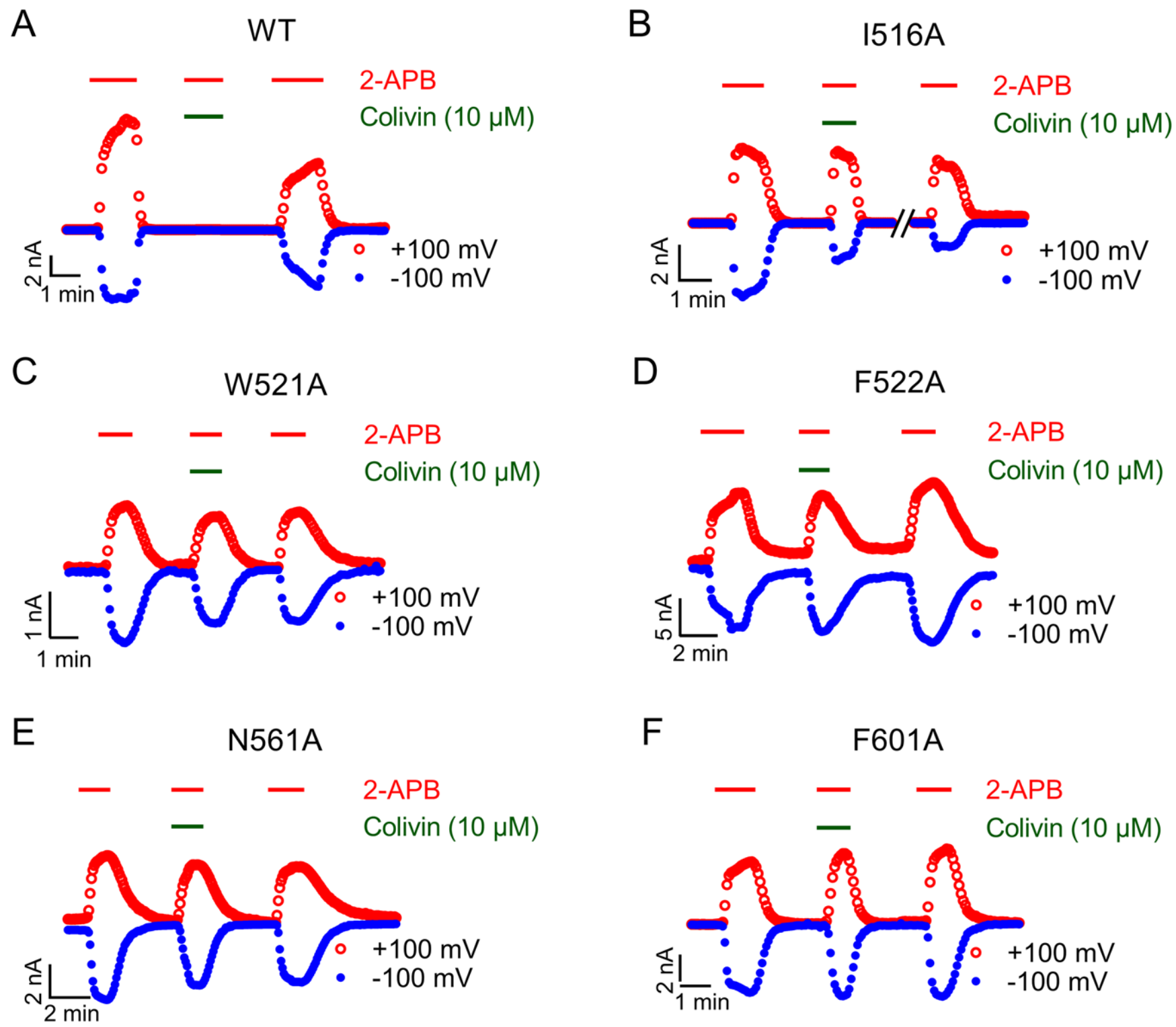

Figure EV5. Colivin inhibition of hTRPV3 was abolished by mutations at critical residues.

Representative whole-cell current traces at ±100 mV with bath applications of 2-APB (100 μM) and Colivin (10 μM) as indicated in HEK-293 cells transiently expressing wild-type (WT) hTRPV3 (**A**) and mutants: I516A (**B**), W521A (**C**), F522A (**D**), N561A (**E**), and F601A (**F**). Related to Fig. 2. Source data are available online for this figure.

© The Author(s)
